# Supplementary material for: Assessing the recovery of an Antarctic predator from historical exploitation
Source: R Soc Open Sci. 2019 Oct 16;6(10):190368. doi: 10.1098/rsos.190368 (PMC6837233; doi:10.1098/rsos.190368)
Supplement: Supplmental Material 1 [file rsos190368supp1.docx]

**Supplementary Material 1**

**Assessing the recovery of an Antarctic predator from historical exploitation**

Alexandre N. Zerbini, Grant Adams, John Best, Phillip J. Clapham, Jennifer A. Jackson and Andre E. Punt

**1) Rationale For Allocation Of Modern Whaling Catches**

For the purpose of allocation of historical catches, the IWC Scientific Committee (SC) defined boundaries for seven Southern Hemisphere humpback whale breeding stocks in the wintering (areas to the north of 40^o^S) and feeding grounds (south of 40^o^S) [1]. Wintering ground catches are allocated to the WSA humpback whale populations, if they were taken south of the Equator, north of 40^o^S and west of 20^o^W. These included individuals taken in the 20^th^ century by catcher boats operating from whaling stations in Costinha (~7^o^S, 35^o^W) and Cabo Frio (~23^o^S, 42^o^W), Brazil [2], and by a Soviet pelagic fleet in the Abrolhos Bank Area (~18^o^30’S, 38^o^30’W) and in offshore areas (~30-32^o^S, 33-36^o^W) along the central South American coast [3, 4].

Feeding ground catches include all catches taken to the south of 40^o^S and were more difficult to assign to breeding stocks because wintering-feeding ground connections are not completely clear for all breeding populations and because mixing may occur in the Southern Ocean. Therefore, the IWC SC developed alternative catch allocation hypotheses (named ‘Naïve’, ‘Fringe’ and ‘Overlap’) to account for possible uncertainties in the feeding ground longitudinal boundaries across all known breeding populations [1]. These boundaries were subsequently reviewed for some populations (e.g., Western South Atlantic, Eastern Indian Ocean and Eastern South Pacific) in light of additional information on migratory connections between low and high latitudes and the former Naïve hypothesis was renamed ‘Core’ (IWC, 2006).

Feeding ground catches included in the WSA humpback whale assessment represent whales taken at South Georgia (54^o^30’S, 36^o^30’W), South Sandwich Islands (56^o^20-59^o^40’S, 21^o^30’W), South Orkney Islands (60^o^35’S, 45^o^30’W), Falkland Islands (59^o^30’S, 51^o^45’W), the Antarctic Peninsula (~65^o^S, 60^o^W) (for the Overlap model only), and pelagic operations in Antarctic waters (Allison, 2006). Areal catch allocation followed the hypotheses proposed by the IWC SC as implemented in a previous assessment of the WSA humpback whales [5]. Retaining the same catch allocation would allow direct comparison of the previous and the updated assessment provided here.

Modern whaling catch allocation is described in Table 1 and illustrated in Fig. 1 (except for the Overlap hypothesis). The Core hypothesis assumes that all catches within the Core area are assigned to the WSA humpbacks. The Falkland Islands catches correspond to humpbacks taken near the Falkland Islands. The migratory connection of whales feeding near the Falkland Islands is unknown. They may include the breeding grounds off Brazil, but the proximity of the Falkland Islands to the southern tip of the South American continent and the presence of humpbacks breeding in the eastern South Pacific in the Magellan strait suggest that humpbacks in the Falkland Islands area could be animals migrating from western South and Central American breeding grounds (Costa Rica, Panama, Colombia, Ecuador and Peru) through the Fuegian channels in southern Chile and Argentina. Thus these catches are considered separately. The Fringe hypothesis assumes that 90% of the catches assigned to the WSA humpbacks come from its Core areas, but 10% come from a ‘fringe’ area between 10^o^ and 20^o^W. This region corresponds to an area where humpbacks wintering off eastern South America could be mixing with humpbacks migrating from the western coast of Africa. The Overlap model assumes that humpbacks in the feeding grounds associated with breeding populations in the eastern South Pacific and eastern South Atlantic (western Africa) could be overlapping with those from the WSA humpbacks. For this reason, this allocation model assigns 80% of the catches in the Core areas for the WSA population and 10% from each of the Core areas associated with the eastern South Pacific (50^o^-100^o^W) and eastern South Atlantic (10^o^W-20^o^E).

Table 1 – Summary of allocation of catches in the feeding grounds (south of 40^o^S) as specified by the IWC [1, 4, 6, 7].

| Core Hypothesis |  | Falkland Catches | Fringe Hypothesis |  | Overlap Hypothesis |  |
| --- | --- | --- | --- | --- | --- | --- |
| Catches between 70-20^o^W of longitude and 40-50^o^S of latitude, plus catches from 50-20^o^W to the south of 50^o^S, excluding the catches in the Falkland Islands |  | Catches taken from 70-50^o^W and between 50 and 58^o^S | 90% of the catches from the Core allocation hypothesis and 10% from a ‘Fringe Area’ between 20 and 10^o^W |  | 80% from Core and 10% from 20^o^W-10^o^E (feeding grounds associated with the eastern South Atlantic) and 10% from 100-50^o^W (feeding grounds associated with the eastern South Pacific) |  |

**References**

1 IWC. 1998 Report of the Scientific Committee. Annex G. Report of the sub-committee on Comprehensive Assessment of Southern Hemisphere humpback whales. Reports of the International Whaling Commission. 48, 170-182.

2 Williamson, G. R. 1975 Minke whales off Brazil. Scientific Reports of the Whales Research Institute, Tokyo. 27, 37-59.

3 Zemsky, V. A., Berzin, A. A., Mikhalev, Y. A., Tormosov, D. D. 1996 Soviet Antarctic whaling data (1947-1972). 2nd ed. Moscow: Center for Russian Environment Policy.

4 Allison, C. 2006 Documentation of the creation of the Southern Hemisphere humpback catch series, February 2006, Cambridge, UK.

5 Zerbini, A. N., Ward, E., Engel, M., Andriolo, A., Kinas, P. G. 2011 A Bayesian assessment of the conservation status of humpback whales (Megaptera novaeangliae) in the western South Atlantic Ocean (Breeding Stock A). J. Cetacean Res. Manage. (special issue 3). 131-144.

6 IWC. Report of the Scientific Committee. Annex H. Report of the Sub-Committee on the Other Southern Hemisphere Whale Stocks. In: International Whaling Commission, ed. Journal of Cetacean Research and Management (Supplement) 2006:151-170.

7 IWC. 2011 Report of the Workshop on the Comprehensive Assessment of Southern Hemisphere humpback whales, 4-7 April 2006, Hobart, Tasmania. J. Cetacean Res. Manage. (special issue 3). 1-50.


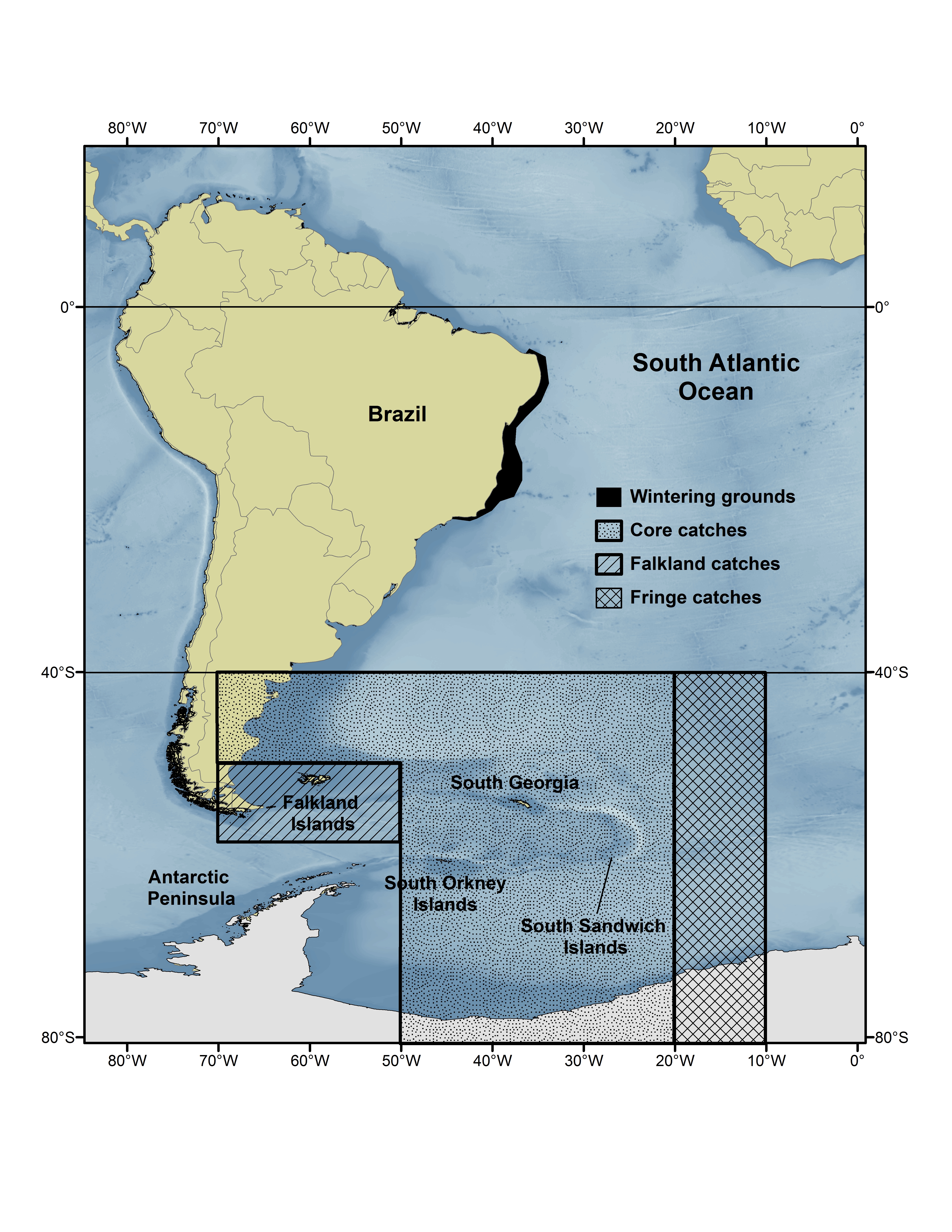


Fig. 1 – Modern whaling catch allocation areas in the breeding (north of 40^o^S) and feeding grounds (south of 40^o^S) associated with the WSA humpback whale population.
