# Supplementary material for: Assessing the recovery of an Antarctic predator from historical exploitation
Source: R Soc Open Sci. 2019 Oct 16;6(10):190368. doi: 10.1098/rsos.190368 (PMC6837233; doi:10.1098/rsos.190368)

**Supplementary Material 2**

**Assessing the recovery of an Antarctic predator from historical exploitation**

Alexandre N. Zerbini, Grant Adams, John Best, Phillip J. Clapham, Jennifer A. Jackson and Andre E. Punt

**1) Bayes factor (model likelihood) comparison of scenarios**

| **Scenario** | **Bayes Factor (weight)** |  |  |
| --- | --- | --- | --- |
| Reference | 0.099 |  |  |
| D-1 | 0.101 |  |  |
| D-7 | 0.100 |  |  |
| C-4 | 0.131 |  |  |
| C-5 | 0.102 |  |  |
| C-6 | 0.100 |  |  |
| C-7 | 0.036 |  |  |
| G-1 | 0.149 |  |  |
| G- 2 | 0.183 |  |  |
| M-1 | 0.099 |  |  |
| M-2 | 0.101 |  |  |

**2) Estimation of Model Parameters and Quantities of Interest for the Reference Case and Sensitivity Scenarios**

# 2.1) Reference Case

| Parameter | Mean | | Median | | 2.5% PI | | 25% PI | | 75% PI | | 97.5% PI | | Unique | |
| --- | --- | --- | --- | --- | --- | --- | --- | --- | --- | --- | --- | --- | --- | --- |
| *r_max_* | | 0.091 | | 0.094 | | 0.054 | | 0.08 | | 0.106 | | 0.117 | | 10,000 |
| *K* | | 28,198 | | 27,726 | | 25,296 | | 26,658 | | 29,233 | | 33,606 | | 10,000 |
| N_min_ | | 477 | | 374 | | 193 | | 281 | | 563 | | 1,312 | | 10,000 |
| N_2006_ | | 13,005 | | 12,933 | | 11,169 | | 12,300 | | 13,669 | | 15,141 | | 10,000 |
| N_2008_ | | 15,015 | | 14,979 | | 13,284 | | 14,370 | | 15,634 | | 16,925 | | 10,000 |
| N_2012_ | | 19,231 | | 19,237 | | 17,371 | | 18,604 | | 19,857 | | 21,037 | | 10,000 |
| N_2019_ | | 24,866 | | 24,892 | | 23,088 | | 24,358 | | 25,427 | | 26,367 | | 10,000 |
| N_2030_ | | 27,651 | | 27,471 | | 25,259 | | 26,545 | | 28,606 | | 30,823 | | 10,000 |
| Maximum depletion | | 0.016 | | 0.013 | | 0.007 | | 0.011 | | 0.019 | | 0.04 | | 10,000 |
| Status in 2006 | | 0.462 | | 0.461 | | 0.396 | | 0.438 | | 0.486 | | 0.537 | | 10,000 |
| Status in 2008 | | 0.535 | | 0.535 | | 0.449 | | 0.506 | | 0.564 | | 0.621 | | 10,000 |
| Status in 2012 | | 0.686 | | 0.693 | | 0.546 | | 0.647 | | 0.732 | | 0.794 | | 10,000 |
| Status in 2019 | | 0.887 | | 0.906 | | 0.713 | | 0.855 | | 0.937 | | 0.964 | | 10,000 |
| Status in 2030 | | 0.983 | | 0.993 | | 0.903 | | 0.981 | | 0.997 | | 0.999 | | 10,000 |

# 2.2) Scenario D-1

| Parameter | Mean | | Median | | 2.5% PI | | 25% PI | | 75% PI | | 97.5% PI | | Unique | |
| --- | --- | --- | --- | --- | --- | --- | --- | --- | --- | --- | --- | --- | --- | --- |
| *r_max_* | | 0.091 | | 0.092 | | 0.054 | | 0.079 | | 0.104 | | 0.116 | | 10,000 |
| *K* | | 28,317 | | 27,872 | | 25,300 | | 26,735 | | 29,376 | | 33,931 | | 10,000 |
| N_min_ | | 485 | | 384 | | 194 | | 288 | | 569 | | 1,310 | | 10,000 |
| N_2006_ | | 12,983 | | 12,925 | | 11,156 | | 12,293 | | 13,617 | | 15,134 | | 10,000 |
| N_2008_ | | 14,979 | | 14,956 | | 13,262 | | 14,336 | | 15,574 | | 16,862 | | 10,000 |
| N_2012_ | | 19,180 | | 19,174 | | 17,394 | | 18,567 | | 19,801 | | 20,974 | | 10,000 |
| N_2019_ | | 24,867 | | 24,899 | | 23,149 | | 24,357 | | 25,434 | | 26,332 | | 10,000 |
| N_2030_ | | 27,738 | | 27,592 | | 25,254 | | 26,616 | | 28,724 | | 30,903 | | 10,000 |
| Maximum depletion | | 0.017 | | 0.014 | | 0.007 | | 0.011 | | 0.019 | | 0.04 | | 10,000 |
| Status in 2006 | | 0.46 | | 0.459 | | 0.395 | | 0.436 | | 0.482 | | 0.53 | | 10,000 |
| Status in 2008 | | 0.531 | | 0.532 | | 0.447 | | 0.503 | | 0.56 | | 0.616 | | 10,000 |
| Status in 2012 | | 0.682 | | 0.687 | | 0.543 | | 0.642 | | 0.727 | | 0.79 | | 10,000 |
| Status in 2019 | | 0.883 | | 0.901 | | 0.708 | | 0.851 | | 0.933 | | 0.963 | | 10,000 |
| Status in 2030 | | 0.982 | | 0.992 | | 0.899 | | 0.981 | | 0.997 | | 0.999 | | 10,000 |

# 2.3) Scenario D-2

| Parameter | Mean | Median | 2.5% PI | 25% PI | 75% PI | 97.5% PI | Unique |
| --- | --- | --- | --- | --- | --- | --- | --- |
| *r_max_* | 0.087 | 0.092 | 0.033 | 0.073 | 0.106 | 0.117 | 10,000 |
| *K* | 28,960 | 27,813 | 25,243 | 26,585 | 29,780 | 40,018 | 10,000 |
| N_min_ | 682 | 395 | 194 | 281 | 717 | 2,763 | 10,000 |
| N_2006_ | 13,387 | 13,282 | 11,331 | 12,511 | 14,129 | 16,043 | 10,000 |
| N_2008_ | 15,327 | 15,287 | 13,446 | 14,608 | 15,991 | 17,457 | 10,000 |
| N_2012_ | 19,353 | 19,352 | 17,392 | 18,684 | 20,026 | 21,311 | 10,000 |
| N_2019_ | 24,688 | 24,812 | 21,712 | 24,249 | 25,384 | 26,367 | 10,000 |
| N_2030_ | 27,579 | 27,415 | 25,068 | 26,421 | 28,595 | 31,024 | 10,000 |
| Maximum depletion | 0.021 | 0.014 | 0.007 | 0.011 | 0.024 | 0.071 | 10,000 |
| Status in 2006 | 0.467 | 0.468 | 0.375 | 0.44 | 0.495 | 0.551 | 10,000 |
| Status in 2008 | 0.536 | 0.54 | 0.402 | 0.506 | 0.574 | 0.636 | 10,000 |
| Status in 2012 | 0.68 | 0.696 | 0.459 | 0.637 | 0.741 | 0.804 | 10,000 |
| Status in 2019 | 0.868 | 0.905 | 0.557 | 0.835 | 0.941 | 0.967 | 10,000 |
| Status in 2030 | 0.964 | 0.993 | 0.712 | 0.973 | 0.997 | 0.999 | 10,000 |

# 2.4) Scenario D-3

| Parameter | Mean | Median | 2.5% PI | 25% PI | 75% PI | 97.5% PI | Unique |
| --- | --- | --- | --- | --- | --- | --- | --- |
| *r_max_* | 0.107 | 0.109 | 0.084 | 0.102 | 0.114 | 0.118 | 10,000 |
| *K* | 26,728 | 26,621 | 25,040 | 25,928 | 27,331 | 29,322 | 10,000 |
| N_min_ | 275 | 257 | 177 | 216 | 305 | 497 | 10,000 |
| N_2006_ | 12,404 | 12,363 | 10,807 | 11,797 | 12,971 | 14,245 | 10,000 |
| N_2008_ | 14,660 | 14,627 | 12,989 | 14,024 | 15,256 | 16,529 | 10,000 |
| N_2012_ | 19,367 | 19,357 | 17,696 | 18,761 | 19,952 | 21,084 | 10,000 |
| N_2019_ | 24,922 | 24,913 | 23,759 | 24,445 | 25,379 | 26,148 | 10,000 |
| N_2030_ | 26,620 | 26,546 | 25,006 | 25,873 | 27,221 | 28,931 | 10,000 |
| Maximum depletion | 0.01 | 0.01 | 0.007 | 0.008 | 0.011 | 0.018 | 10,000 |
| Status in 2006 | 0.465 | 0.462 | 0.403 | 0.44 | 0.487 | 0.538 | 10,000 |
| Status in 2008 | 0.549 | 0.548 | 0.478 | 0.522 | 0.575 | 0.629 | 10,000 |
| Status in 2012 | 0.726 | 0.727 | 0.636 | 0.697 | 0.756 | 0.806 | 10,000 |
| Status in 2019 | 0.933 | 0.94 | 0.859 | 0.921 | 0.952 | 0.968 | 10,000 |
| Status in 2030 | 0.996 | 0.998 | 0.984 | 0.996 | 0.998 | 0.999 | 10,000 |

# 2.5) Scenario D-4

| Parameter | Mean | | Median | | 2.5% PI | | 25% PI | | 75% PI | | 97.5% PI | | Unique | |
| --- | --- | --- | --- | --- | --- | --- | --- | --- | --- | --- | --- | --- | --- | --- |
| *r_max_* | | 0.094 | | 0.097 | | 0.056 | | 0.083 | | 0.108 | | 0.117 | | 10,000 |
| *K* | | 27,957 | | 27,492 | | 25,276 | | 26,496 | | 28,896 | | 33,348 | | 10,000 |
| N_min_ | | 440 | | 342 | | 190 | | 268 | | 504 | | 1,243 | | 10,000 |
| N_2006_ | | 12,907 | | 12,846 | | 11,148 | | 12,198 | | 13,552 | | 15,010 | | 10,000 |
| N_2008_ | | 14,961 | | 14,941 | | 13,269 | | 14,320 | | 15,569 | | 16,842 | | 10,000 |
| N_2012_ | | 19,263 | | 19,266 | | 17,508 | | 18,648 | | 19,882 | | 21,037 | | 10,000 |
| N_2019_ | | 24,893 | | 24,915 | | 23,334 | | 24,394 | | 25,431 | | 26,305 | | 10,000 |
| N_2030_ | | 27,487 | | 27,304 | | 25,236 | | 26,403 | | 28,393 | | 30,731 | | 10,000 |
| Maximum depletion | | 0.015 | | 0.013 | | 0.007 | | 0.01 | | 0.018 | | 0.038 | | 10,000 |
| Status in 2006 | | 0.463 | | 0.462 | | 0.396 | | 0.438 | | 0.486 | | 0.536 | | 10,000 |
| Status in 2008 | | 0.537 | | 0.538 | | 0.451 | | 0.509 | | 0.566 | | 0.623 | | 10,000 |
| Status in 2012 | | 0.693 | | 0.7 | | 0.553 | | 0.655 | | 0.737 | | 0.797 | | 10,000 |
| Status in 2019 | | 0.895 | | 0.914 | | 0.726 | | 0.868 | | 0.941 | | 0.965 | | 10,000 |
| Status in 2030 | | 0.985 | | 0.994 | | 0.913 | | 0.985 | | 0.997 | | 0.999 | | 10,000 |

# 2.6) Scenario D-5

| Parameter | Mean | Median | 2.5% PI | 25% PI | 75% PI | 97.5% PI | Unique |
| --- | --- | --- | --- | --- | --- | --- | --- |
| *r_max_* | 0.108 | 0.11 | 0.086 | 0.104 | 0.115 | 0.118 | 10,000 |
| *K* | 26,637 | 26,534 | 25,049 | 25,885 | 27,200 | 29,046 | 10,000 |
| N_min_ | 264 | 248 | 177 | 211 | 292 | 466 | 10,000 |
| N_2006_ | 12,378 | 12,343 | 10,782 | 11,772 | 12,943 | 14,185 | 10,000 |
| N_2008_ | 14,654 | 14,624 | 12,972 | 14,025 | 15,244 | 16,503 | 10,000 |
| N_2012_ | 19,398 | 19,391 | 17,702 | 18,810 | 19,990 | 21,097 | 10,000 |
| N_2019_ | 24,924 | 24,924 | 23,769 | 24,450 | 25,378 | 26,157 | 10,000 |
| N_2030_ | 26,543 | 26,471 | 25,013 | 25,834 | 27,107 | 28,680 | 10,000 |
| Maximum depletion | 0.01 | 0.009 | 0.007 | 0.008 | 0.011 | 0.016 | 10,000 |
| Status in 2006 | 0.465 | 0.464 | 0.403 | 0.441 | 0.487 | 0.537 | 10,000 |
| Status in 2008 | 0.551 | 0.55 | 0.48 | 0.524 | 0.576 | 0.629 | 10,000 |
| Status in 2012 | 0.729 | 0.731 | 0.642 | 0.702 | 0.758 | 0.807 | 10,000 |
| Status in 2019 | 0.936 | 0.942 | 0.87 | 0.926 | 0.954 | 0.969 | 10,000 |
| Status in 2030 | 0.997 | 0.998 | 0.987 | 0.996 | 0.998 | 0.999 | 10,000 |

# 2.7) Scenario D-6

| Parameter | Mean | Median | 2.5% PI | 25% PI | 75% PI | 97.5% PI | Unique |
| --- | --- | --- | --- | --- | --- | --- | --- |
| *r_max_* | 0.084 | 0.087 | 0.031 | 0.068 | 0.103 | 0.117 | 10,000 |
| *K* | 29,382 | 28,161 | 25,275 | 26,814 | 30,436 | 40,897 | 10,000 |
| N_min_ | 762 | 447 | 198 | 301 | 865 | 2,969 | 10,000 |
| N_2006_ | 13,516 | 13,400 | 11,335 | 12,633 | 14,304 | 16,263 | 10,000 |
| N_2008_ | 15,390 | 15,329 | 13,447 | 14,650 | 16,085 | 17,599 | 10,000 |
| N_2012_ | 19,292 | 19,284 | 17,350 | 18,617 | 19,955 | 21,282 | 10,000 |
| N_2019_ | 24,616 | 24,758 | 21,534 | 24,162 | 25,331 | 26,378 | 10,000 |
| N_2030_ | 27,767 | 27,622 | 25,128 | 26,611 | 28,834 | 31,218 | 10,000 |
| Maximum depletion | 0.023 | 0.016 | 0.008 | 0.011 | 0.029 | 0.075 | 10,000 |
| Status in 2006 | 0.465 | 0.466 | 0.371 | 0.439 | 0.493 | 0.548 | 10,000 |
| Status in 2008 | 0.531 | 0.535 | 0.396 | 0.5 | 0.568 | 0.631 | 10,000 |
| Status in 2012 | 0.669 | 0.684 | 0.448 | 0.621 | 0.732 | 0.801 | 10,000 |
| Status in 2019 | 0.854 | 0.89 | 0.54 | 0.81 | 0.934 | 0.965 | 10,000 |
| Status in 2030 | 0.958 | 0.989 | 0.687 | 0.961 | 0.997 | 0.999 | 10,000 |

# 2.8) Scenario D-7

| Parameter | Mean | Median | 2.5% PI | 25% PI | 75% PI | 97.5% PI | Unique |
| --- | --- | --- | --- | --- | --- | --- | --- |
| *r_max_* | 0.088 | 0.088 | 0.063 | 0.079 | 0.097 | 0.113 | 9,999 |
| *K* | 28,433 | 28,230 | 25,688 | 27,215 | 29,440 | 32,276 | 10,000 |
| N_min_ | 486 | 441 | 222 | 344 | 579 | 1,001 | 10,000 |
| N_2006_ | 13,104 | 13,070 | 11,339 | 12,465 | 13,724 | 14,997 | 10,000 |
| N_2008_ | 15,059 | 15,028 | 13,341 | 14,432 | 15,669 | 16,910 | 10,000 |
| N_2012_ | 19,178 | 19,173 | 17,376 | 18,558 | 19,794 | 21,029 | 10,000 |
| N_2019_ | 24,946 | 24,953 | 23,383 | 24,408 | 25,490 | 26,416 | 10,000 |
| N_2030_ | 27,968 | 27,871 | 25,615 | 27,014 | 28,871 | 30,656 | 10,000 |
| Maximum depletion | 0.017 | 0.016 | 0.008 | 0.013 | 0.02 | 0.032 | 10,000 |
| Status in 2006 | 0.462 | 0.46 | 0.398 | 0.437 | 0.485 | 0.533 | 10,000 |
| Status in 2008 | 0.531 | 0.53 | 0.455 | 0.503 | 0.558 | 0.612 | 10,000 |
| Status in 2012 | 0.677 | 0.678 | 0.571 | 0.641 | 0.715 | 0.777 | 10,000 |
| Status in 2019 | 0.88 | 0.888 | 0.762 | 0.849 | 0.918 | 0.956 | 10,000 |
| Status in 2030 | 0.984 | 0.989 | 0.942 | 0.98 | 0.994 | 0.998 | 10,000 |

# 2.9) Scenario C-1

| Parameter | Mean | Median | 2.5% PI | 25% PI | 75% PI | 97.5% PI | Unique |
| --- | --- | --- | --- | --- | --- | --- | --- |
| *r_max_* | 0.093 | 0.096 | 0.055 | 0.082 | 0.107 | 0.117 | 10,000 |
| *K* | 23,339 | 23,152 | 22,246 | 22,647 | 23,825 | 25,501 | 10,000 |
| N_min_ | 463 | 358 | 200 | 273 | 537 | 1,275 | 10,000 |
| N_2006_ | 13,194 | 13,125 | 11,286 | 12,442 | 13,873 | 15,449 | 10,000 |
| N_2008_ | 15,007 | 14,969 | 13,265 | 14,348 | 15,625 | 16,987 | 10,000 |
| N_2012_ | 18,393 | 18,408 | 16,918 | 17,877 | 18,898 | 19,829 | 10,000 |
| N_2019_ | 21,897 | 21,898 | 21,140 | 21,713 | 22,109 | 22,555 | 10,000 |
| N_2030_ | 23,152 | 23,078 | 22,228 | 22,616 | 23,633 | 24,402 | 10,000 |
| Maximum depletion | 0.019 | 0.015 | 0.009 | 0.012 | 0.023 | 0.05 | 10,000 |
| Status in 2006 | 0.565 | 0.564 | 0.496 | 0.538 | 0.59 | 0.645 | 10,000 |
| Status in 2008 | 0.643 | 0.642 | 0.571 | 0.615 | 0.67 | 0.725 | 10,000 |
| Status in 2012 | 0.789 | 0.793 | 0.69 | 0.76 | 0.823 | 0.87 | 10,000 |
| Status in 2019 | 0.94 | 0.951 | 0.838 | 0.923 | 0.967 | 0.98 | 10,000 |
| Status in 2030 | 0.992 | 0.997 | 0.956 | 0.992 | 0.999 | 0.999 | 10,000 |

# 2.10) Scenario C-2

| Parameter | Mean | Median | 2.5% PI | 25% PI | 75% PI | 97.5% PI | Unique |
| --- | --- | --- | --- | --- | --- | --- | --- |
| *r_max_* | 0.094 | 0.096 | 0.056 | 0.083 | 0.108 | 0.117 | 10,000 |
| *K* | 23,745 | 23,556 | 22,653 | 23,059 | 24,225 | 25,858 | 10,000 |
| N_min_ | 454 | 356 | 200 | 274 | 523 | 1,251 | 10,000 |
| N_2006_ | 13,130 | 13,078 | 11,290 | 12,389 | 13,803 | 15,342 | 10,000 |
| N_2008_ | 14,982 | 14,958 | 13,270 | 14,327 | 15,608 | 16,886 | 10,000 |
| N_2012_ | 18,493 | 18,505 | 16,966 | 17,974 | 19,028 | 19,924 | 10,000 |
| N_2019_ | 22,210 | 22,227 | 21,350 | 22,030 | 22,431 | 22,860 | 10,000 |
| N_2030_ | 23,552 | 23,480 | 22,635 | 23,024 | 24,022 | 24,780 | 10,000 |
| Maximum depletion | 0.019 | 0.015 | 0.009 | 0.012 | 0.022 | 0.048 | 10,000 |
| Status in 2006 | 0.553 | 0.551 | 0.486 | 0.527 | 0.577 | 0.628 | 10,000 |
| Status in 2008 | 0.631 | 0.63 | 0.559 | 0.604 | 0.657 | 0.71 | 10,000 |
| Status in 2012 | 0.78 | 0.784 | 0.68 | 0.751 | 0.813 | 0.861 | 10,000 |
| Status in 2019 | 0.937 | 0.948 | 0.835 | 0.921 | 0.964 | 0.979 | 10,000 |
| Status in 2030 | 0.992 | 0.997 | 0.957 | 0.992 | 0.998 | 0.999 | 10,000 |

# 2.11) Scenario C-3

| Parameter | Mean | Median | 2.5% PI | 25% PI | 75% PI | 97.5% PI | Unique |
| --- | --- | --- | --- | --- | --- | --- | --- |
| *r_max_* | 0.092 | 0.095 | 0.054 | 0.081 | 0.106 | 0.117 | 10,000 |
| *K* | 25,691 | 25,359 | 23,775 | 24,609 | 26,387 | 29,421 | 10,000 |
| N_min_ | 468 | 361 | 197 | 275 | 537 | 1,290 | 10,000 |
| N_2006_ | 13,076 | 13,025 | 11,246 | 12,358 | 13,728 | 15,263 | 10,000 |
| N_2008_ | 15,008 | 14,980 | 13,267 | 14,368 | 15,608 | 16,919 | 10,000 |
| N_2012_ | 18,861 | 18,869 | 17,196 | 18,309 | 19,422 | 20,475 | 10,000 |
| N_2019_ | 23,426 | 23,448 | 22,224 | 23,120 | 23,773 | 24,428 | 10,000 |
| N_2030_ | 25,349 | 25,219 | 23,746 | 24,554 | 26,026 | 27,536 | 10,000 |
| Maximum depletion | 0.018 | 0.014 | 0.008 | 0.011 | 0.02 | 0.044 | 10,000 |
| Status in 2006 | 0.509 | 0.508 | 0.446 | 0.485 | 0.532 | 0.582 | 10,000 |
| Status in 2008 | 0.585 | 0.585 | 0.507 | 0.558 | 0.612 | 0.668 | 10,000 |
| Status in 2012 | 0.737 | 0.742 | 0.614 | 0.703 | 0.778 | 0.831 | 10,000 |
| Status in 2019 | 0.915 | 0.931 | 0.774 | 0.892 | 0.953 | 0.972 | 10,000 |
| Status in 2030 | 0.988 | 0.995 | 0.931 | 0.988 | 0.998 | 0.999 | 10,000 |

# 2.12) Scenario C-4

| Parameter | Mean | Median | 2.5% PI | 25% PI | 75% PI | 97.5% PI | Unique |
| --- | --- | --- | --- | --- | --- | --- | --- |
| *r_max_* | 0.091 | 0.093 | 0.055 | 0.08 | 0.105 | 0.117 | 10,000 |
| *K* | 30,038 | 29,591 | 26,072 | 28,144 | 31,363 | 36,891 | 10,000 |
| N_min_ | 471 | 372 | 192 | 282 | 555 | 1,271 | 10,000 |
| N_2006_ | 12,925 | 12,863 | 11,132 | 12,231 | 13,559 | 15,072 | 10,000 |
| N_2008_ | 14,985 | 14,948 | 13,252 | 14,349 | 15,590 | 16,901 | 10,000 |
| N_2012_ | 19,442 | 19,431 | 17,535 | 18,752 | 20,114 | 21,427 | 10,000 |
| N_2019_ | 25,879 | 25,854 | 23,555 | 25,079 | 26,616 | 28,495 | 10,000 |
| N_2030_ | 29,348 | 29,186 | 26,005 | 27,955 | 30,486 | 34,105 | 10,000 |
| Maximum depletion | 0.015 | 0.013 | 0.007 | 0.01 | 0.018 | 0.037 | 10,000 |
| Status in 2006 | 0.433 | 0.432 | 0.353 | 0.407 | 0.458 | 0.513 | 10,000 |
| Status in 2008 | 0.502 | 0.503 | 0.405 | 0.472 | 0.533 | 0.596 | 10,000 |
| Status in 2012 | 0.653 | 0.658 | 0.506 | 0.61 | 0.7 | 0.77 | 10,000 |
| Status in 2019 | 0.867 | 0.886 | 0.68 | 0.831 | 0.922 | 0.956 | 10,000 |
| Status in 2030 | 0.979 | 0.991 | 0.888 | 0.977 | 0.996 | 0.999 | 10,000 |

# 2.13) Scenario C-5

| Parameter | Mean | Median | 2.5% PI | 25% PI | 75% PI | 97.5% PI | Unique |
| --- | --- | --- | --- | --- | --- | --- | --- |
| *r_max_* | 0.092 | 0.094 | 0.055 | 0.08 | 0.105 | 0.117 | 10,000 |
| *K* | 28,312 | 27,873 | 25,460 | 26,807 | 29,358 | 33,768 | 10,000 |
| N_min_ | 502 | 411 | 194 | 300 | 600 | 1,287 | 10,000 |
| N_2006_ | 12,984 | 12,910 | 11,199 | 12,257 | 13,641 | 15,133 | 10,000 |
| N_2008_ | 15,001 | 14,968 | 13,276 | 14,333 | 15,618 | 16,930 | 10,000 |
| N_2012_ | 19,244 | 19,240 | 17,393 | 18,613 | 19,882 | 21,076 | 10,000 |
| N_2019_ | 24,954 | 24,980 | 23,193 | 24,449 | 25,520 | 26,413 | 10,000 |
| N_2030_ | 27,773 | 27,606 | 25,420 | 26,692 | 28,718 | 30,958 | 10,000 |
| Maximum depletion | 0.017 | 0.015 | 0.007 | 0.011 | 0.021 | 0.039 | 10,000 |
| Status in 2006 | 0.46 | 0.459 | 0.394 | 0.436 | 0.483 | 0.533 | 10,000 |
| Status in 2008 | 0.532 | 0.532 | 0.447 | 0.504 | 0.561 | 0.618 | 10,000 |
| Status in 2012 | 0.684 | 0.689 | 0.547 | 0.644 | 0.729 | 0.79 | 10,000 |
| Status in 2019 | 0.886 | 0.903 | 0.716 | 0.855 | 0.935 | 0.963 | 10,000 |
| Status in 2030 | 0.983 | 0.993 | 0.905 | 0.982 | 0.997 | 0.999 | 10,000 |

# 2.14) Scenario C-6

| Parameter | Mean | Median | 2.5% PI | 25% PI | 75% PI | 97.5% PI | Unique |
| --- | --- | --- | --- | --- | --- | --- | --- |
| *r_max_* | 0.092 | 0.094 | 0.055 | 0.08 | 0.105 | 0.117 | 10,000 |
| *K* | 28,239 | 27,780 | 25,328 | 26,691 | 29,265 | 33,749 | 10,000 |
| N_min_ | 496 | 393 | 195 | 296 | 577 | 1,439 | 10,000 |
| N_2006_ | 13,006 | 12,948 | 11,181 | 12,303 | 13,653 | 15,142 | 10,000 |
| N_2008_ | 15,022 | 14,989 | 13,271 | 14,380 | 15,636 | 16,953 | 10,000 |
| N_2012_ | 19,251 | 19,257 | 17,441 | 18,626 | 19,877 | 21,066 | 10,000 |
| N_2019_ | 24,911 | 24,923 | 23,263 | 24,405 | 25,462 | 26,384 | 10,000 |
| N_2030_ | 27,696 | 27,529 | 25,286 | 26,582 | 28,686 | 30,831 | 10,000 |
| Maximum depletion | 0.017 | 0.014 | 0.007 | 0.011 | 0.02 | 0.044 | 10,000 |
| Status in 2006 | 0.462 | 0.461 | 0.395 | 0.437 | 0.485 | 0.534 | 10,000 |
| Status in 2008 | 0.534 | 0.535 | 0.447 | 0.505 | 0.564 | 0.62 | 10,000 |
| Status in 2012 | 0.686 | 0.692 | 0.546 | 0.647 | 0.731 | 0.792 | 10,000 |
| Status in 2019 | 0.887 | 0.905 | 0.711 | 0.856 | 0.936 | 0.963 | 10,000 |
| Status in 2030 | 0.983 | 0.993 | 0.903 | 0.981 | 0.997 | 0.999 | 10,000 |

# 2.15) Scenario C-7

| Parameter | Mean | Median | 2.5% PI | 25% PI | 75% PI | 97.5% PI | Unique |
| --- | --- | --- | --- | --- | --- | --- | --- |
| *r_max_* | 0.089 | 0.091 | 0.05 | 0.076 | 0.103 | 0.116 | 9,999 |
| *K* | 25,479 | 24,914 | 22,184 | 23,727 | 26,639 | 31,934 | 10,000 |
| N_min_ | 617 | 531 | 204 | 333 | 803 | 1,491 | 10,000 |
| N_2006_ | 13,250 | 13,193 | 11,379 | 12,507 | 13,933 | 15,457 | 10,000 |
| N_2008_ | 15,077 | 15,054 | 13,339 | 14,403 | 15,689 | 17,023 | 10,000 |
| N_2012_ | 18,661 | 18,655 | 17,005 | 18,099 | 19,222 | 20,314 | 10,000 |
| N_2019_ | 22,937 | 22,927 | 21,356 | 22,325 | 23,516 | 24,647 | 10,000 |
| N_2030_ | 24,985 | 24,734 | 22,159 | 23,657 | 26,113 | 28,973 | 10,000 |
| Maximum depletion | 0.023 | 0.021 | 0.009 | 0.014 | 0.03 | 0.048 | 10,000 |
| Status in 2006 | 0.523 | 0.522 | 0.434 | 0.493 | 0.553 | 0.616 | 10,000 |
| Status in 2008 | 0.596 | 0.598 | 0.48 | 0.561 | 0.633 | 0.7 | 10,000 |
| Status in 2012 | 0.739 | 0.749 | 0.571 | 0.697 | 0.791 | 0.855 | 10,000 |
| Status in 2019 | 0.907 | 0.927 | 0.724 | 0.879 | 0.955 | 0.977 | 10,000 |
| Status in 2030 | 0.983 | 0.994 | 0.899 | 0.983 | 0.998 | 0.999 | 10,000 |

# 2.16) Scenario G-1

| Parameter | Mean | Median | 2.5% PI | 25% PI | 75% PI | 97.5% PI | Unique |
| --- | --- | --- | --- | --- | --- | --- | --- |
| *r_max_* | 0.091 | 0.093 | 0.054 | 0.08 | 0.105 | 0.117 | 10,000 |
| *K* | 28,204 | 27,739 | 25,329 | 26,685 | 29,256 | 33,693 | 10,000 |
| N_min_ | 476 | 375 | 194 | 282 | 556 | 1,313 | 10,000 |
| N_2006_ | 13,004 | 12,953 | 11,213 | 12,279 | 13,641 | 15,141 | 10,000 |
| N_2008_ | 15,015 | 14,976 | 13,306 | 14,365 | 15,610 | 16,940 | 10,000 |
| N_2012_ | 19,235 | 19,221 | 17,413 | 18,632 | 19,855 | 21,032 | 10,000 |
| N_2019_ | 24,880 | 24,913 | 23,183 | 24,375 | 25,429 | 26,333 | 10,000 |
| N_2030_ | 27,662 | 27,488 | 25,284 | 26,572 | 28,651 | 30,842 | 10,000 |
| Maximum depletion | 0.016 | 0.014 | 0.007 | 0.011 | 0.019 | 0.04 | 10,000 |
| Status in 2006 | 0.462 | 0.461 | 0.396 | 0.438 | 0.485 | 0.536 | 10,000 |
| Status in 2008 | 0.535 | 0.535 | 0.447 | 0.506 | 0.563 | 0.621 | 10,000 |
| Status in 2012 | 0.686 | 0.693 | 0.545 | 0.648 | 0.732 | 0.792 | 10,000 |
| Status in 2019 | 0.887 | 0.905 | 0.709 | 0.856 | 0.937 | 0.963 | 10,000 |
| Status in 2030 | 0.983 | 0.993 | 0.898 | 0.982 | 0.997 | 0.999 | 10,000 |

# 2.17) Scenario G-2

| Parameter | Mean | Median | 2.5% PI | 25% PI | 75% PI | 97.5% PI | Unique |
| --- | --- | --- | --- | --- | --- | --- | --- |
| *r_max_* | 0.091 | 0.094 | 0.054 | 0.08 | 0.105 | 0.116 | 10,000 |
| *K* | 28,221 | 27,742 | 25,327 | 26,703 | 29,258 | 33,735 | 10,000 |
| N_min_ | 476 | 372 | 197 | 284 | 560 | 1,308 | 10,000 |
| N_2006_ | 13,011 | 12,954 | 11,193 | 12,310 | 13,653 | 15,123 | 10,000 |
| N_2008_ | 15,023 | 14,998 | 13,294 | 14,386 | 15,620 | 16,927 | 10,000 |
| N_2012_ | 19,243 | 19,250 | 17,437 | 18,610 | 19,866 | 21,045 | 10,000 |
| N_2019_ | 24,888 | 24,916 | 23,133 | 24,384 | 25,438 | 26,372 | 10,000 |
| N_2030_ | 27,674 | 27,496 | 25,280 | 26,593 | 28,634 | 30,808 | 10,000 |
| Maximum depletion | 0.016 | 0.013 | 0.008 | 0.011 | 0.019 | 0.04 | 10,000 |
| Status in 2006 | 0.462 | 0.462 | 0.396 | 0.438 | 0.486 | 0.536 | 10,000 |
| Status in 2008 | 0.535 | 0.535 | 0.446 | 0.506 | 0.565 | 0.62 | 10,000 |
| Status in 2012 | 0.686 | 0.693 | 0.543 | 0.647 | 0.733 | 0.792 | 10,000 |
| Status in 2019 | 0.887 | 0.905 | 0.71 | 0.856 | 0.936 | 0.963 | 10,000 |
| Status in 2030 | 0.983 | 0.993 | 0.9 | 0.982 | 0.997 | 0.999 | 10,000 |

# 2.18) Scenario M-1

| Parameter | Mean | Median | 2.5% PI | 25% PI | 75% PI | 97.5% PI | Unique |
| --- | --- | --- | --- | --- | --- | --- | --- |
| *r_max_* | 0.083 | 0.083 | 0.049 | 0.071 | 0.096 | 0.114 | 10,000 |
| *K* | 26,032 | 25,799 | 23,277 | 24,669 | 27,076 | 30,391 | 10,000 |
| N_min_ | 576 | 473 | 187 | 331 | 714 | 1,466 | 10,000 |
| N_2006_ | 12,720 | 12,653 | 10,815 | 11,988 | 13,413 | 14,946 | 10,000 |
| N_2008_ | 14,818 | 14,781 | 13,088 | 14,170 | 15,425 | 16,792 | 10,000 |
| N_2012_ | 19,586 | 19,607 | 17,605 | 18,918 | 20,258 | 21,465 | 10,000 |
| N_2019_ | 24,909 | 24,958 | 23,169 | 24,326 | 25,533 | 26,516 | 10,000 |
| N_2030_ | 25,970 | 25,795 | 23,277 | 24,669 | 27,055 | 29,796 | 10,000 |
| Maximum depletion | 0.021 | 0.018 | 0.008 | 0.013 | 0.026 | 0.049 | 10,000 |
| Status in 2006 | 0.489 | 0.488 | 0.431 | 0.468 | 0.509 | 0.554 | 10,000 |
| Status in 2008 | 0.571 | 0.571 | 0.491 | 0.543 | 0.598 | 0.653 | 10,000 |
| Status in 2012 | 0.757 | 0.76 | 0.607 | 0.708 | 0.811 | 0.884 | 10,000 |
| Status in 2019 | 0.96 | 0.979 | 0.814 | 0.946 | 0.994 | 0.999 | 10,000 |
| Status in 2030 | 0.998 | 1 | 0.982 | 0.999 | 1 | 1 | 10,000 |

# 2.19) Scenario M-2

| Parameter | Mean | Median | 2.5% PI | 25% PI | 75% PI | 97.5% PI | Unique |
| --- | --- | --- | --- | --- | --- | --- | --- |
| *r_max_* | 0.076 | 0.076 | 0.047 | 0.065 | 0.087 | 0.108 | 9,999 |
| *K* | 24,682 | 24,521 | 21,933 | 23,540 | 25,653 | 28,267 | 10,000 |
| N_min_ | 690 | 593 | 225 | 419 | 875 | 1,574 | 10,000 |
| N_2006_ | 12,786 | 12,749 | 10,802 | 12,035 | 13,504 | 14,956 | 10,000 |
| N_2008_ | 14,778 | 14,751 | 13,015 | 14,128 | 15,401 | 16,693 | 10,000 |
| N_2012_ | 19,652 | 19,657 | 17,685 | 18,983 | 20,341 | 21,537 | 10,000 |
| N_2019_ | 24,387 | 24,460 | 21,927 | 23,516 | 25,319 | 26,485 | 10,000 |
| N_2030_ | 24,677 | 24,521 | 21,933 | 23,540 | 25,653 | 28,256 | 10,000 |
| Maximum depletion | 0.027 | 0.024 | 0.01 | 0.018 | 0.034 | 0.056 | 10,000 |
| Status in 2006 | 0.518 | 0.517 | 0.465 | 0.499 | 0.537 | 0.576 | 10,000 |
| Status in 2008 | 0.6 | 0.6 | 0.529 | 0.576 | 0.624 | 0.671 | 10,000 |
| Status in 2012 | 0.8 | 0.801 | 0.653 | 0.75 | 0.852 | 0.939 | 10,000 |
| Status in 2019 | 0.989 | 1 | 0.896 | 0.996 | 1 | 1 | 9,993 |
| Status in 2030 | 1 | 1 | 1 | 1 | 1 | 1 | 5,659 |

**3) Population Trajectories**

**3.1) Reference Case**

**
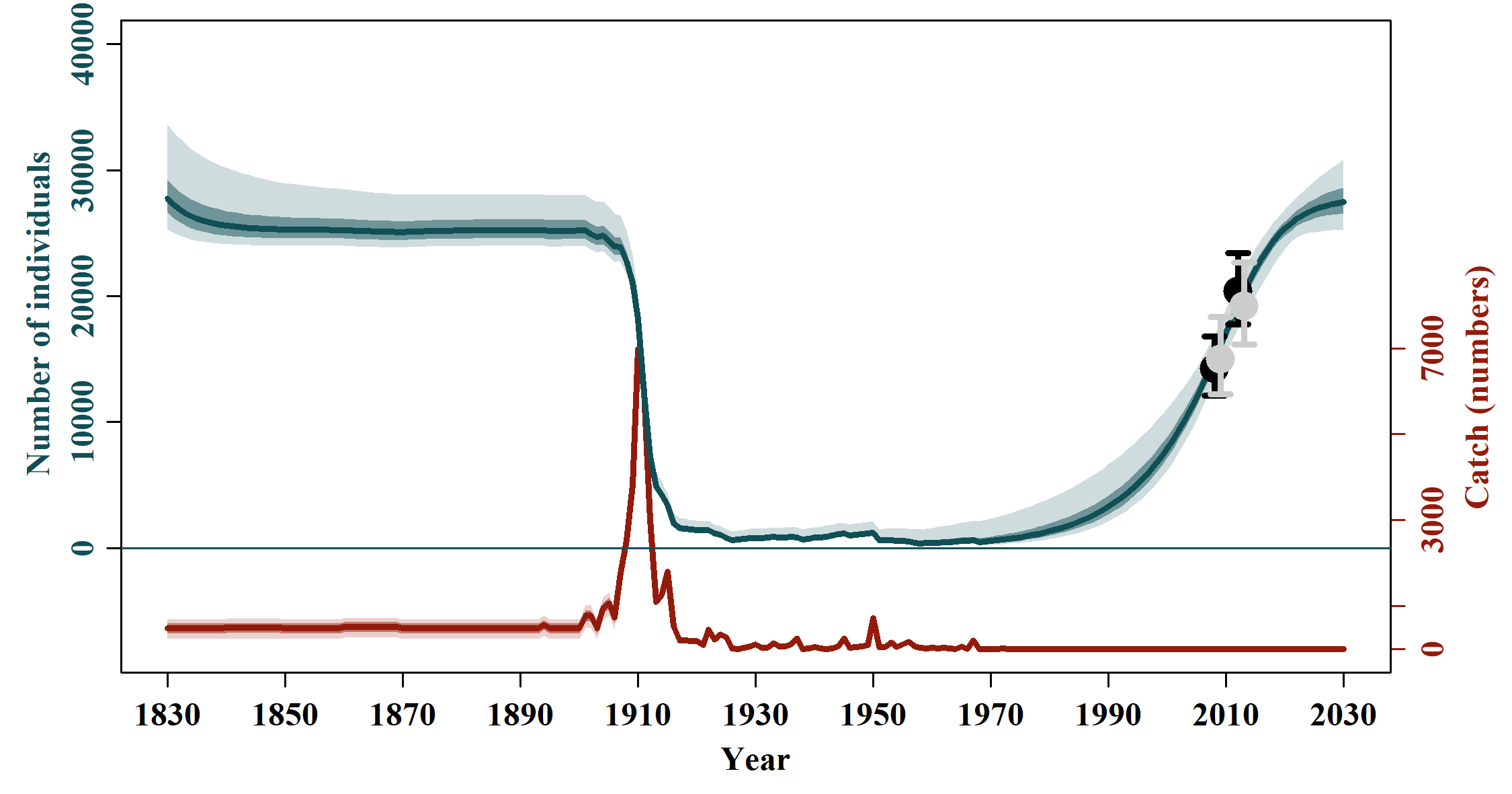
**

**3.2) Sensitivities to Data Inclusion (Scenarios D)**

| **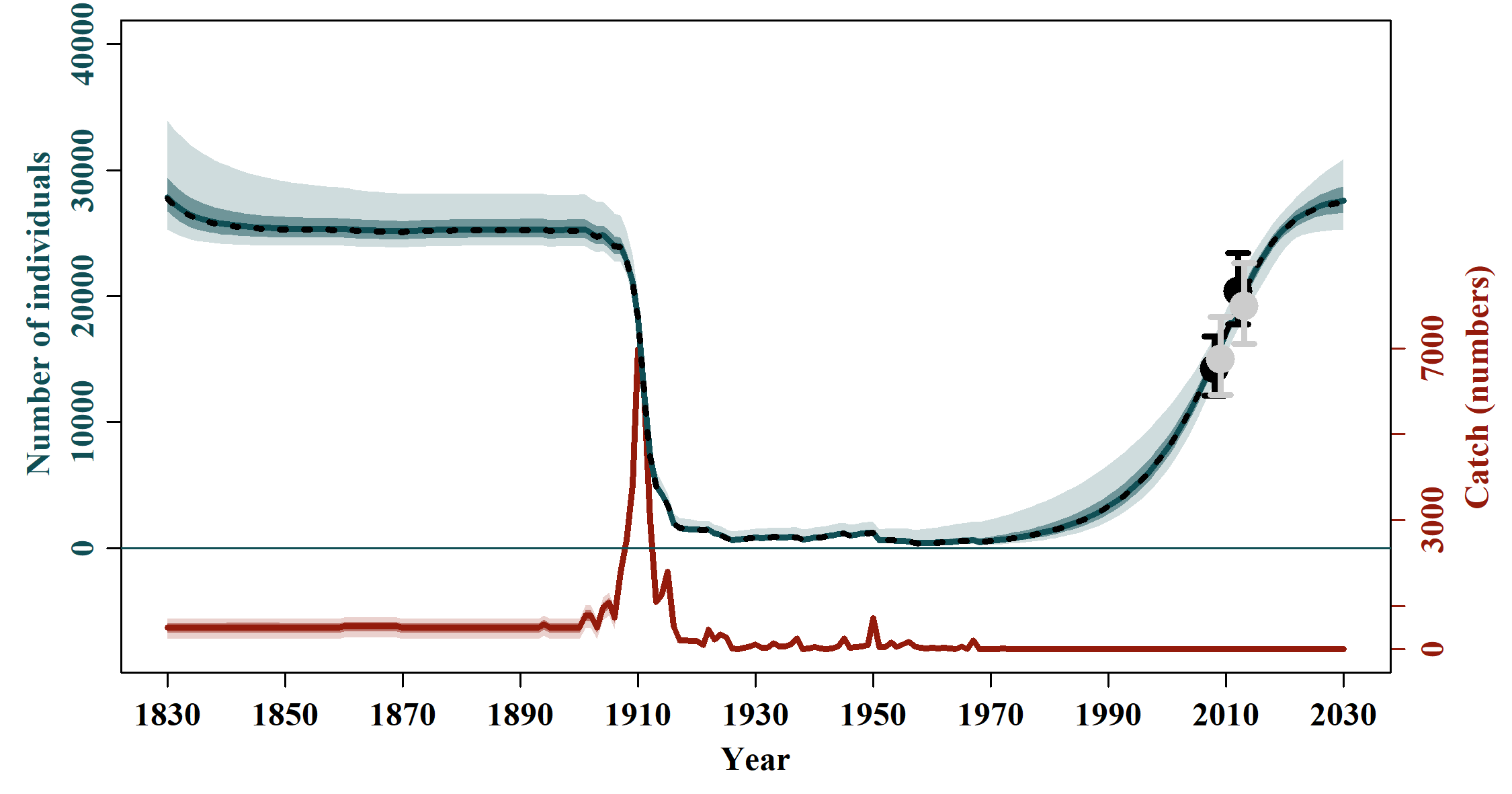** | **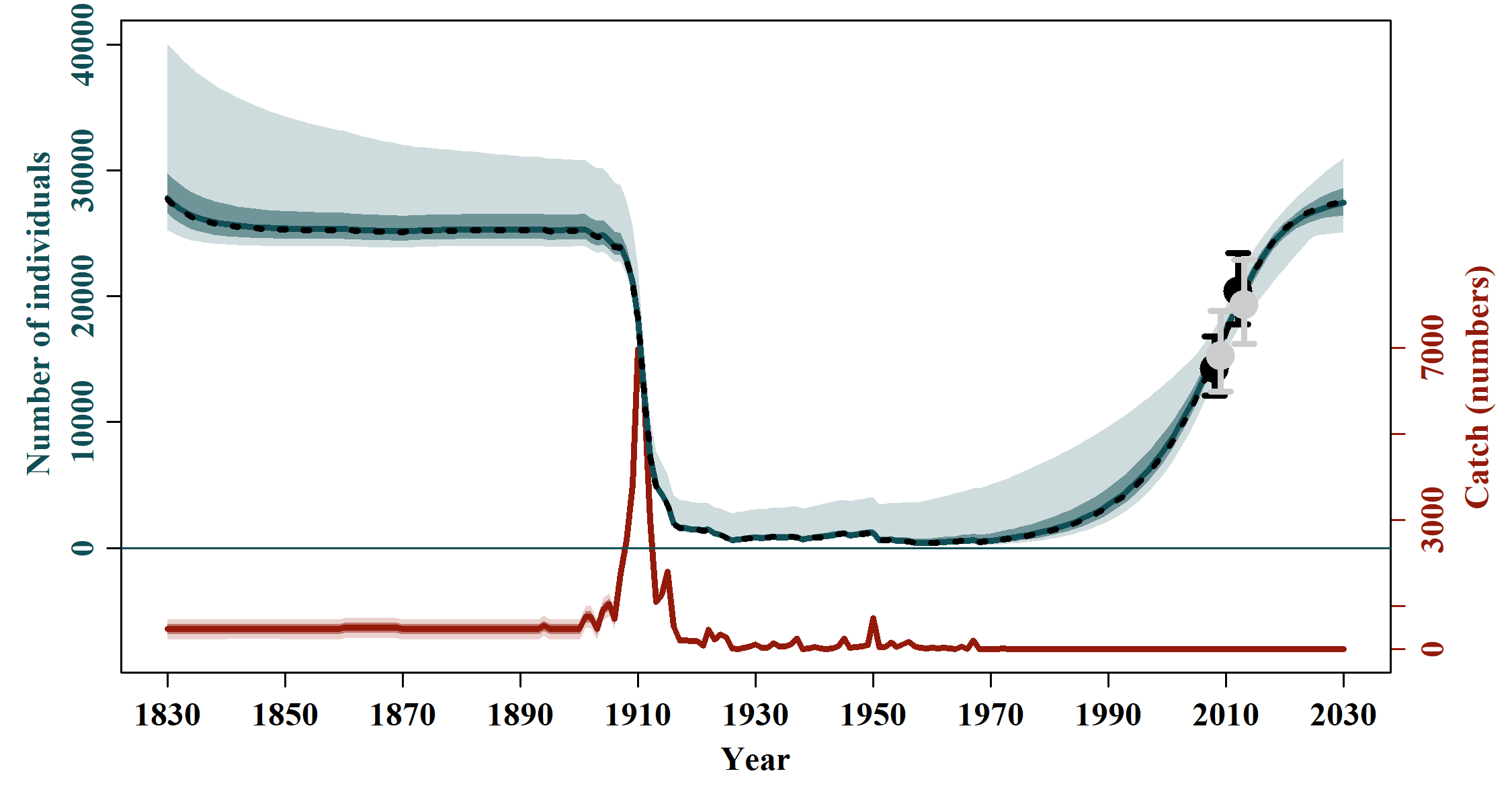** |
| --- | --- |
| D-1 | D-2 |
|  |  |
| 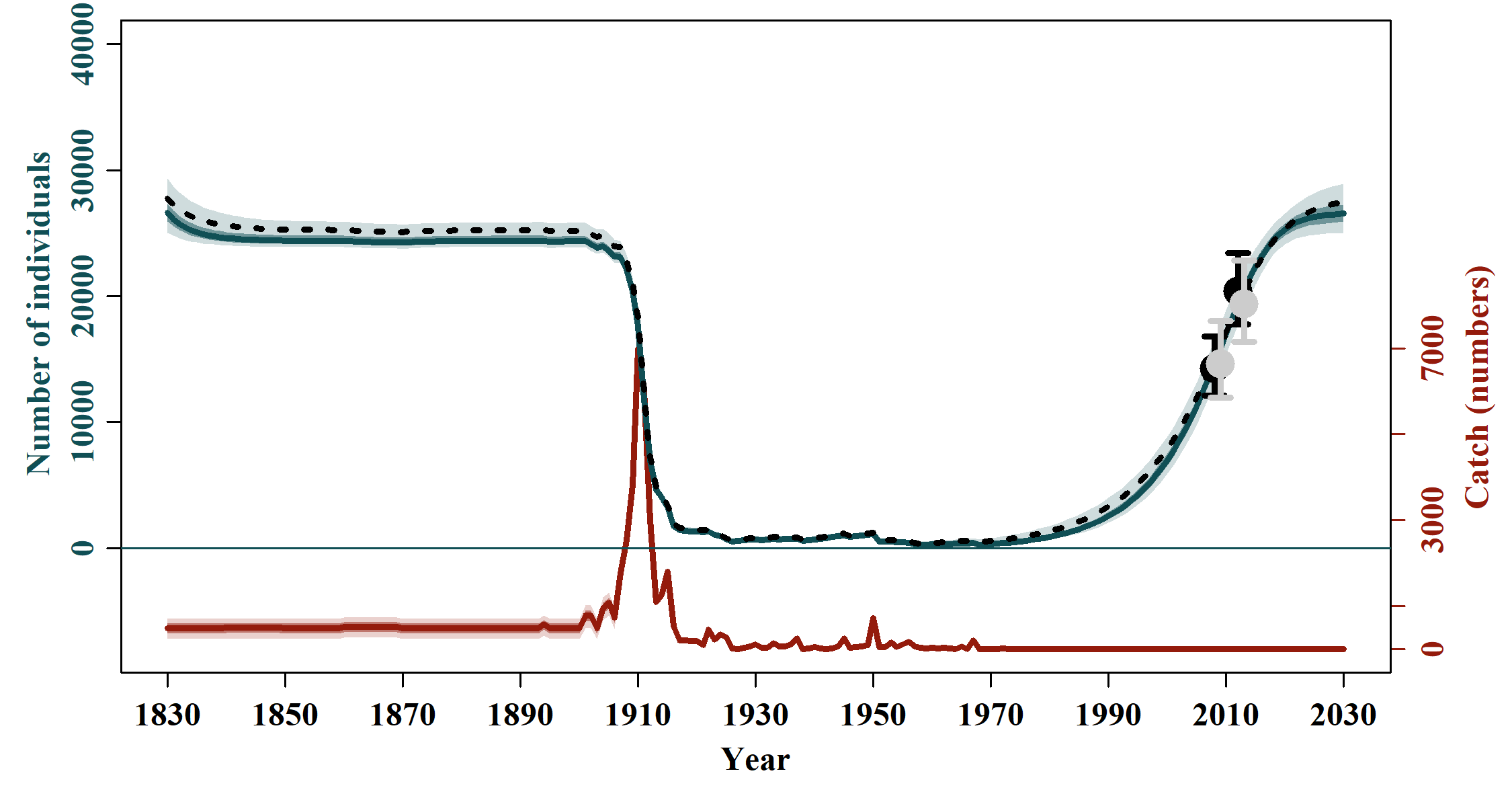 | 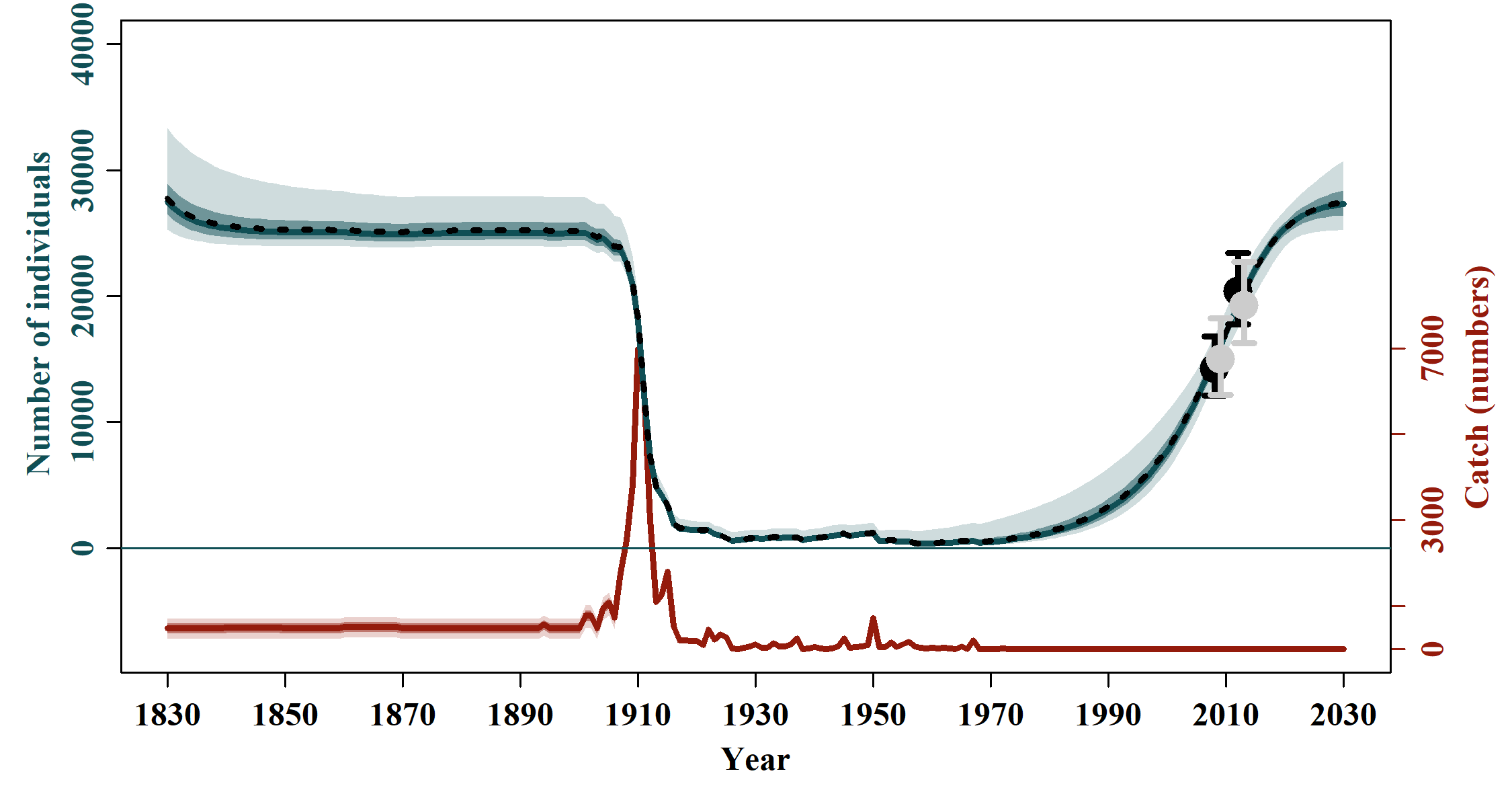 |
| D-3 | D-4 |
|  |  |
| 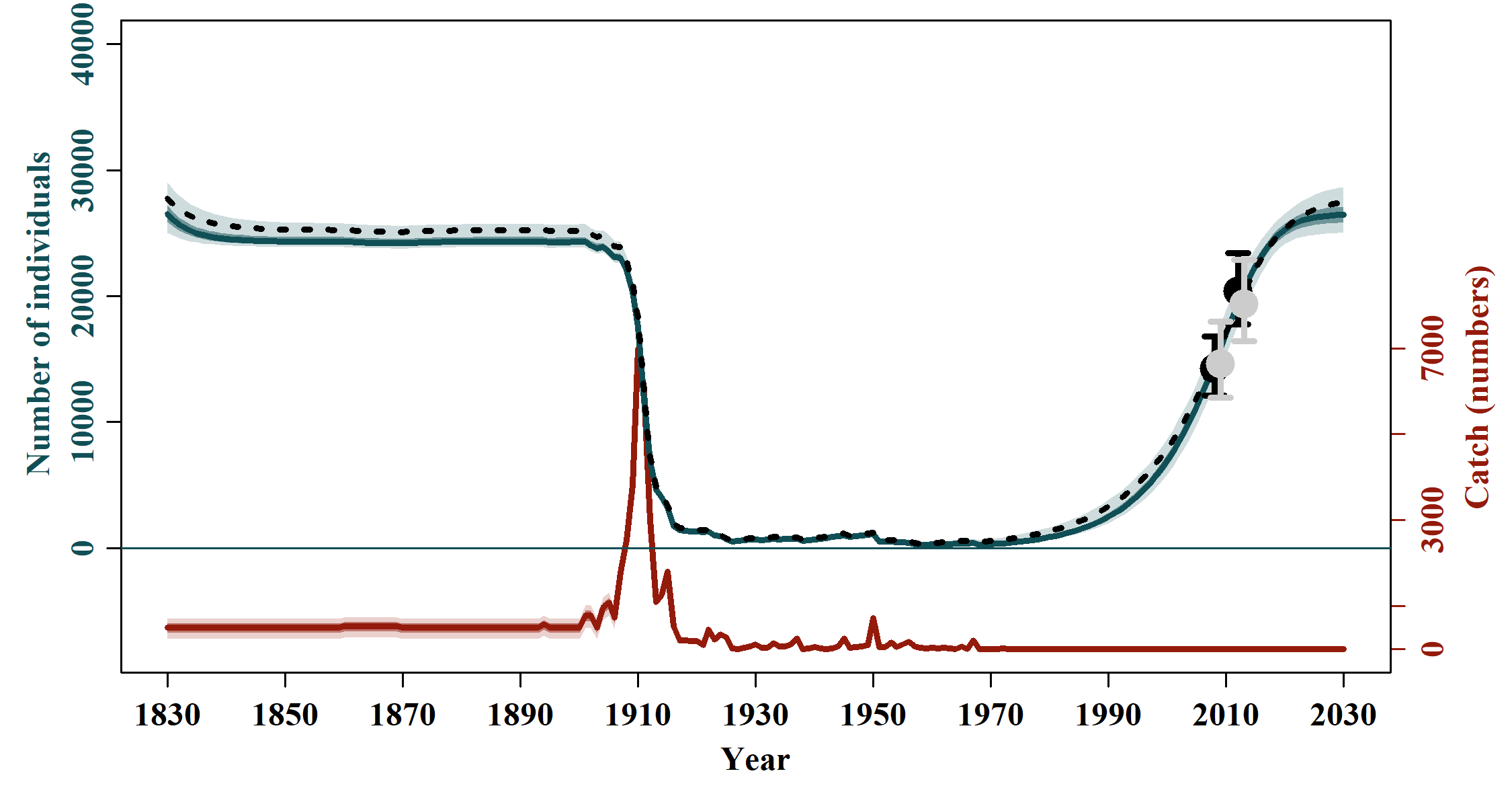 | 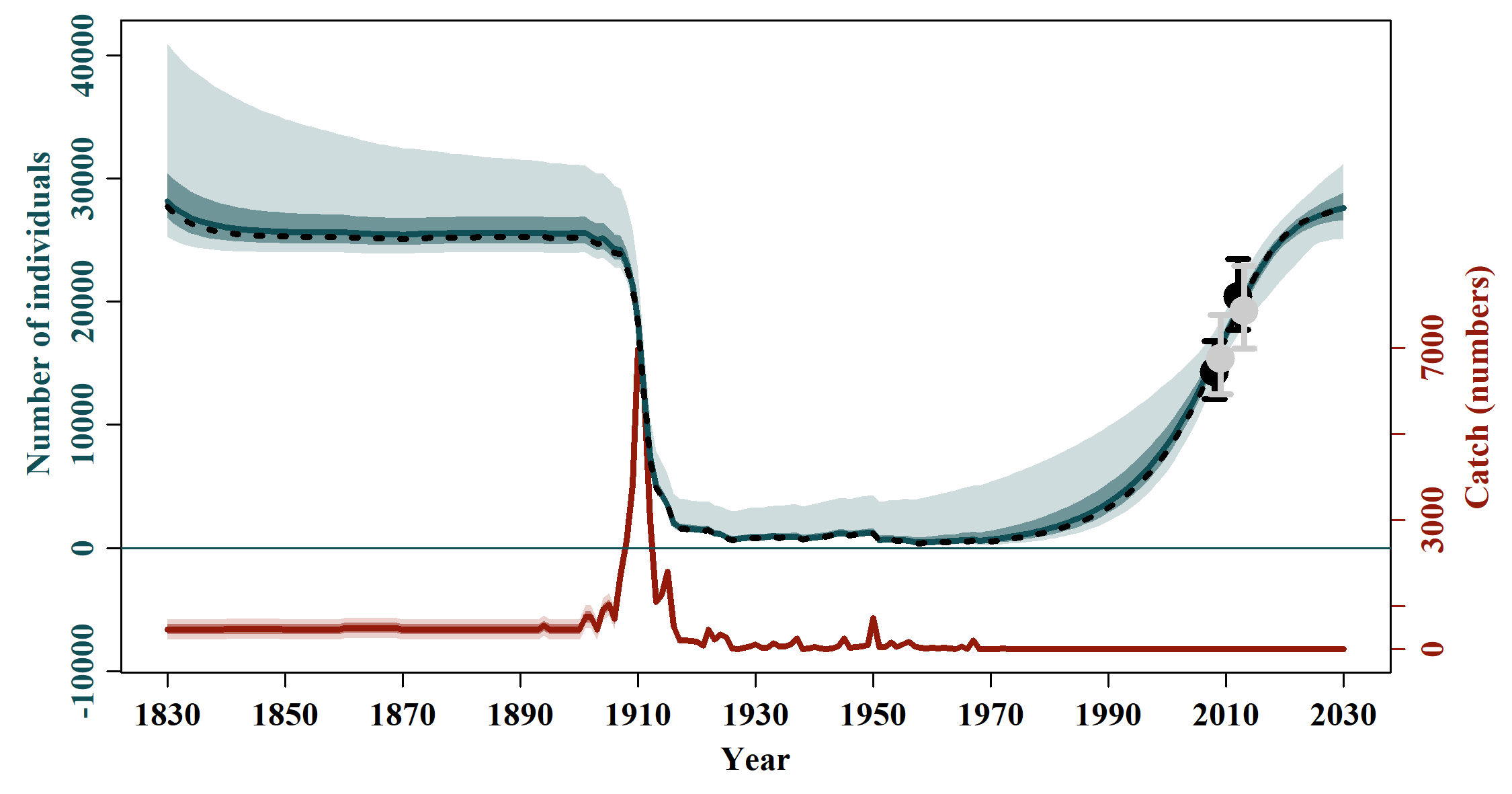 |
| D-5 | D-6 |
| 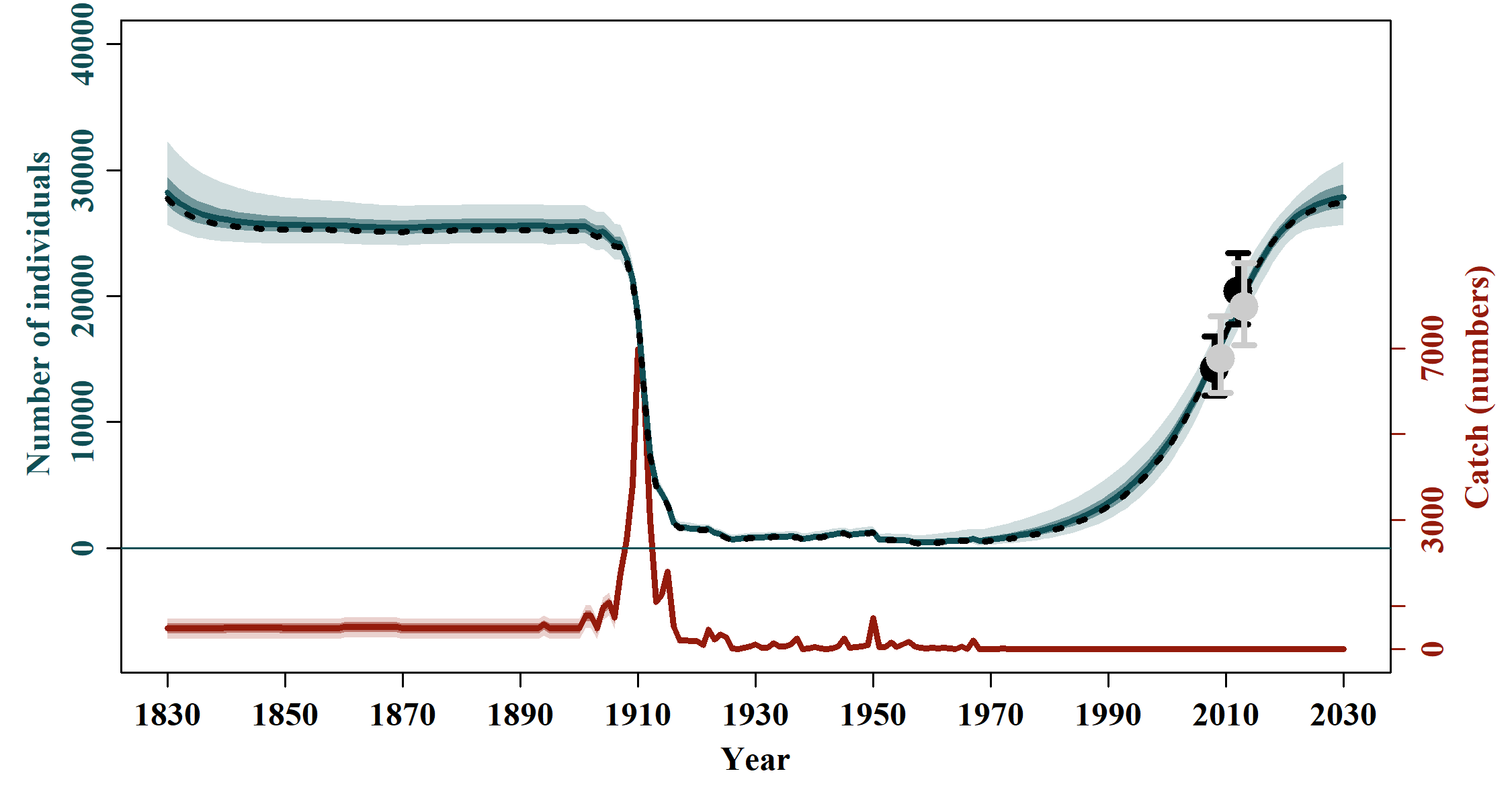  D-7 |  |

**3.3) Sensitivities to Catch Allocation and Struck and Lost Rates (Scenarios C)**

| 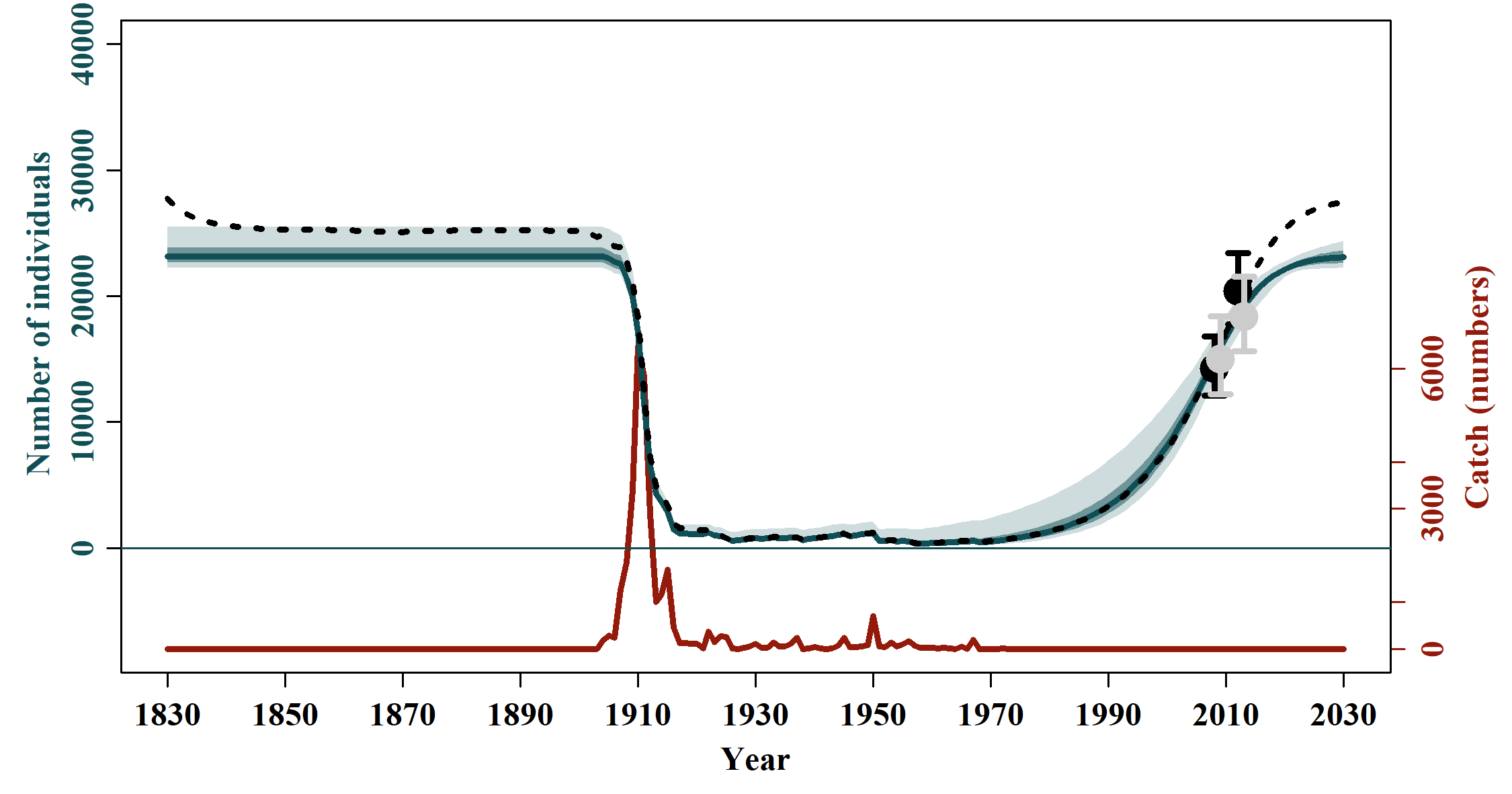 | 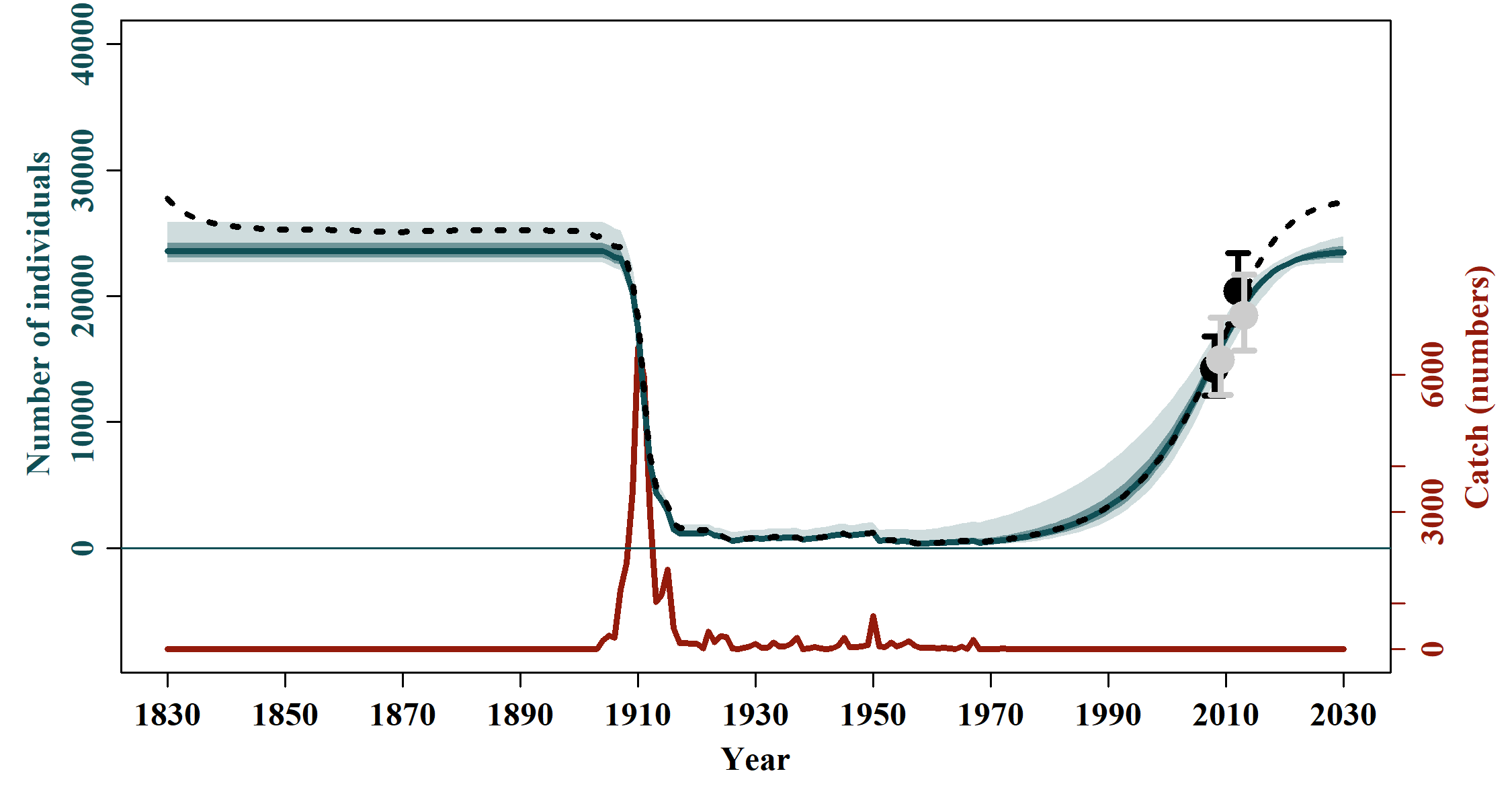 |
| --- | --- |
| C-1 | C-2 |
|  |  |
| 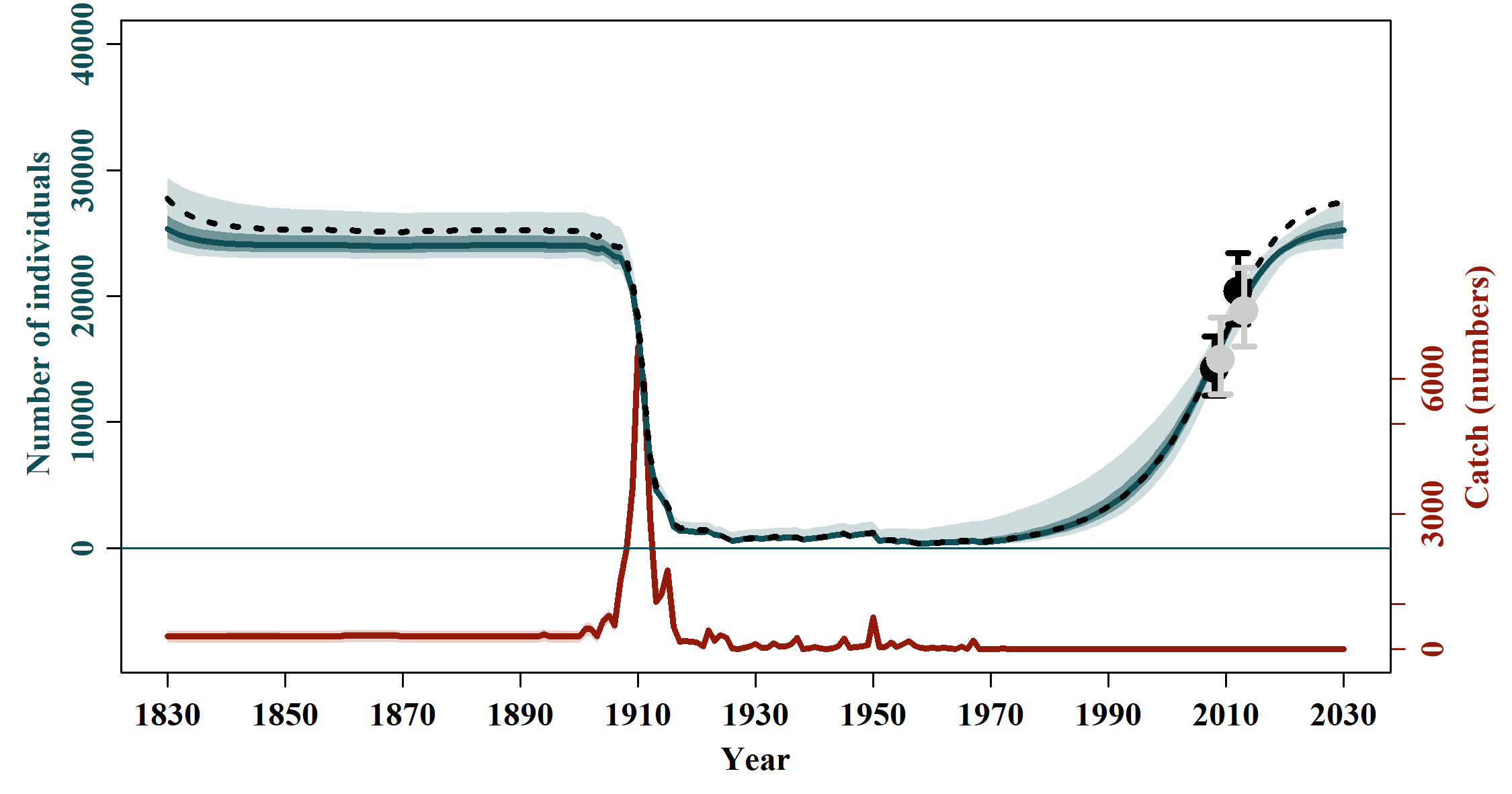 | 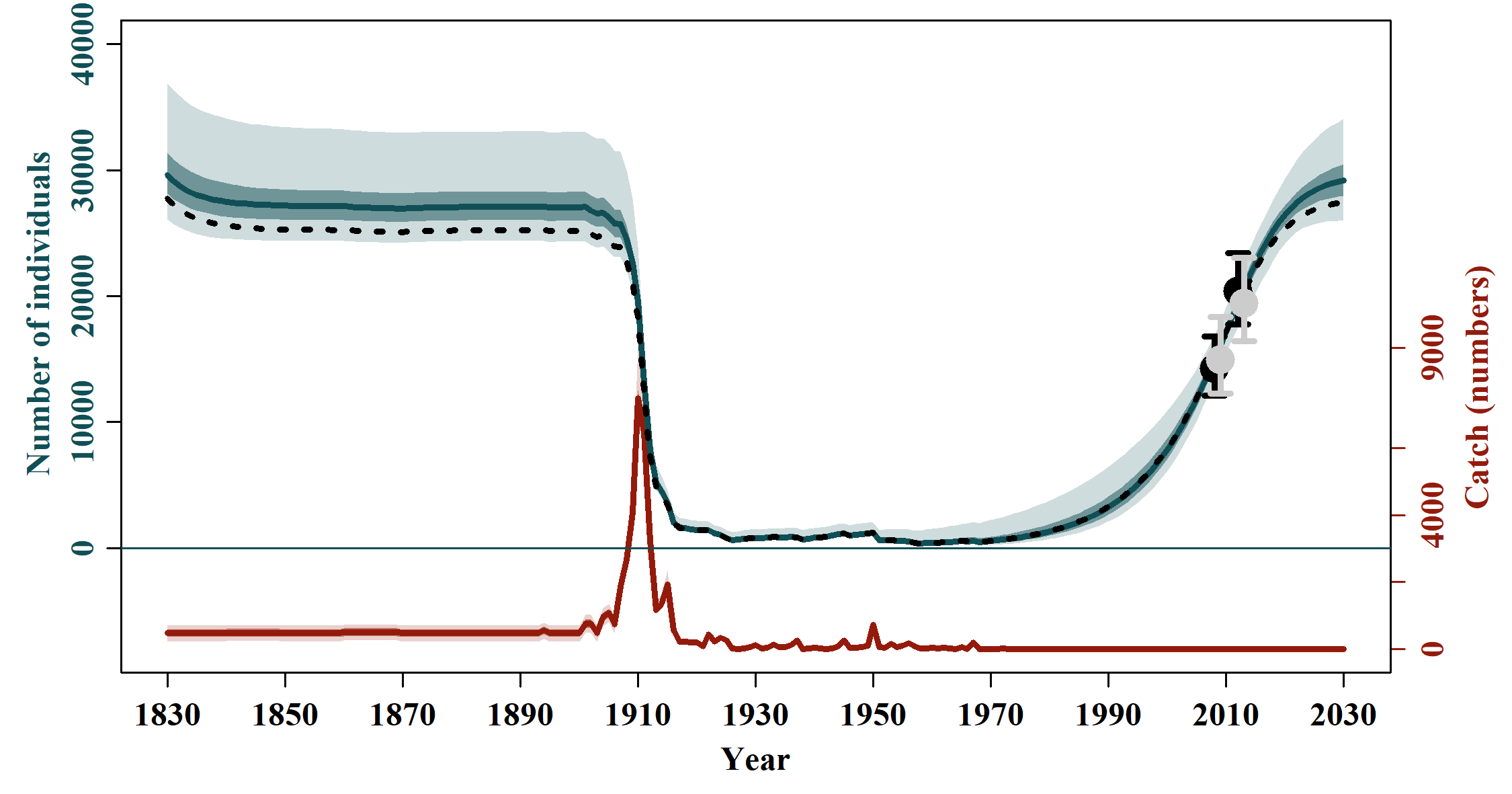 |
| C-3 | C-4 |
|  |  |
| 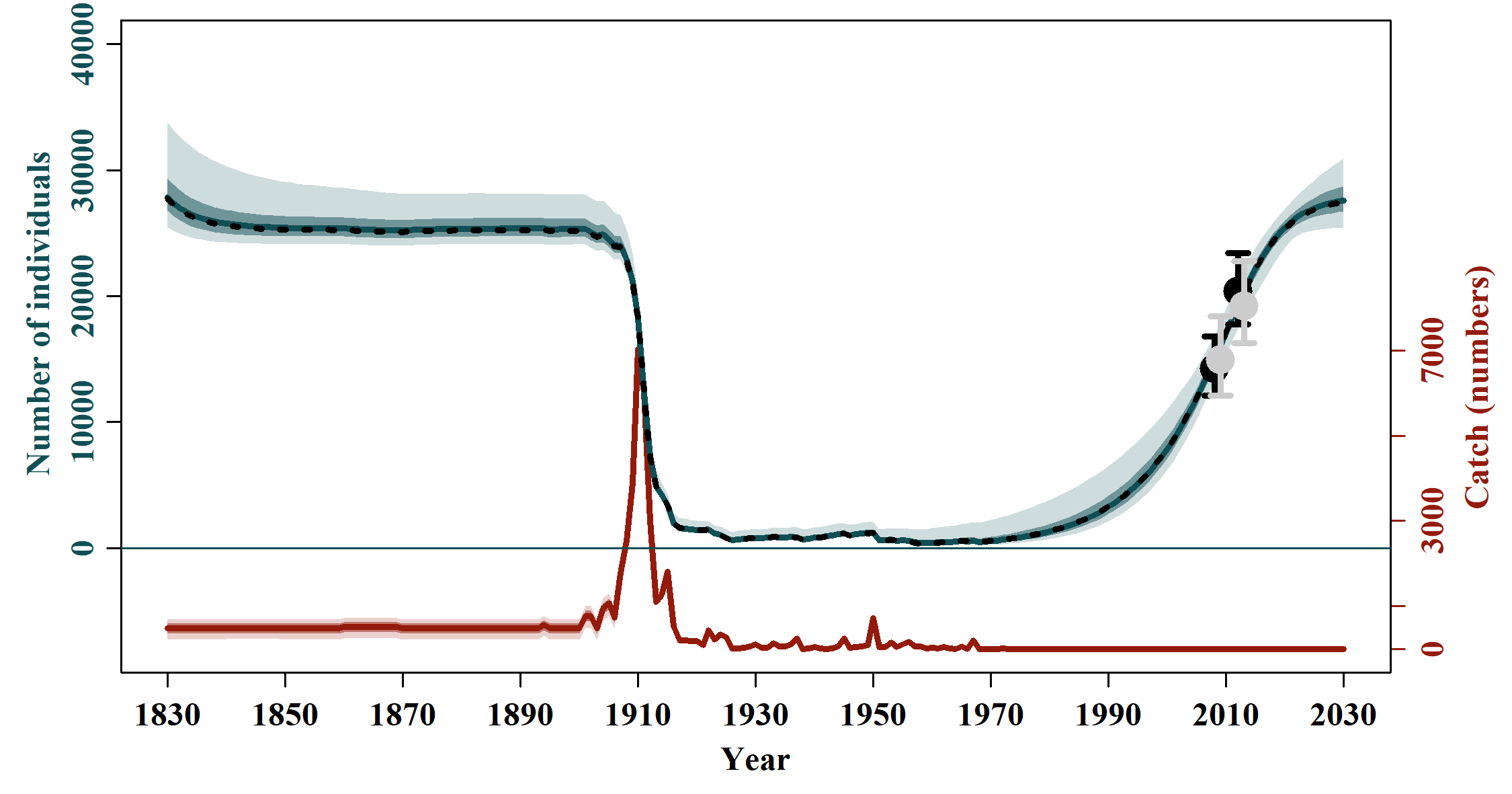 | 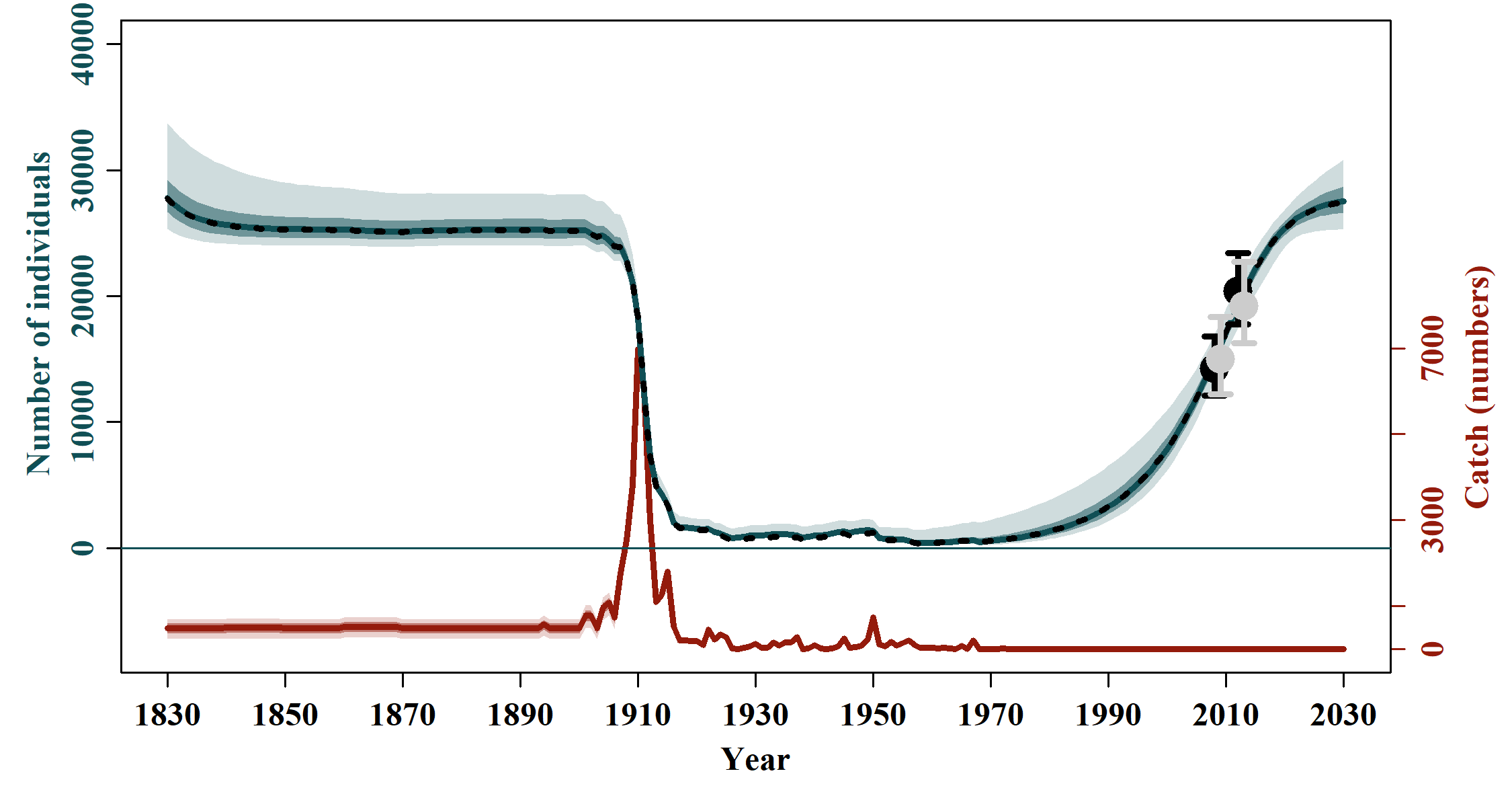 |
| C-5 | C-6 |
|  |  |
| 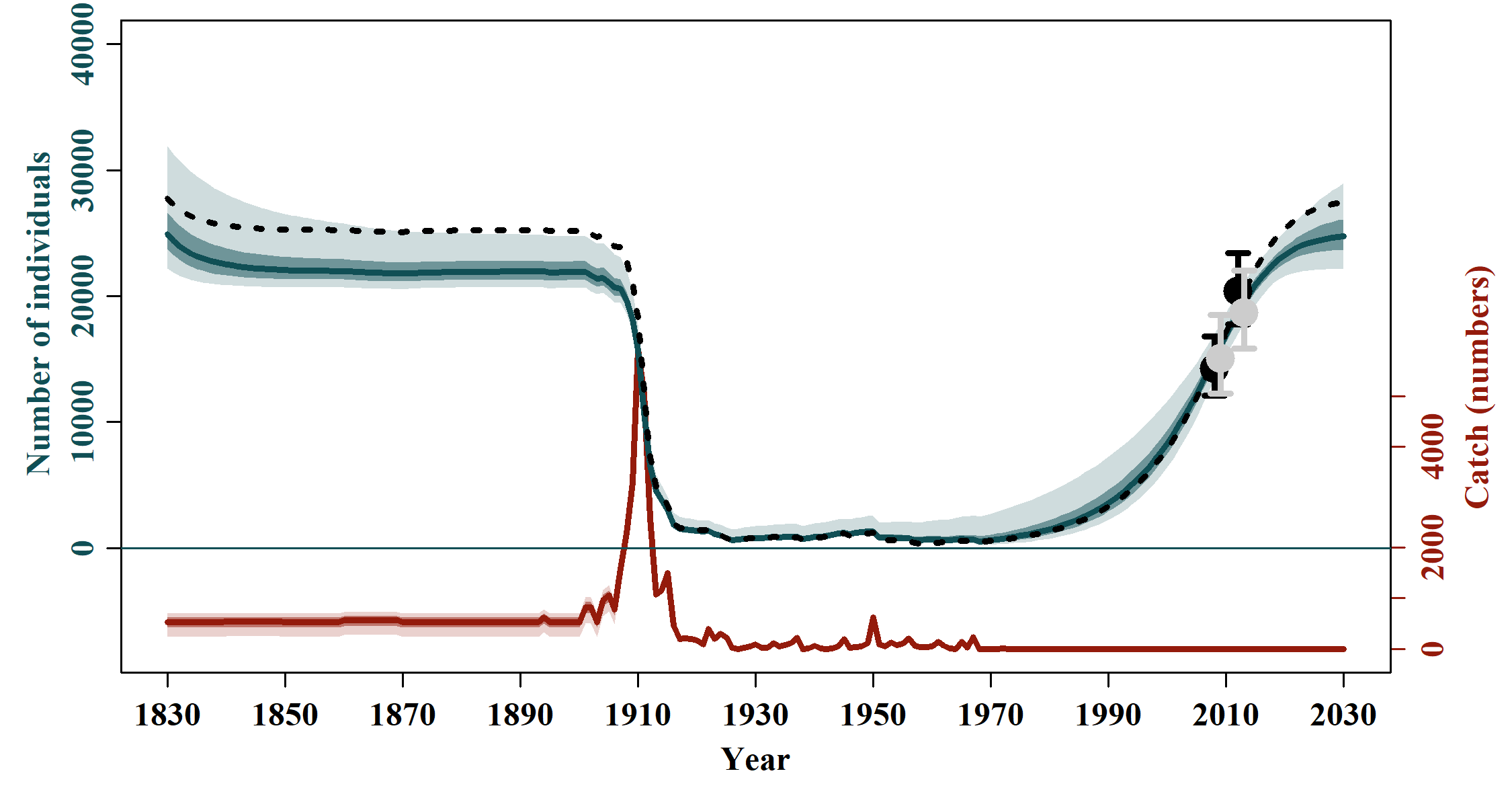 |  |
| C-7 |  |

**3.4) Sensitivities to Nmin constraints (Scenarios G) and to maximum productivity level (Scenarios M)**

| 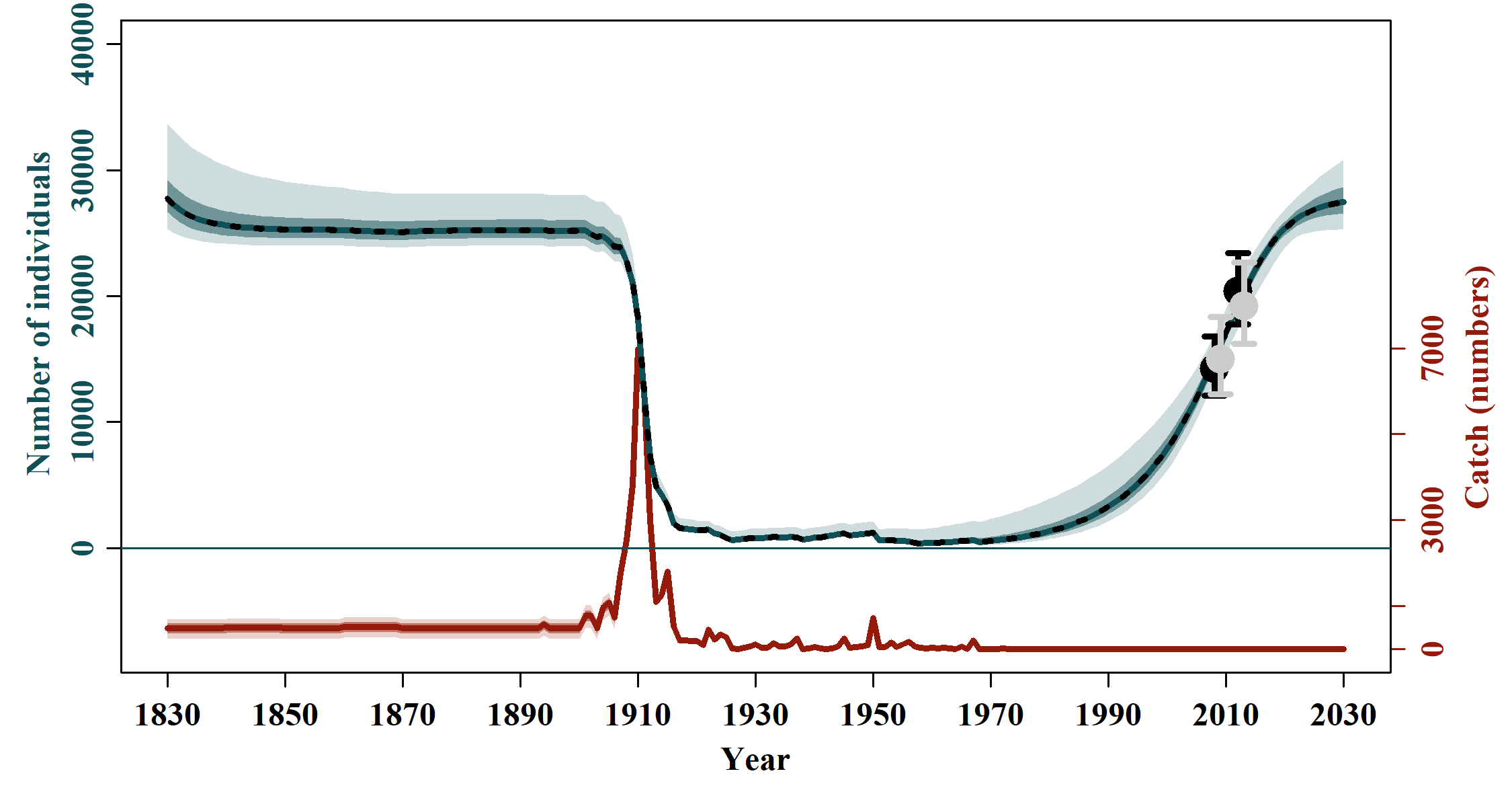 | 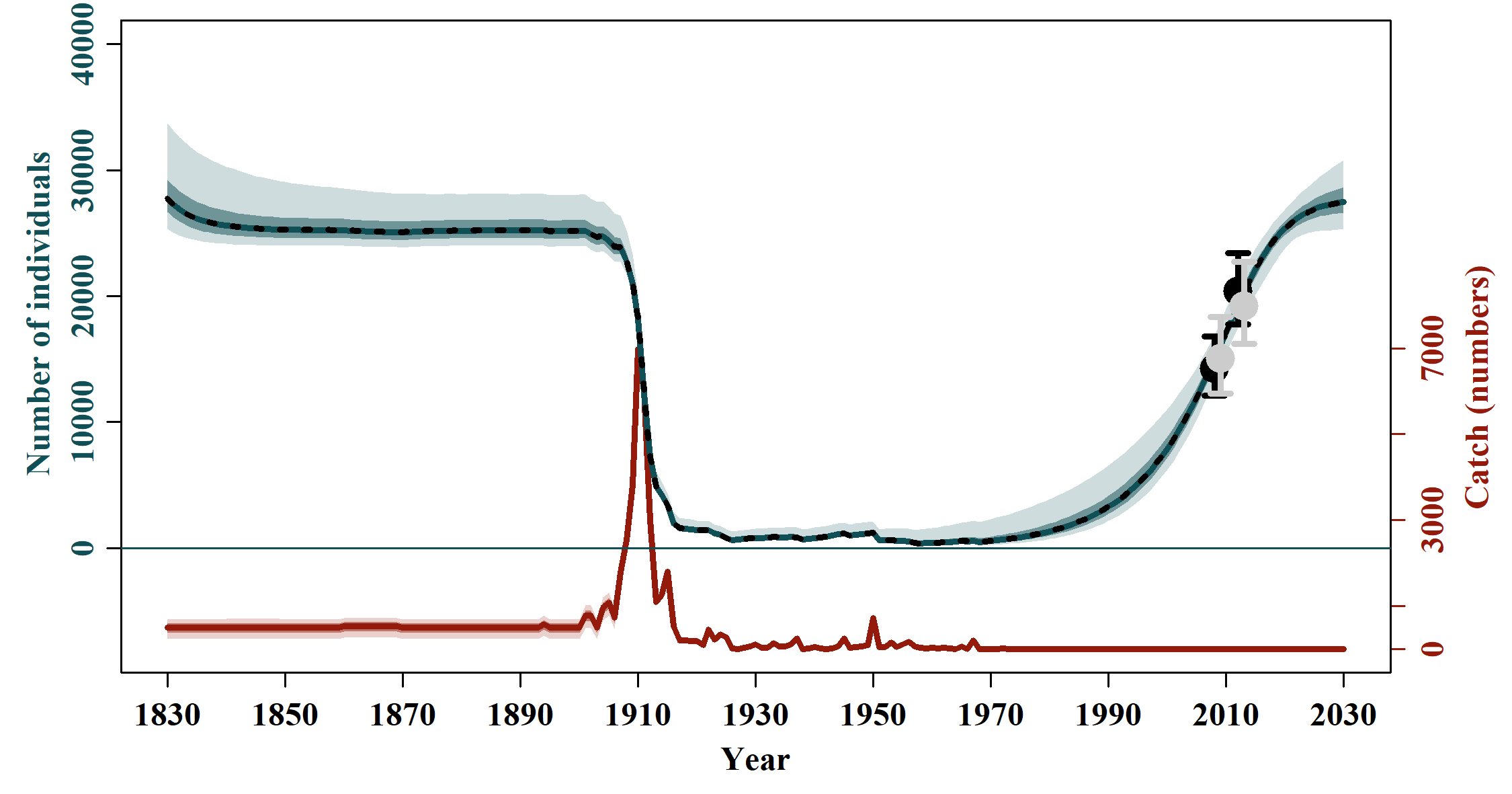 |
| --- | --- |
| G-1 | G-2 |
|  |  |
| 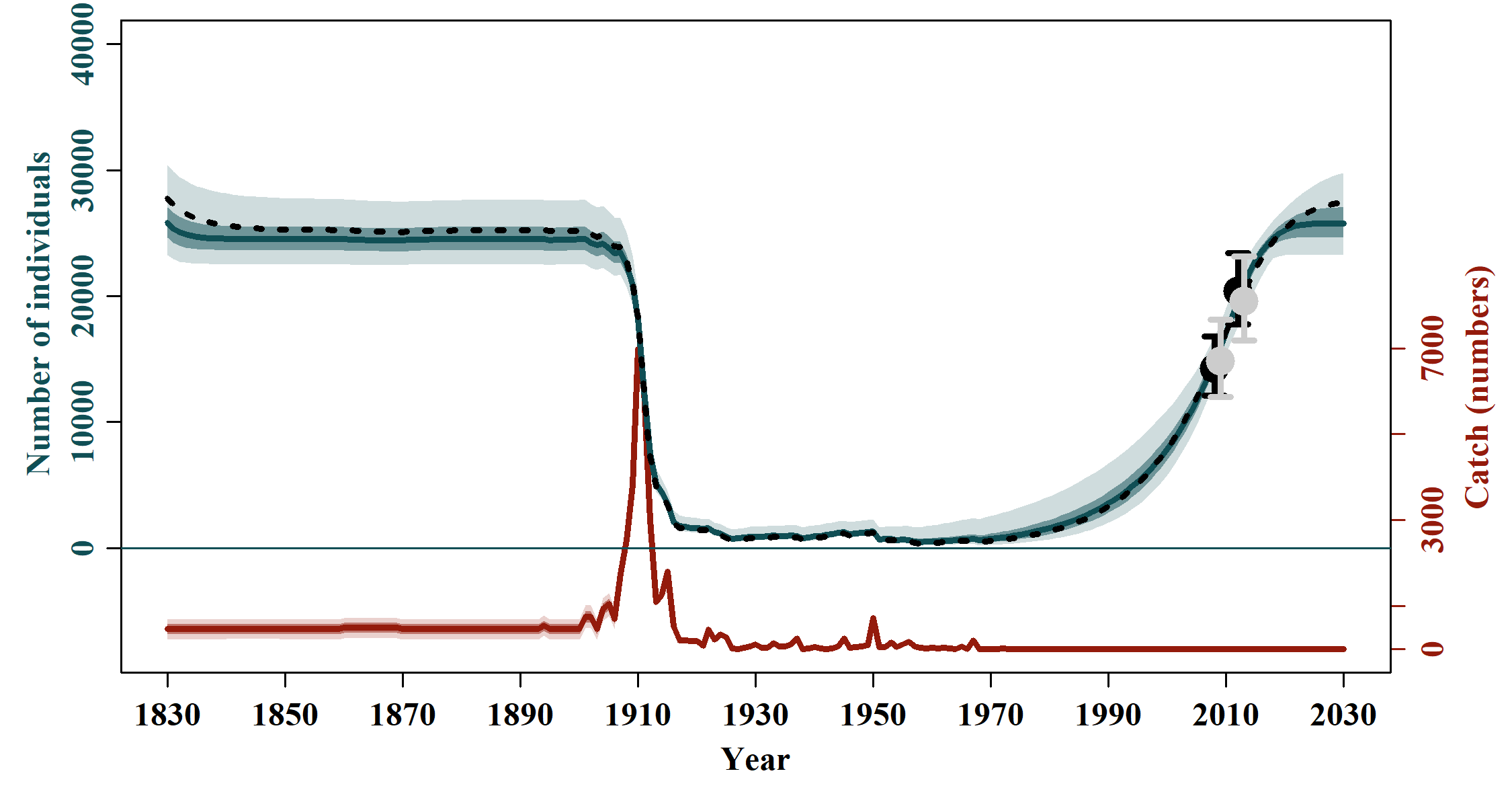 | 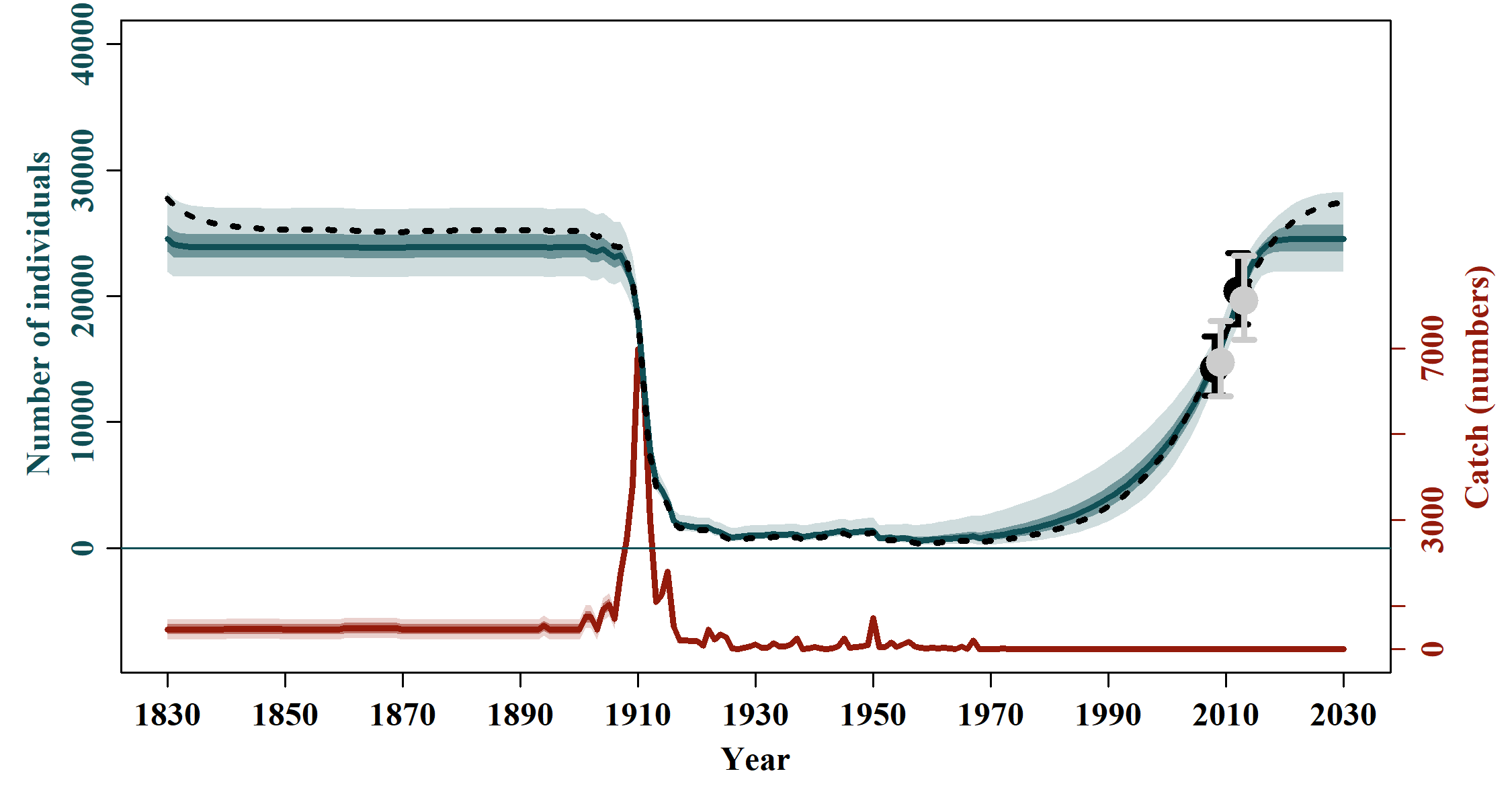 |
| M-1 | M-2 |

**4) Model fit to the Indices of Abundance (FG = feeding grounds, BG = breeding grounds)**

**4.1) Model Average**

**
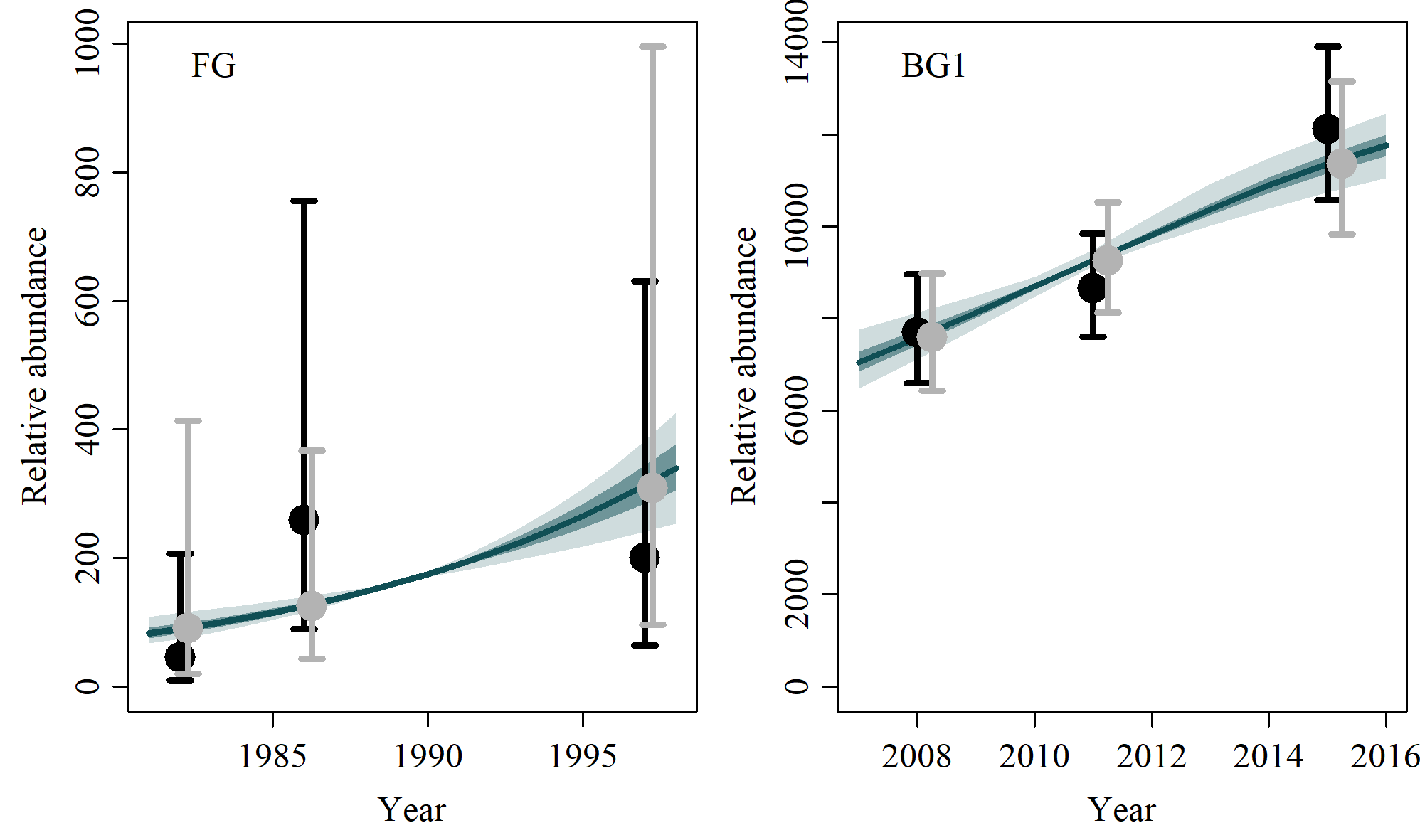
**

**4.2) Reference Case**

**
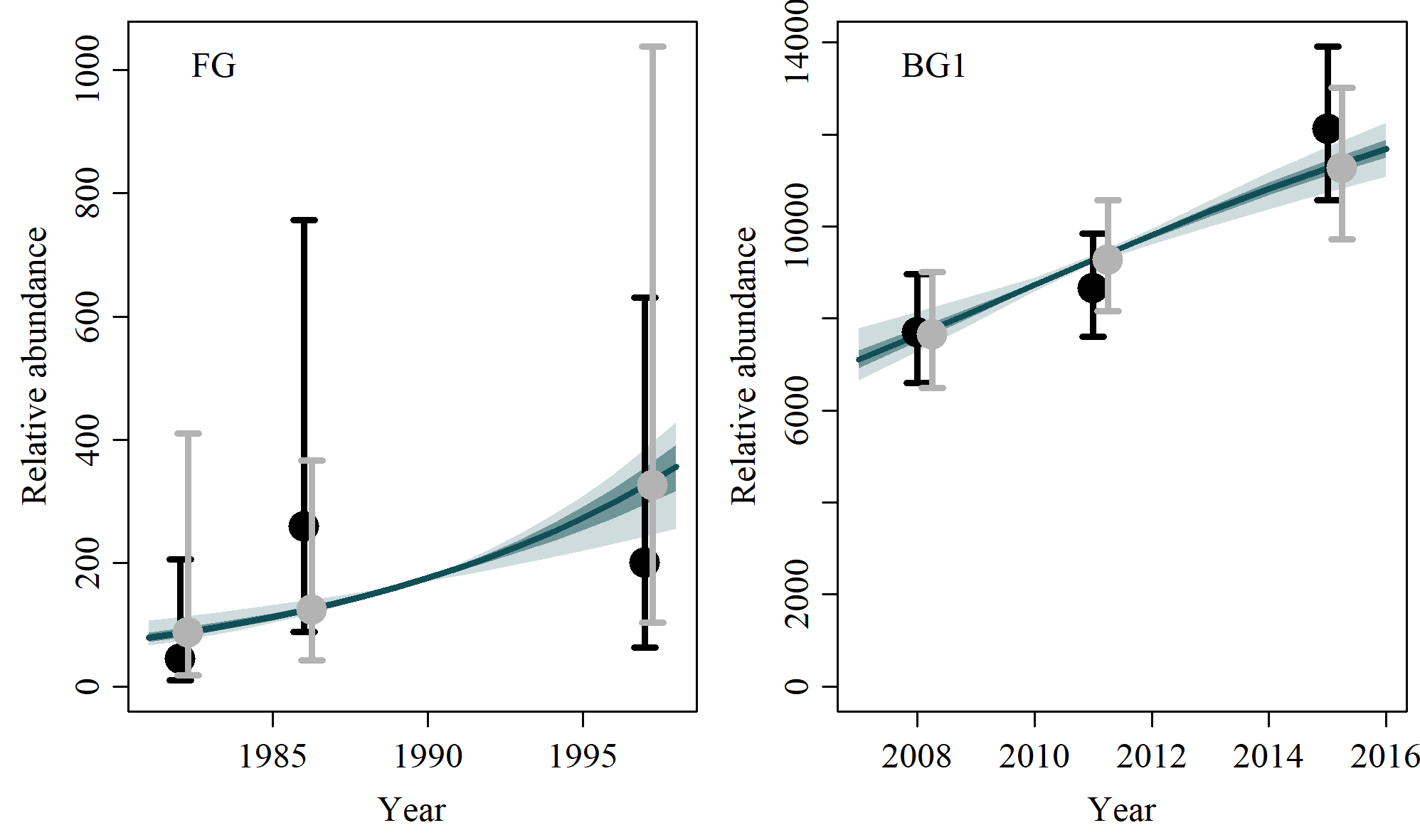
**

**4.3) Scenario D-1**

**
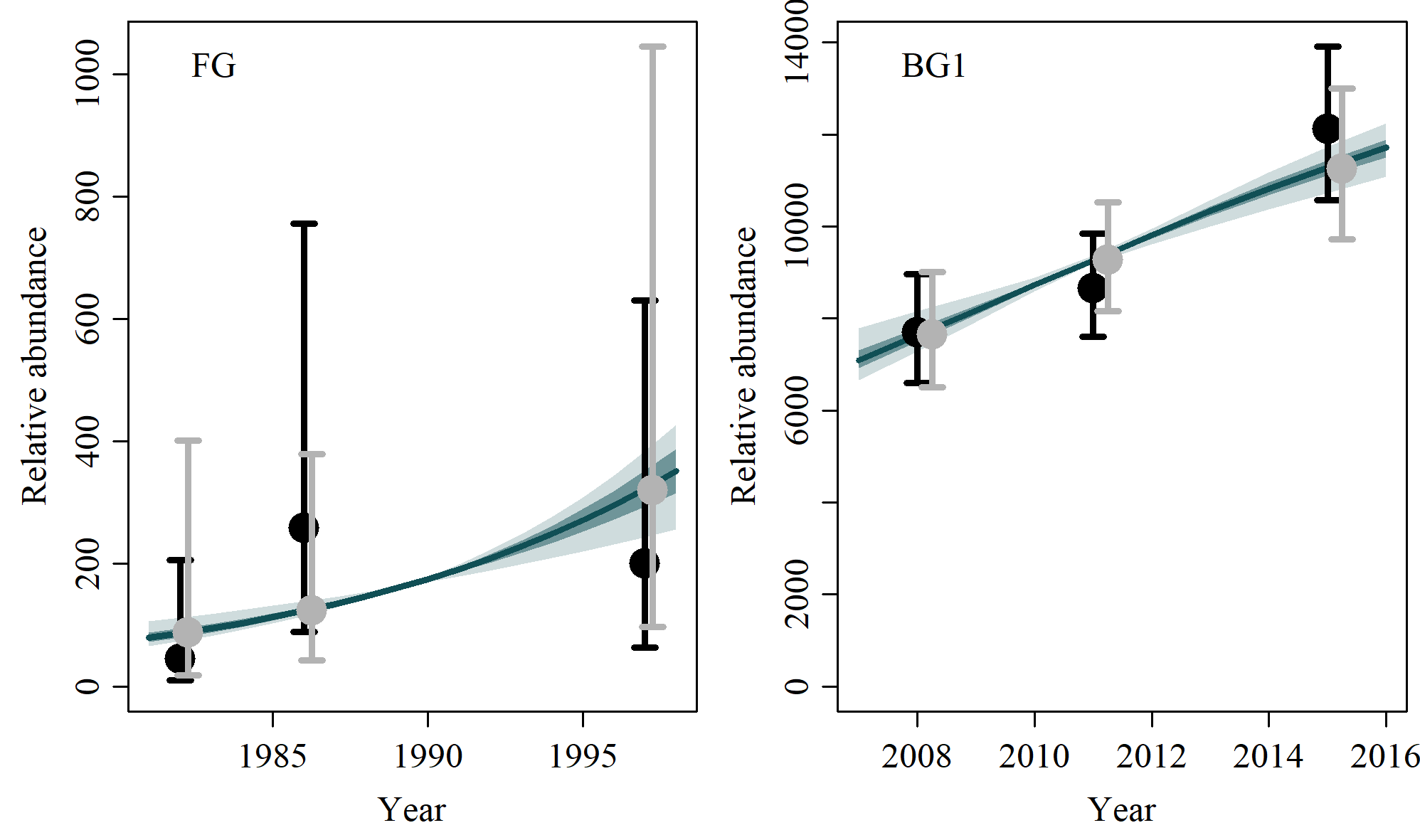
**

**4.4) Scenario D-2**

No output because no index of abundance was used.

**4.5) Scenario D-3**

**
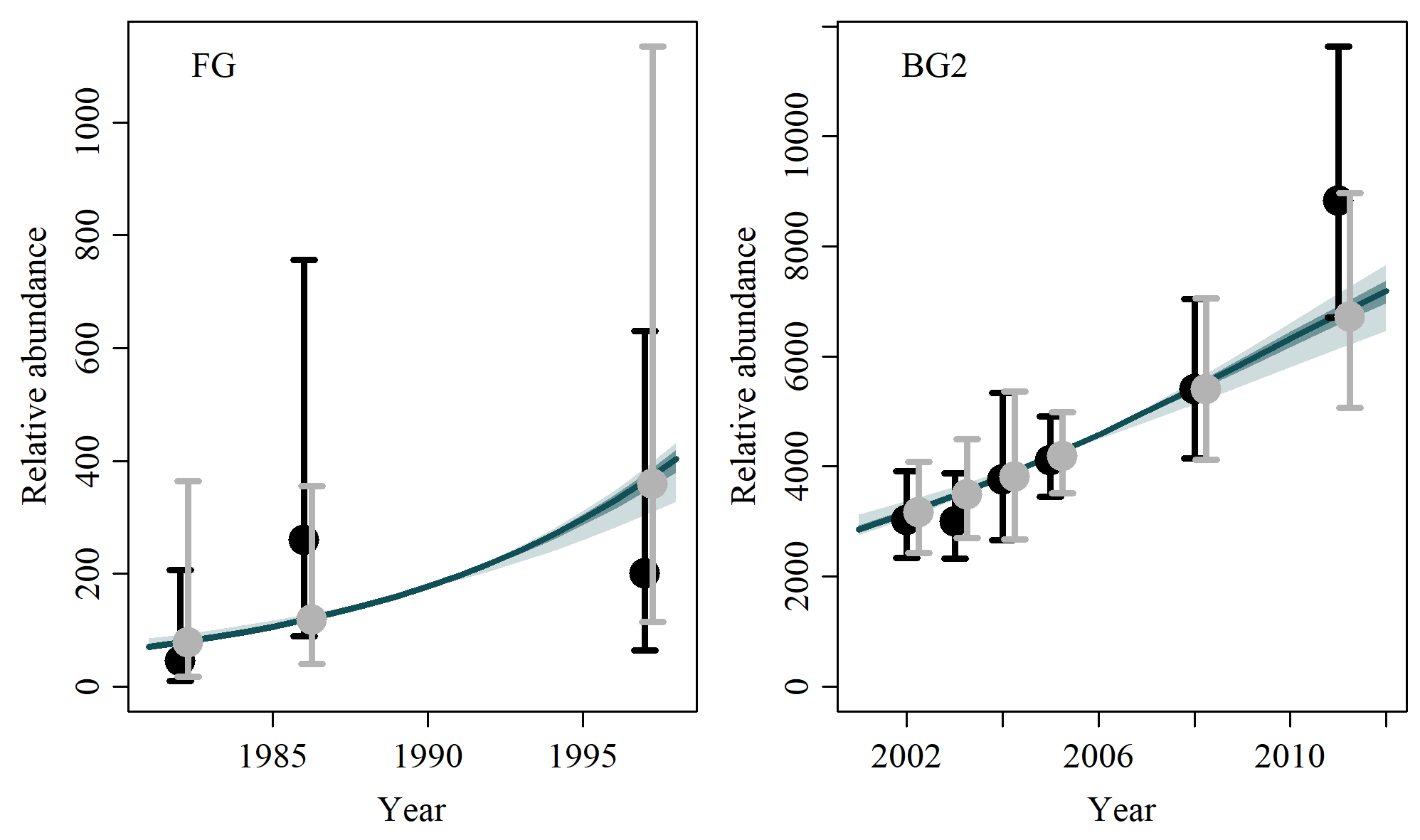
**

**4.6) Scenario D-4**

**
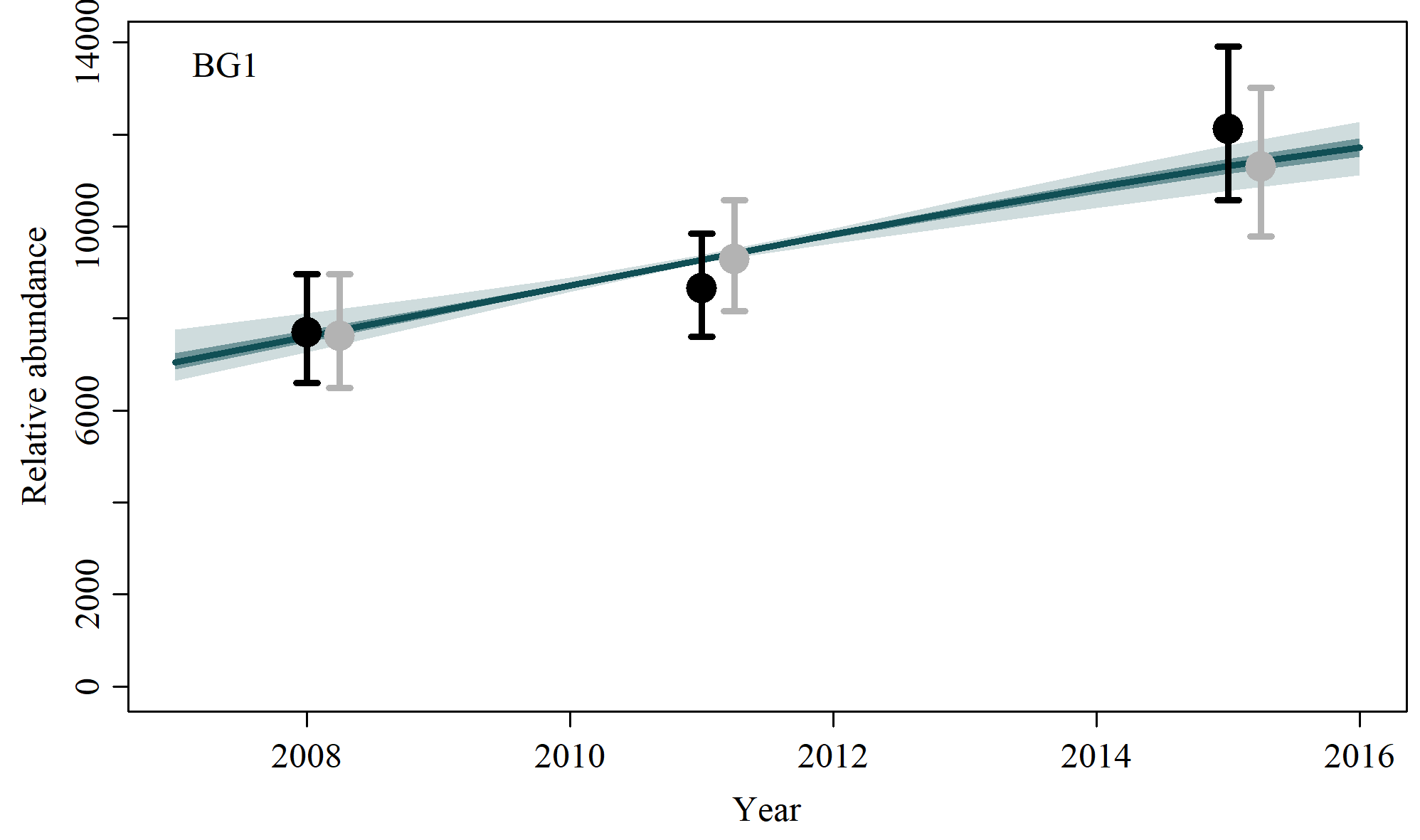
**

**4.7) Scenario D-5**

**
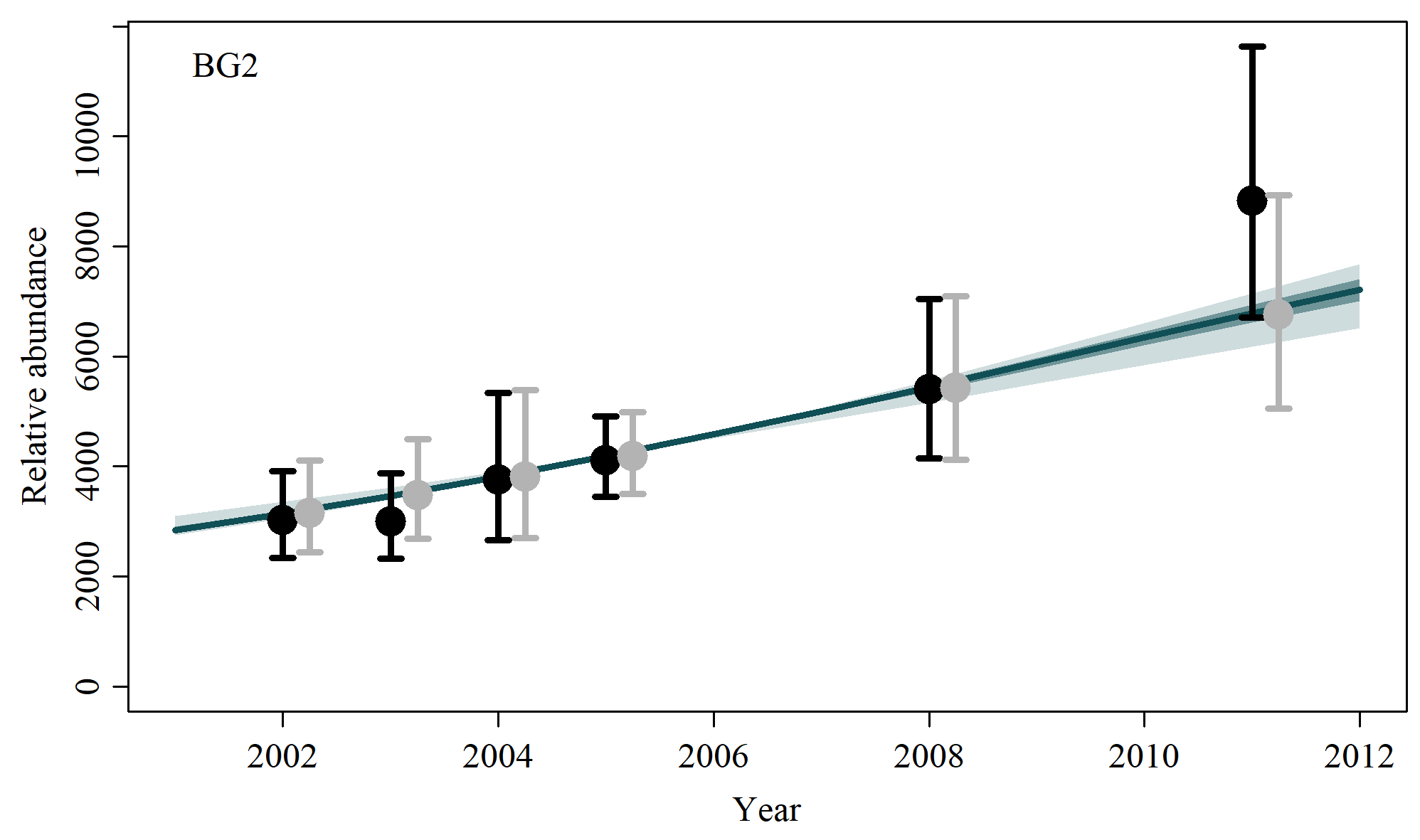
**

**4.8) Scenario D-6**

**
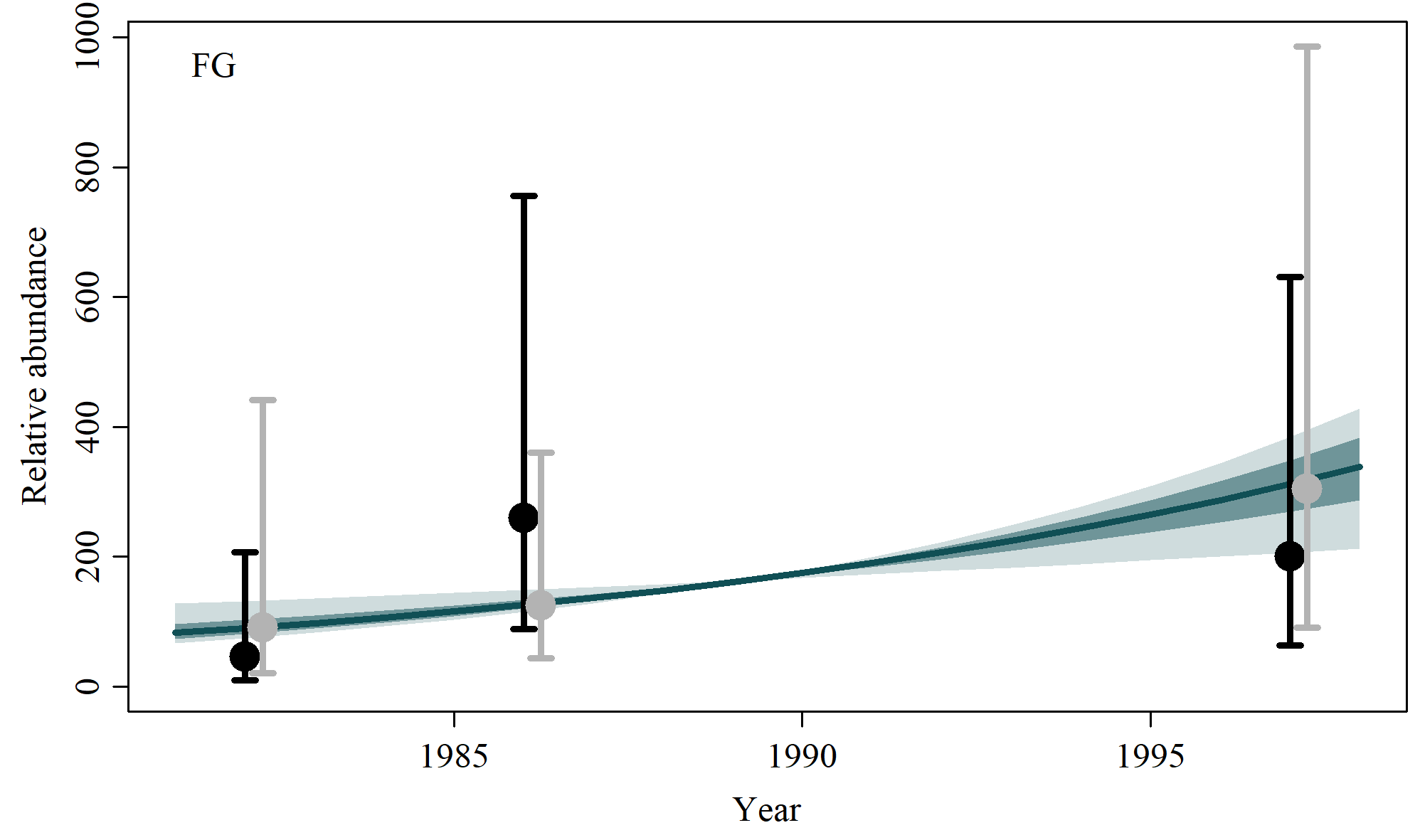
**

**4.9) Scenario D-7**

**
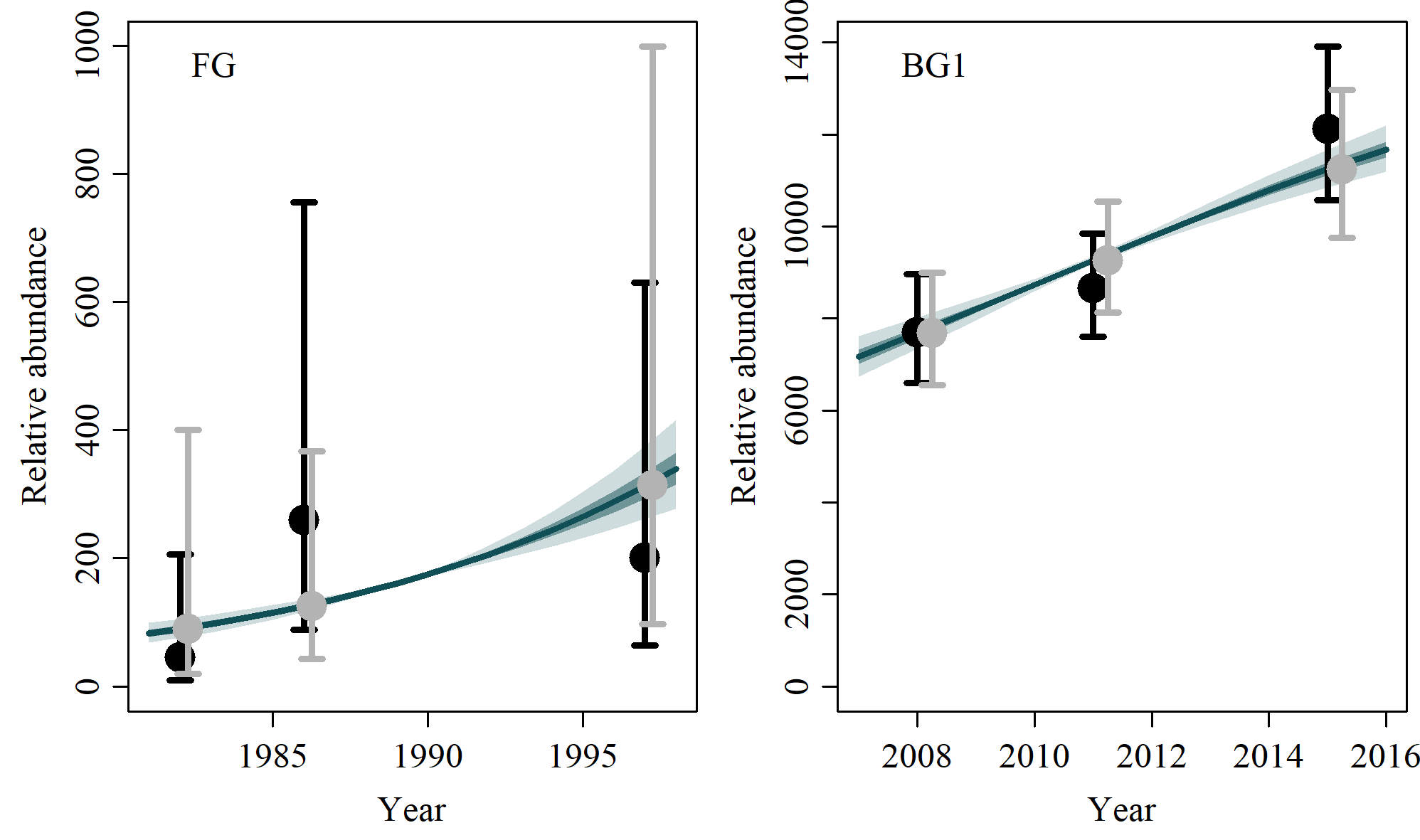
**

**4.10) Scenario C-1 to C-7**

| **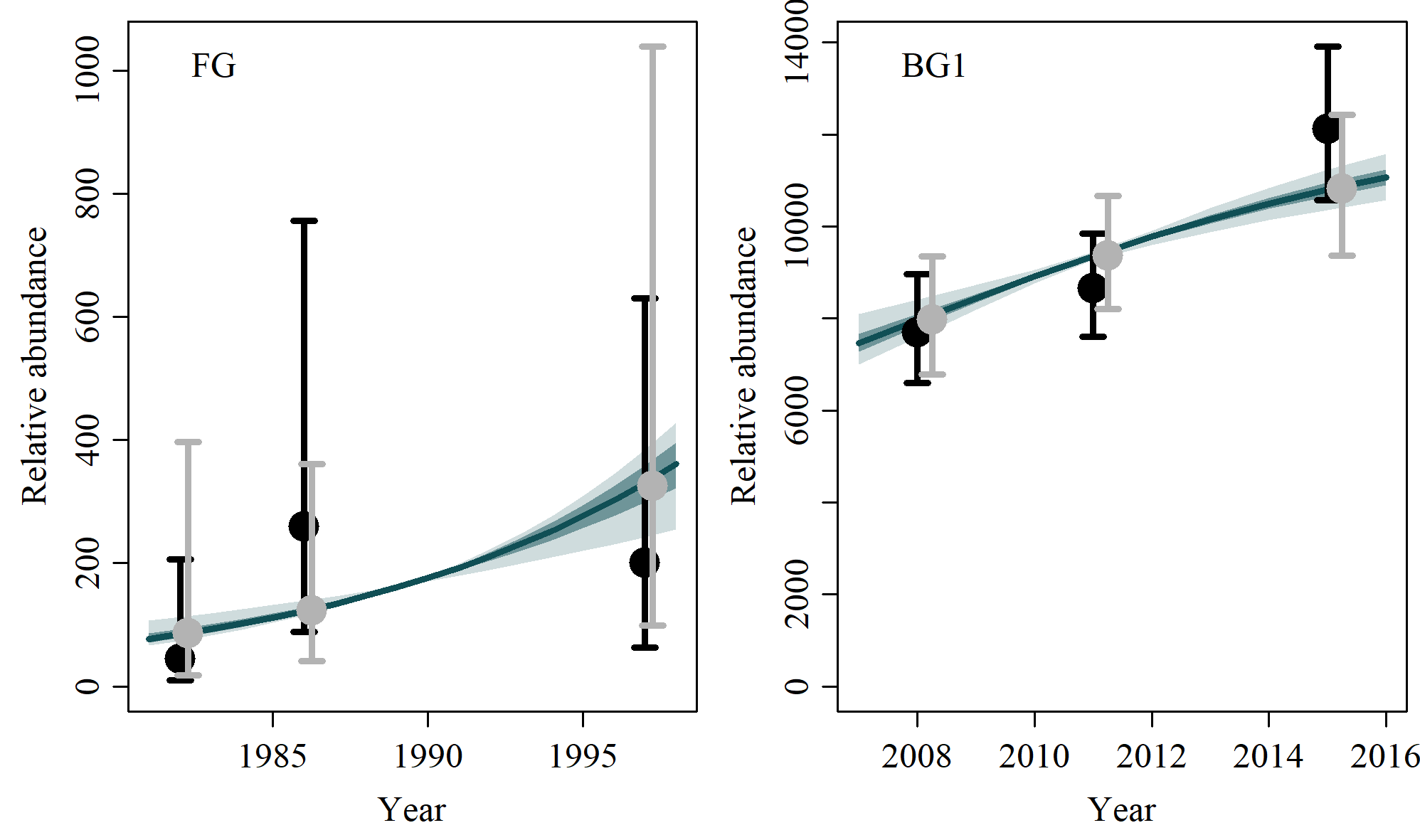** | **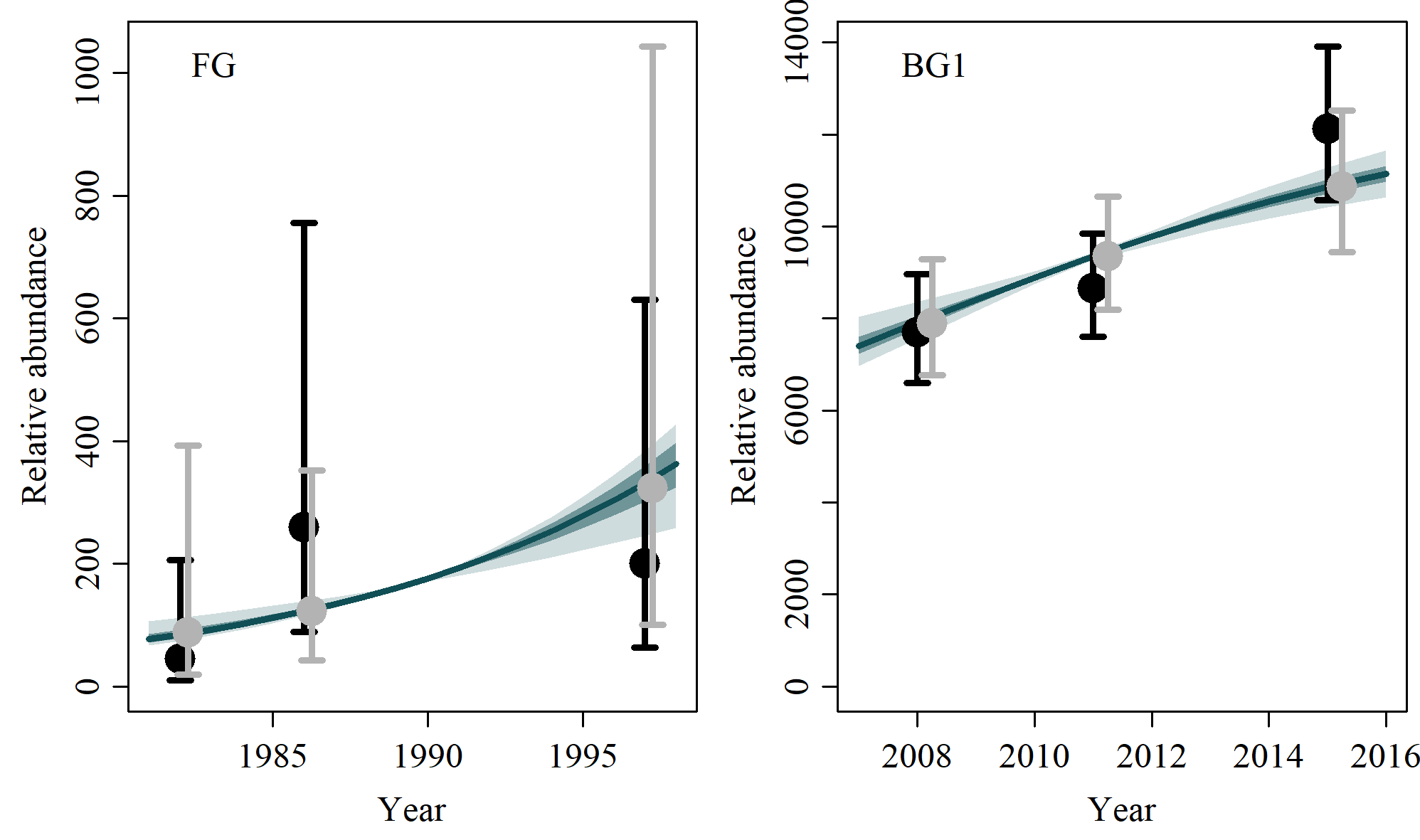** |
| --- | --- |
| C-1 | C-2 |
|  |  |
| 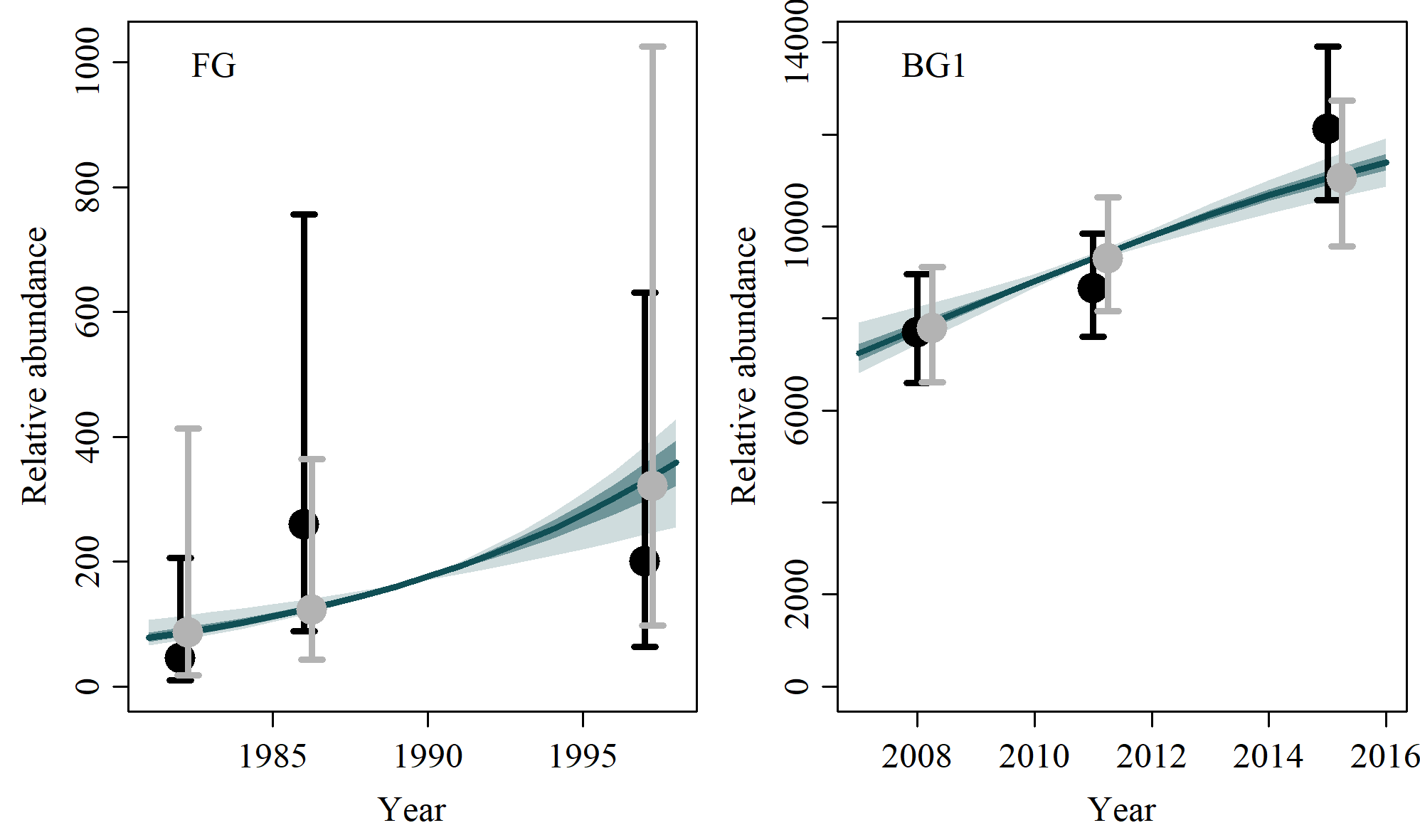 | 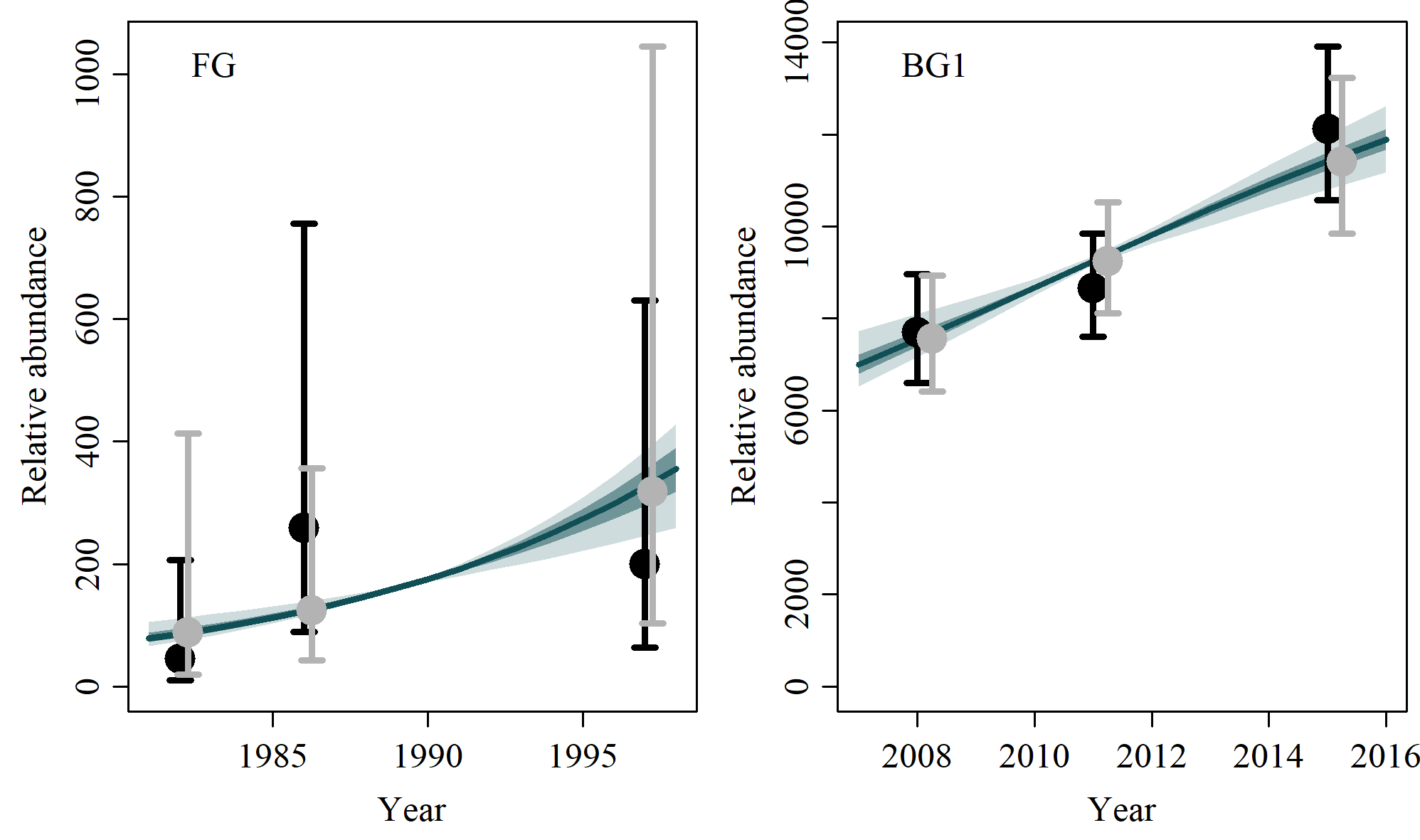 |
| C-3 | C-4 |
|  |  |
| 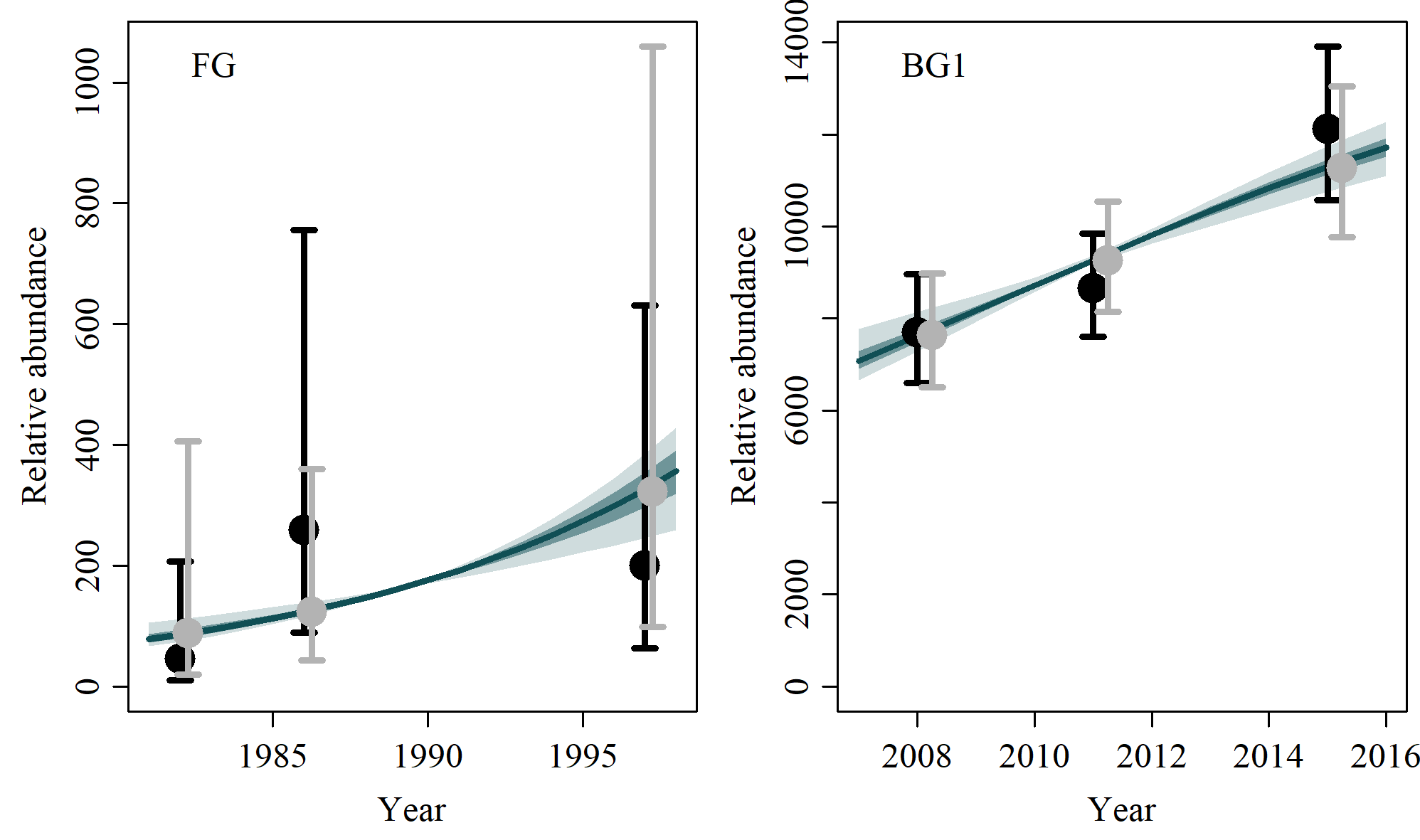 | 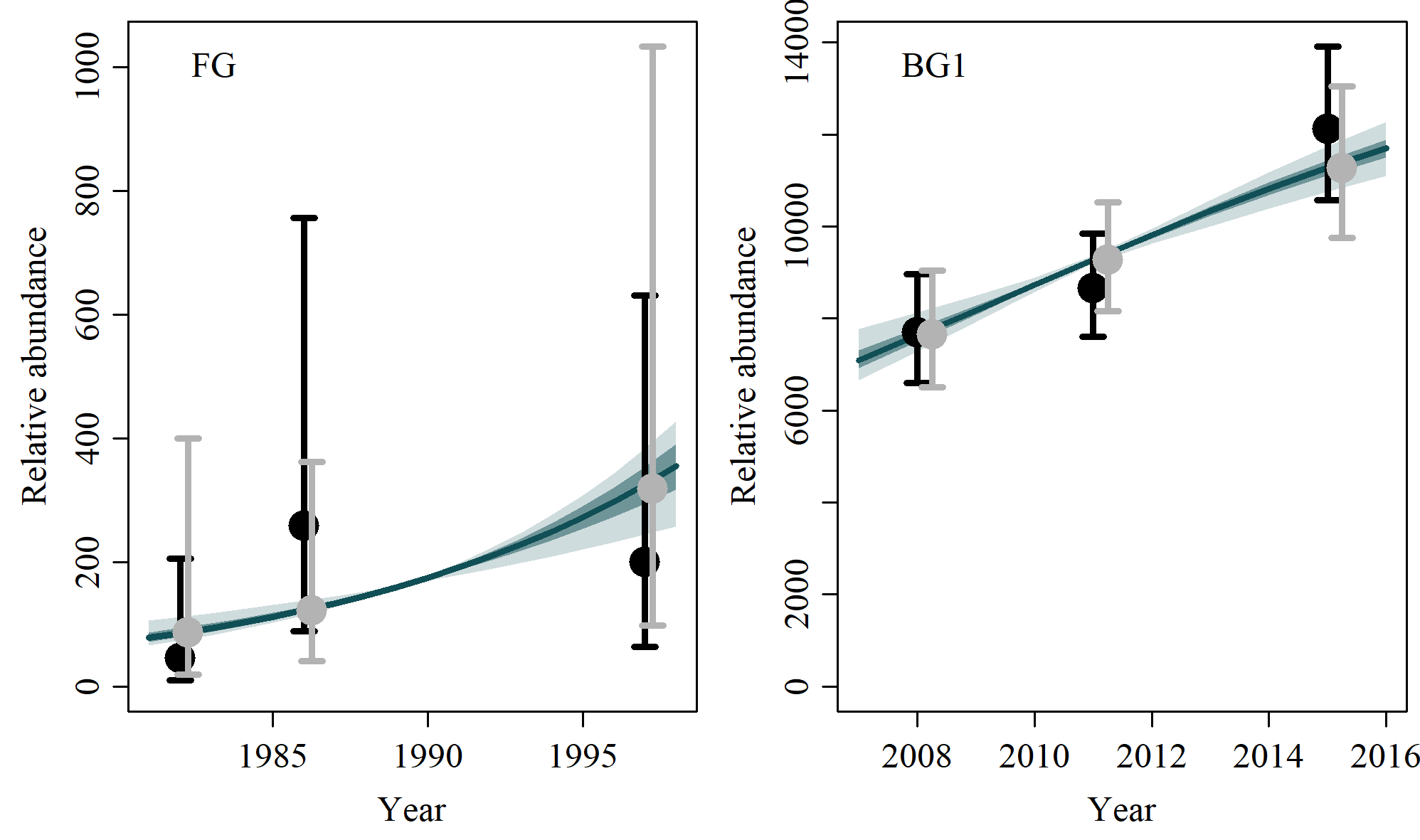 |
| C-5 | C-6 |
|  |  |
| 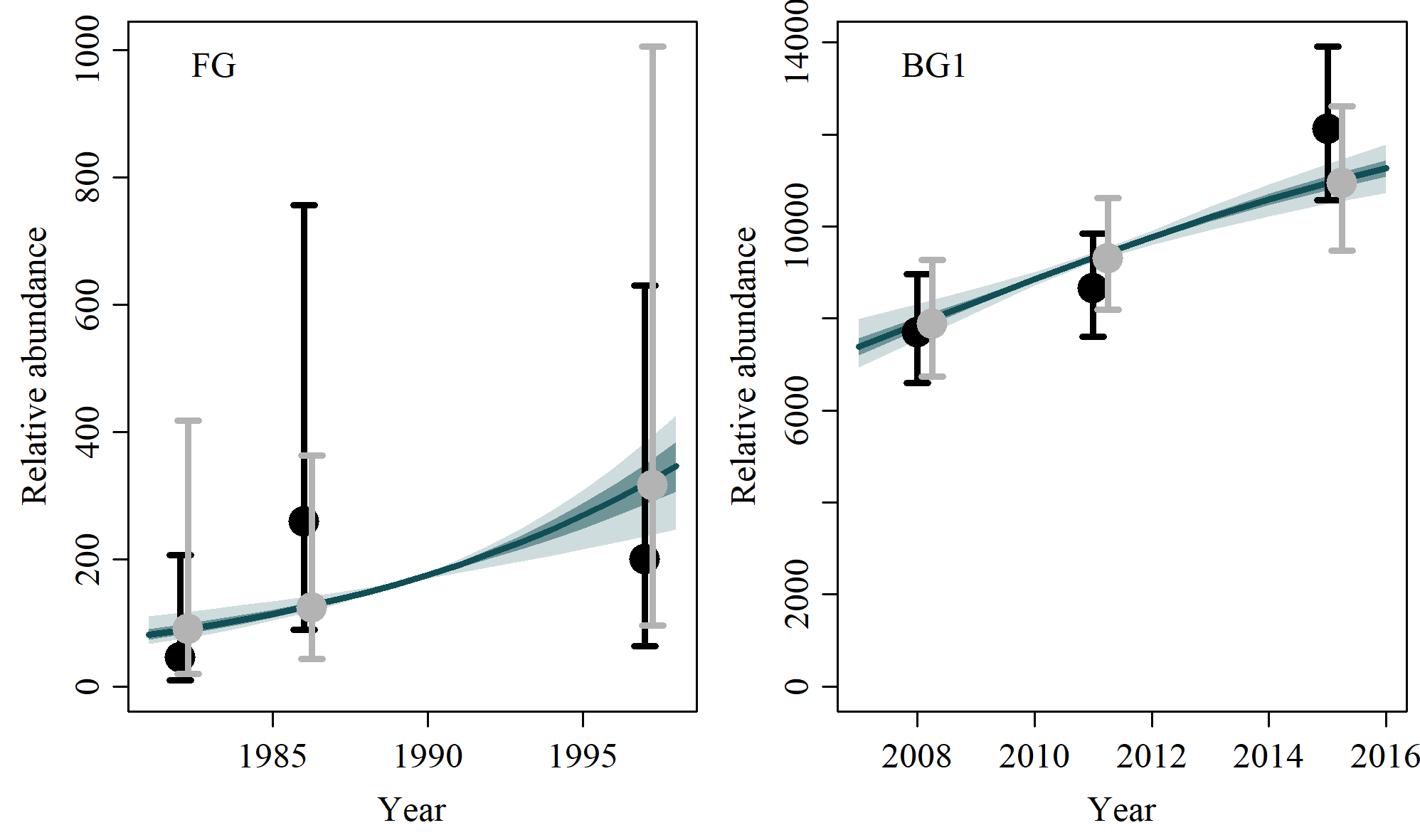 |  |
| C-7 |  |

**4.11) Scenario G-1, G2, M-1 and M-2**

| **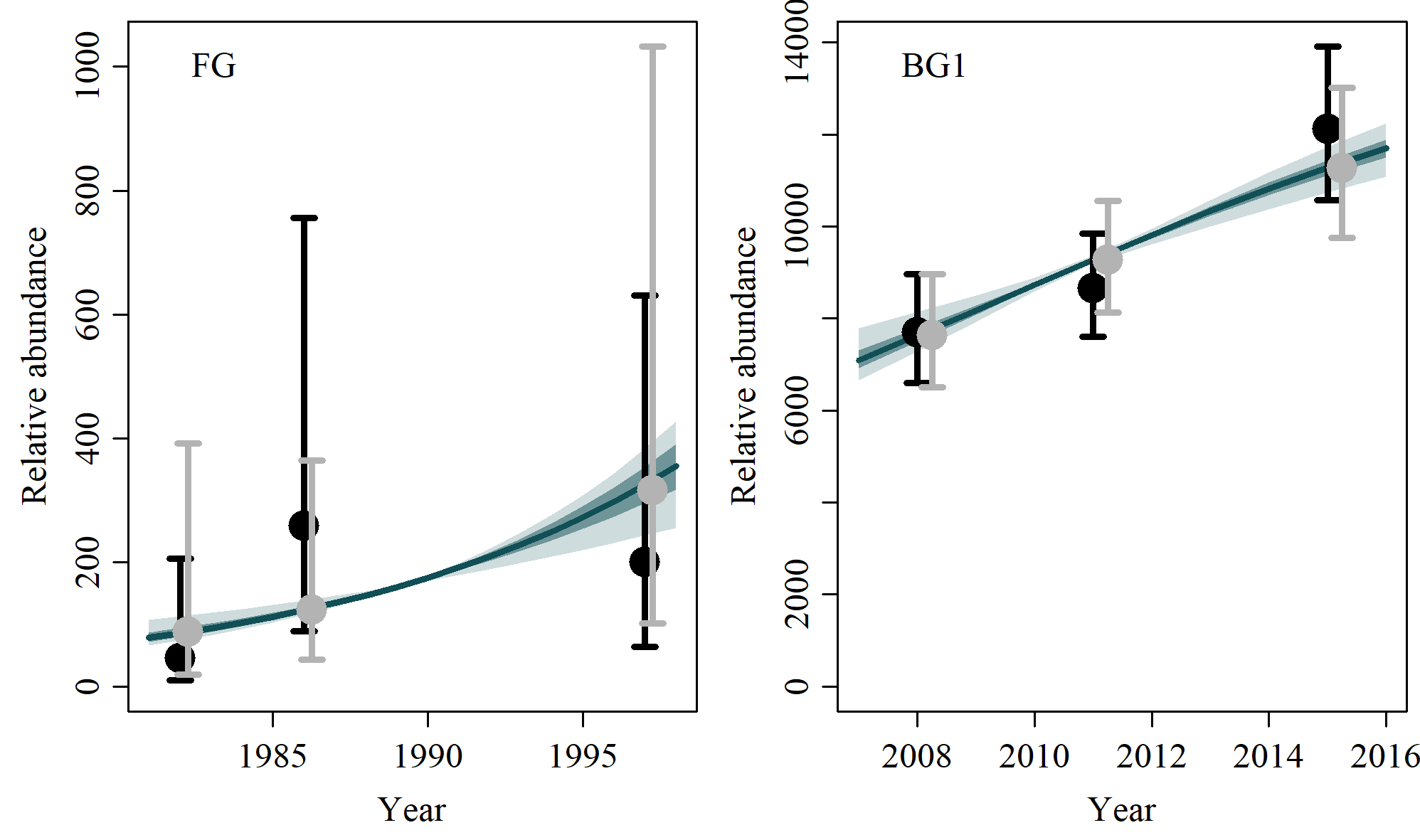** | **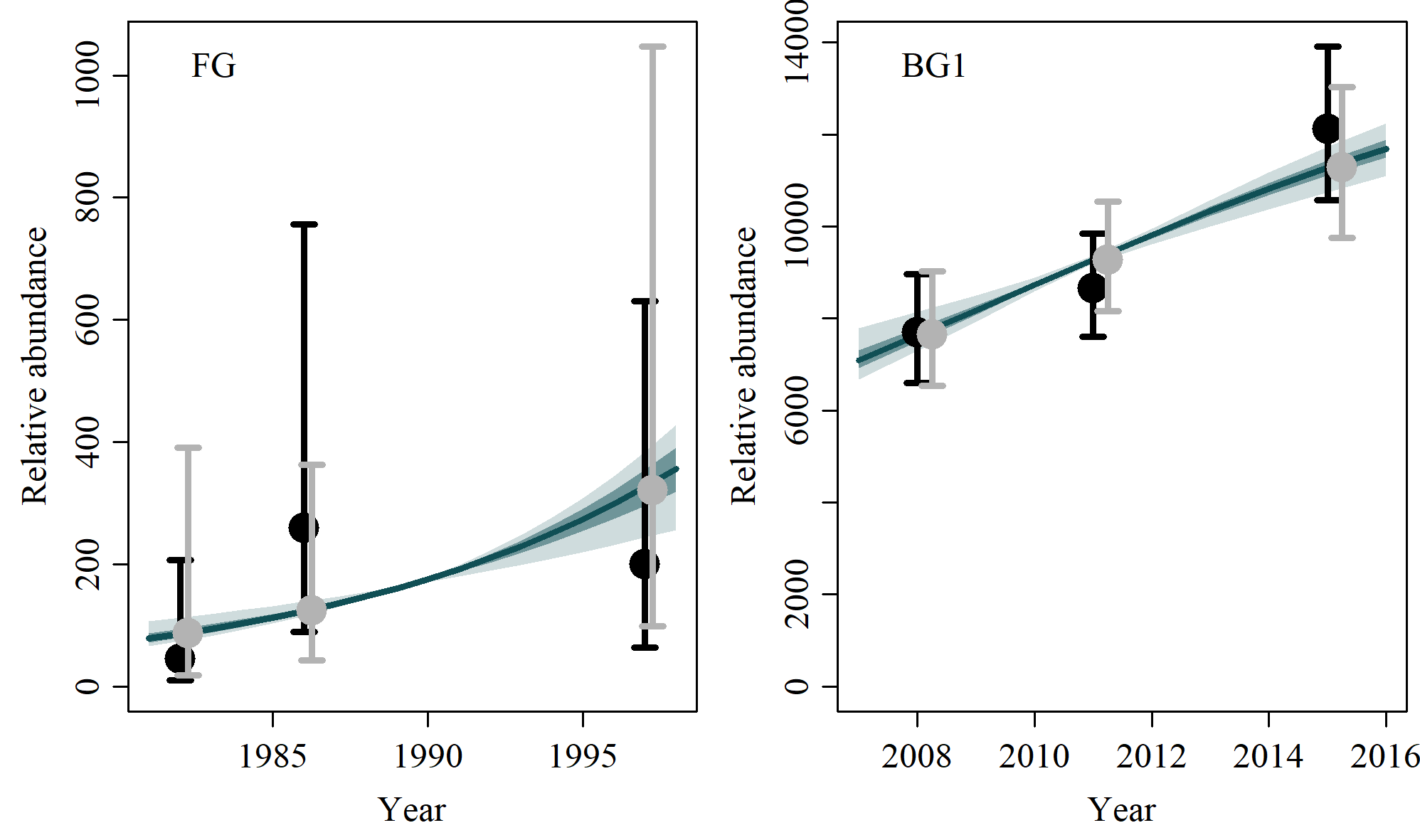** |
| --- | --- |
| G-1 | G-2 |
|  |  |
| 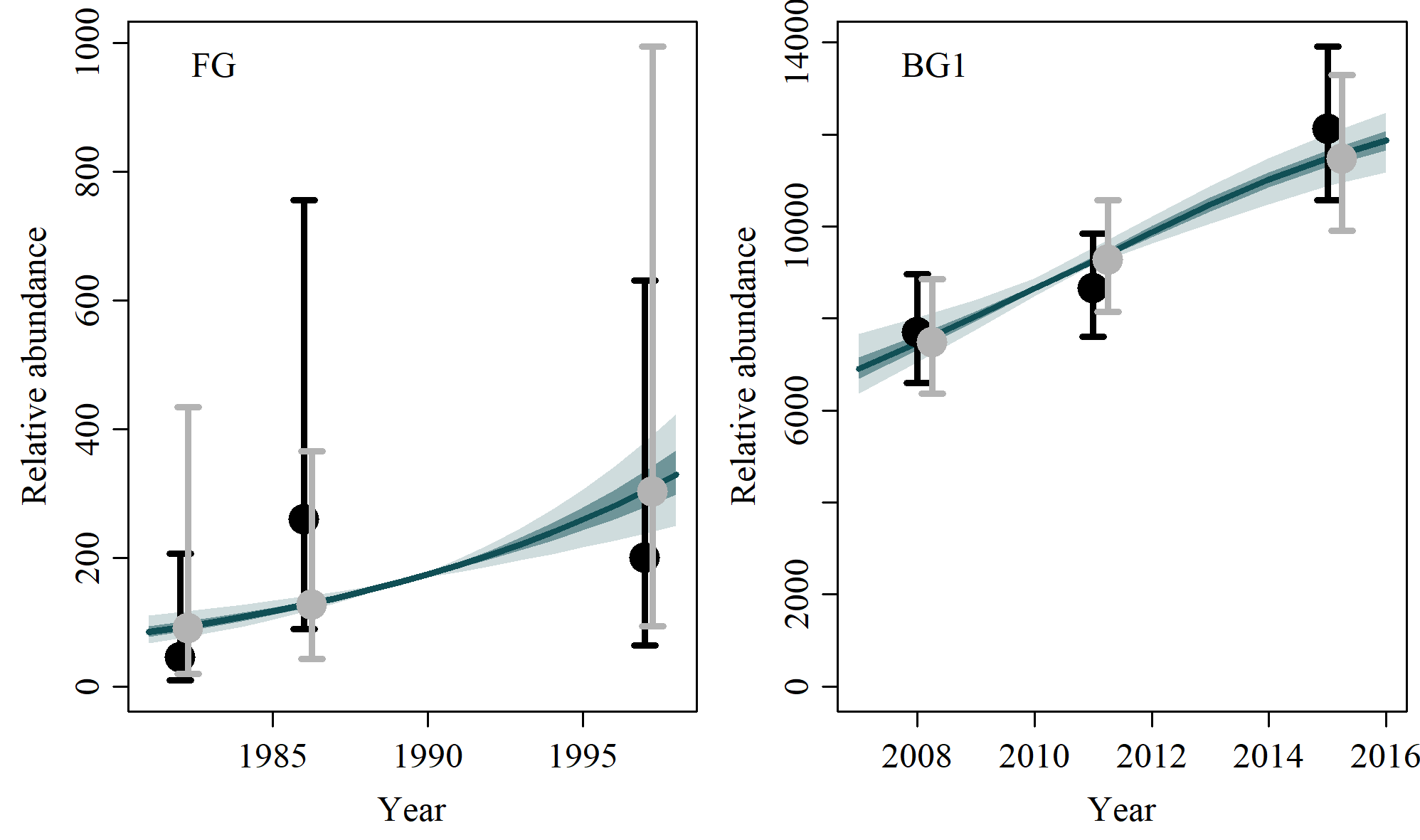 | 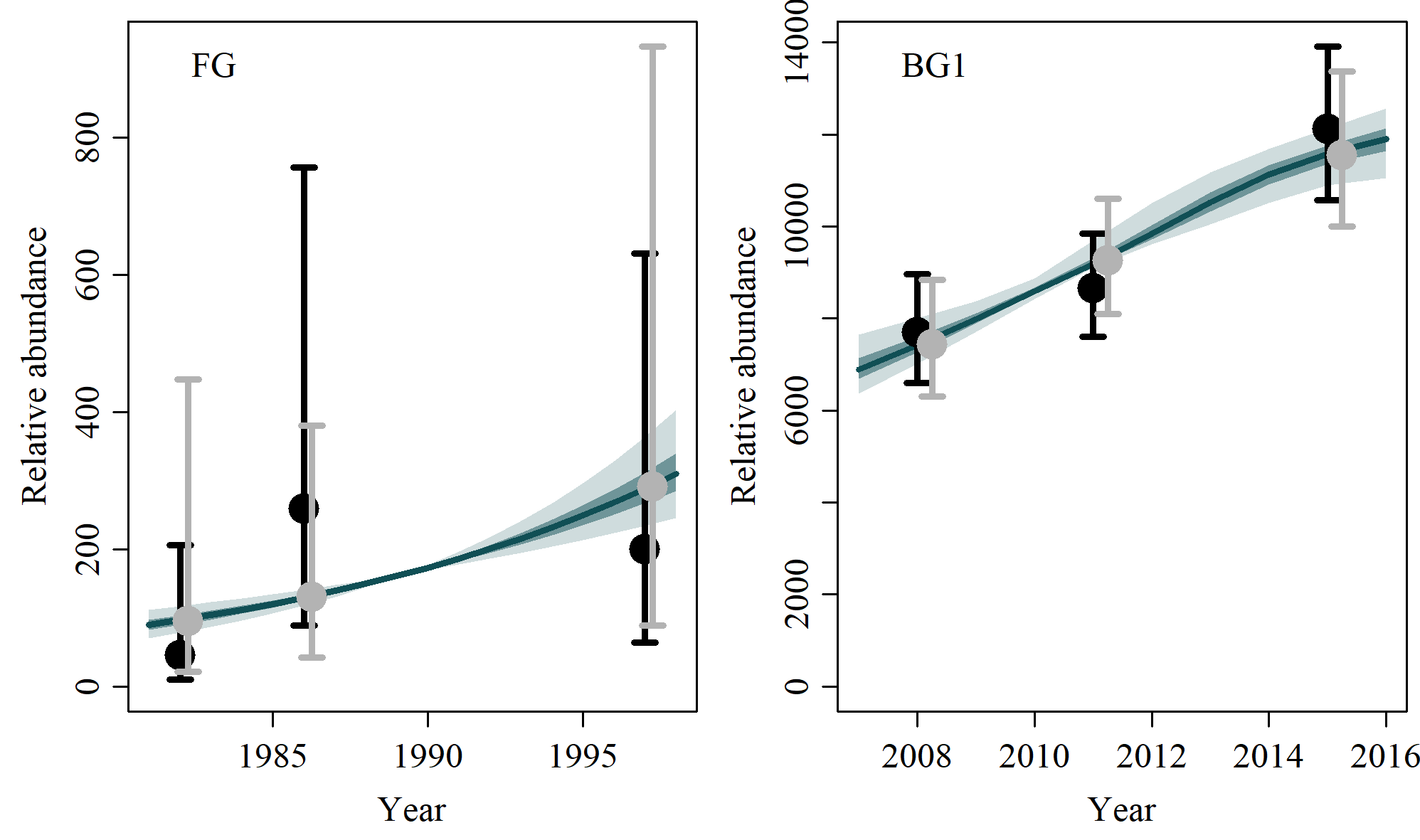 |
| M-1 | M-2 |

**5) Posterior Density Plots**

**5.1) Model average**

**
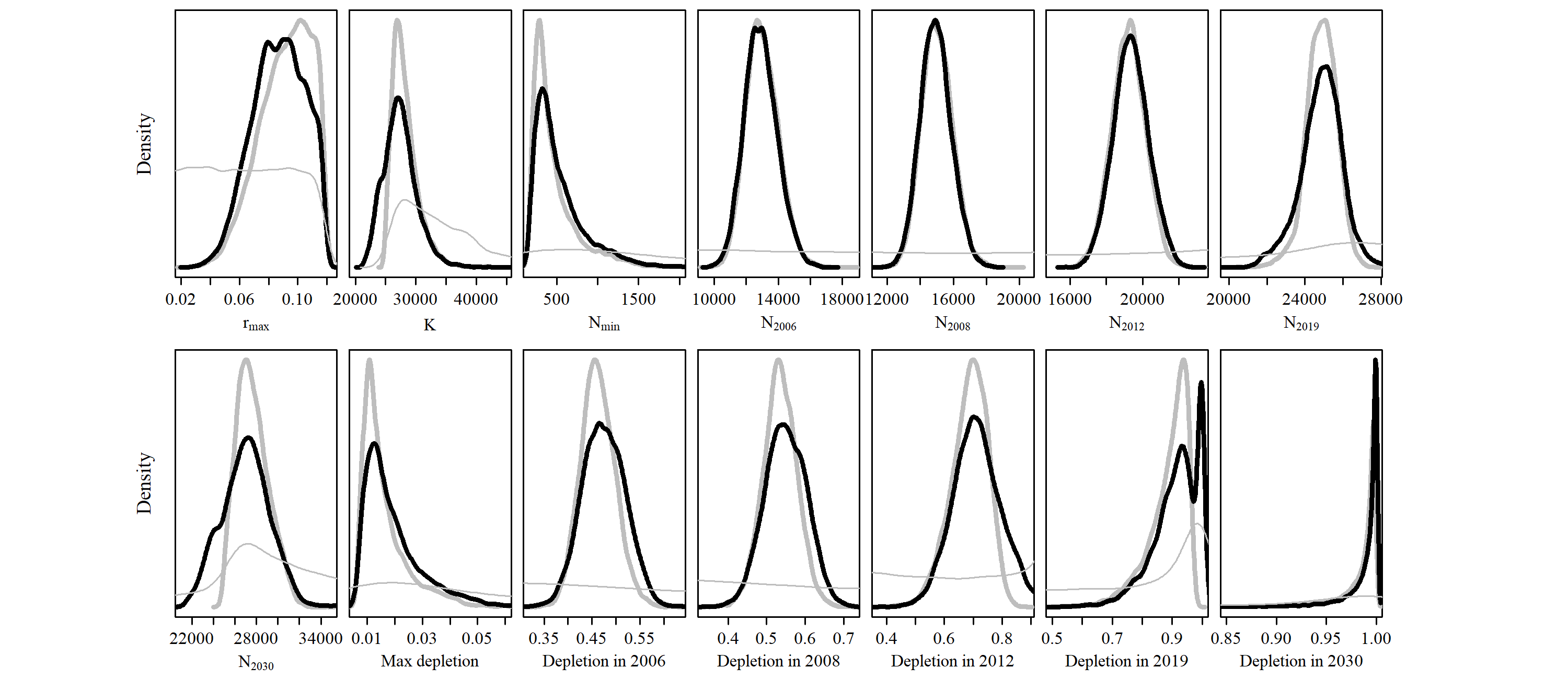
**

**5.2) Reference Case**

**
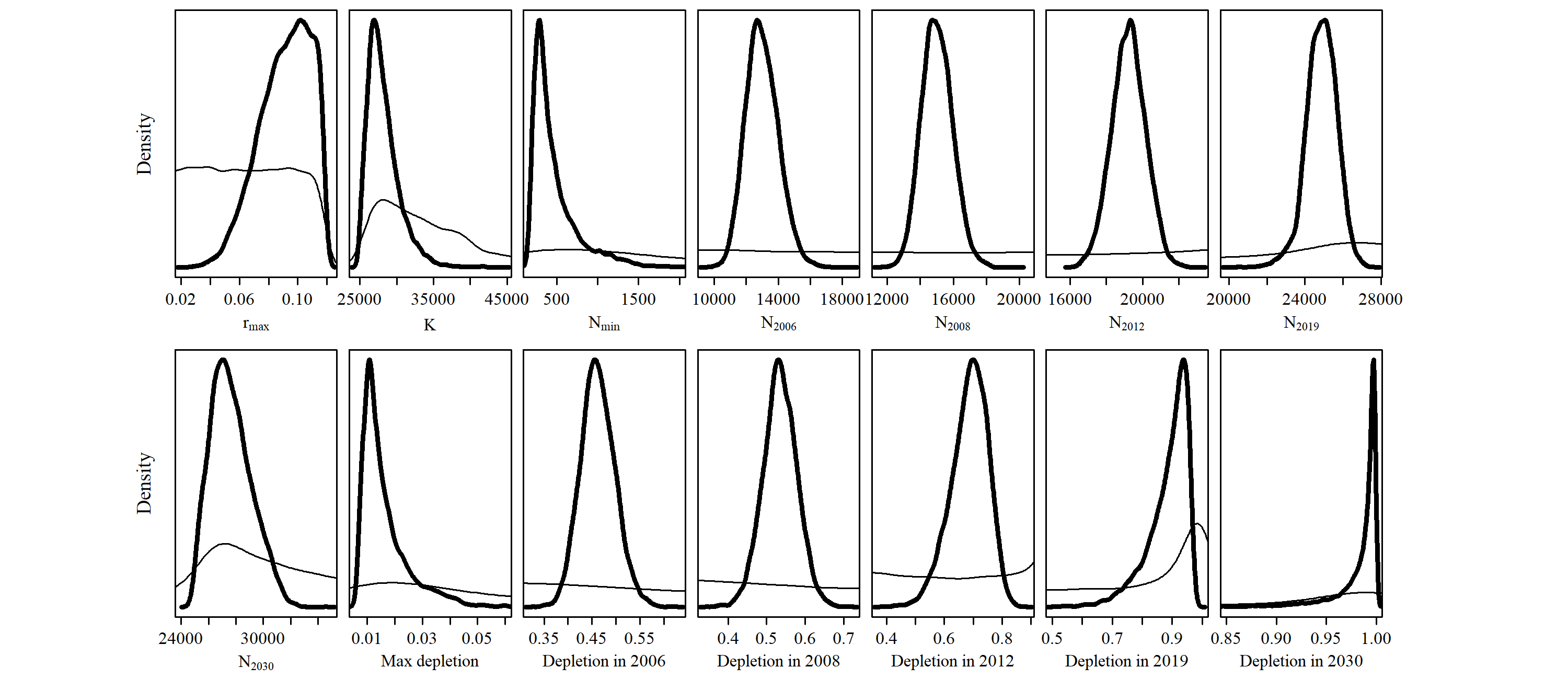
**

**5.3) Scenario D-1**

**
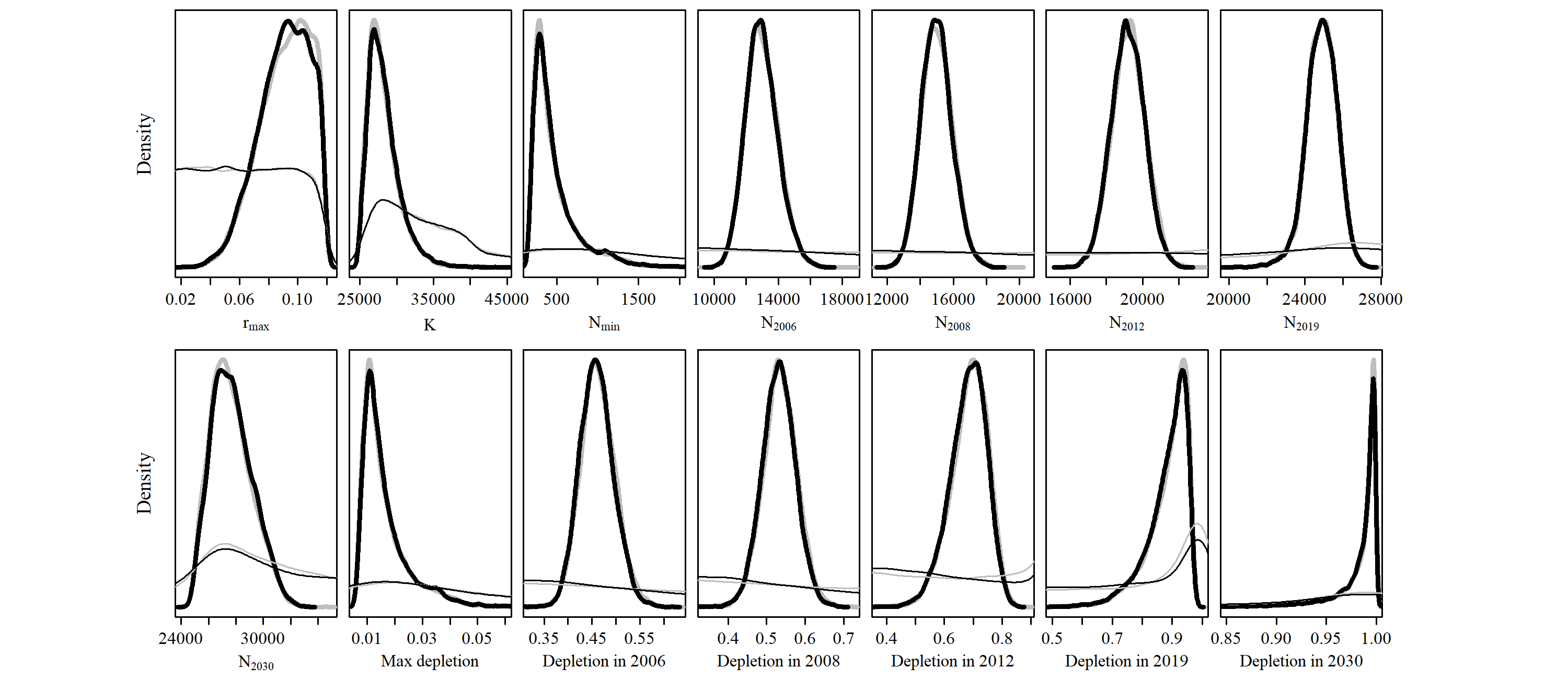
**

**5.4) Scenario D-2**

**
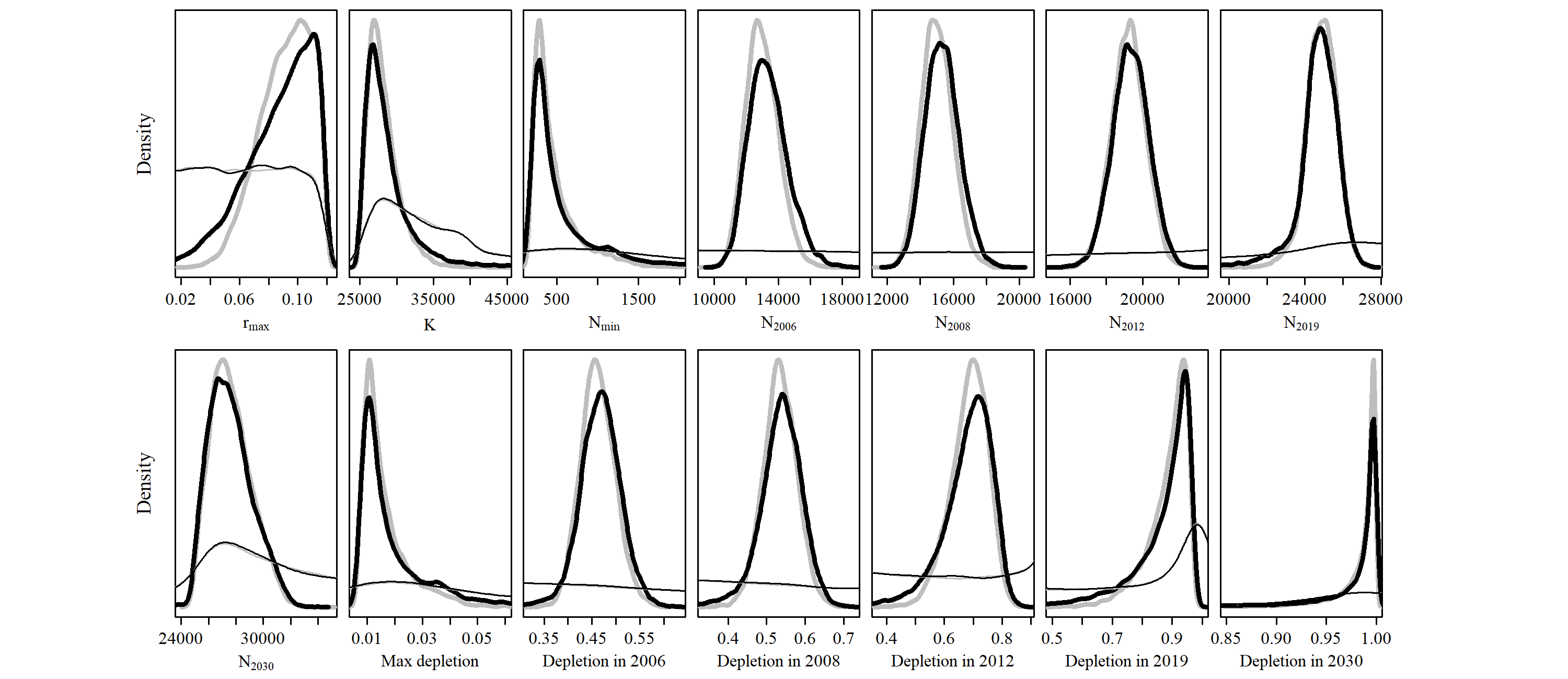
**

**5.5) Scenario D-3**

**
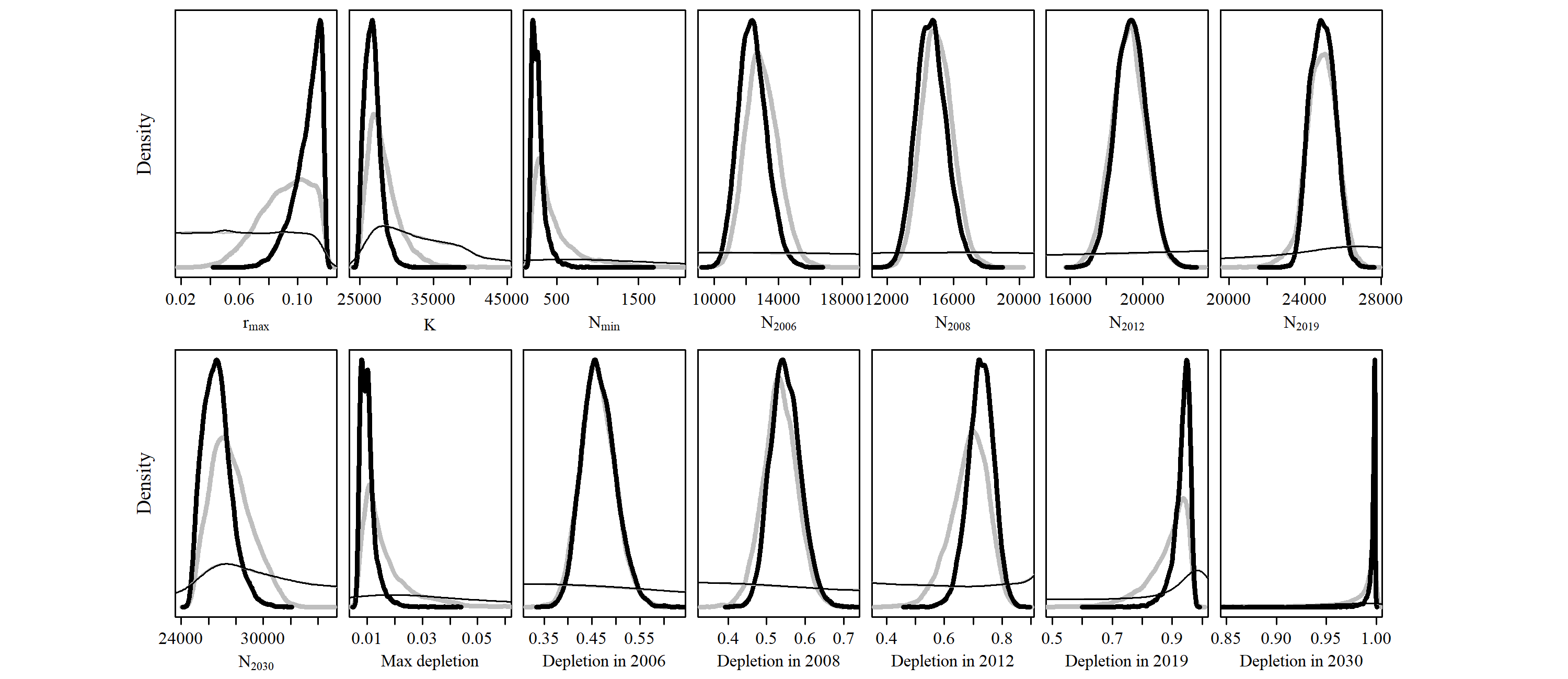
**

**5.6) Scenario D-4**

**
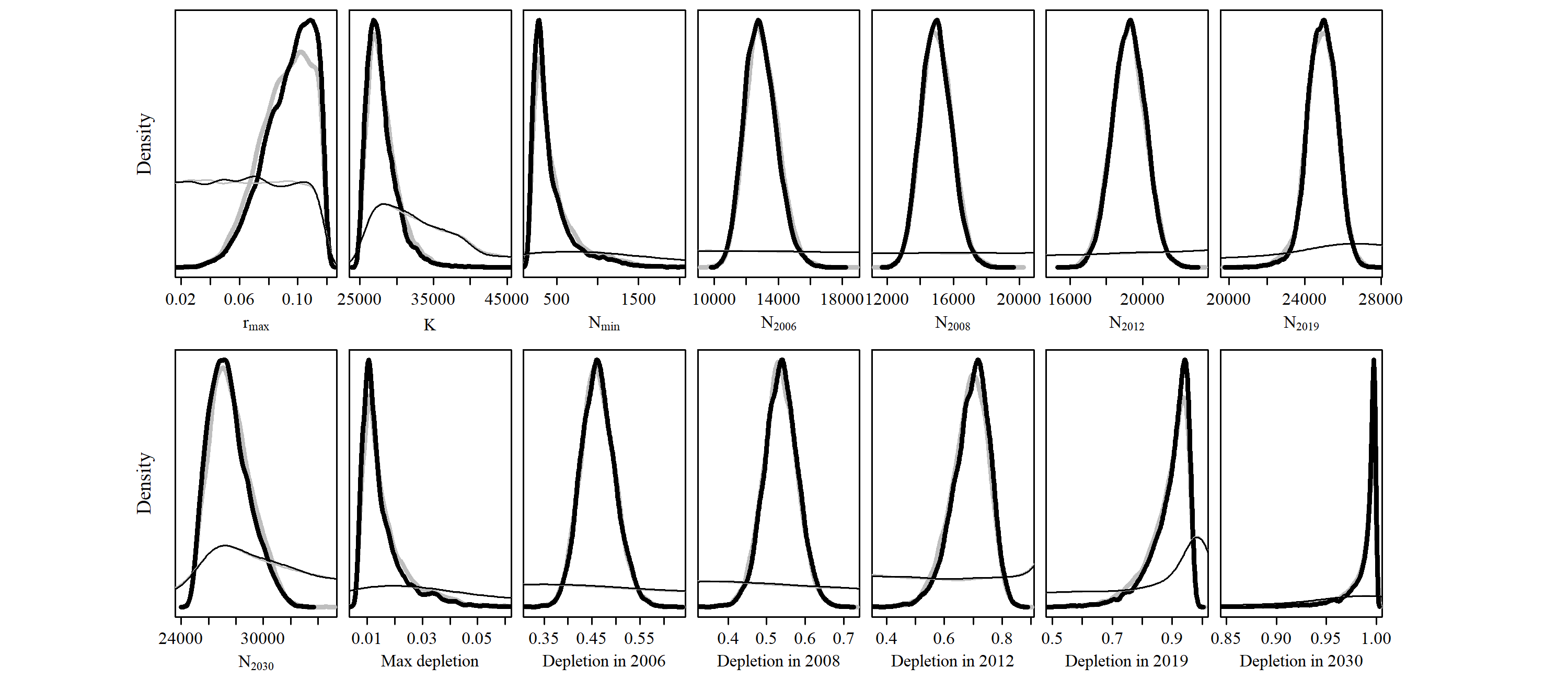
**

**5.7) Scenario D-5**

**
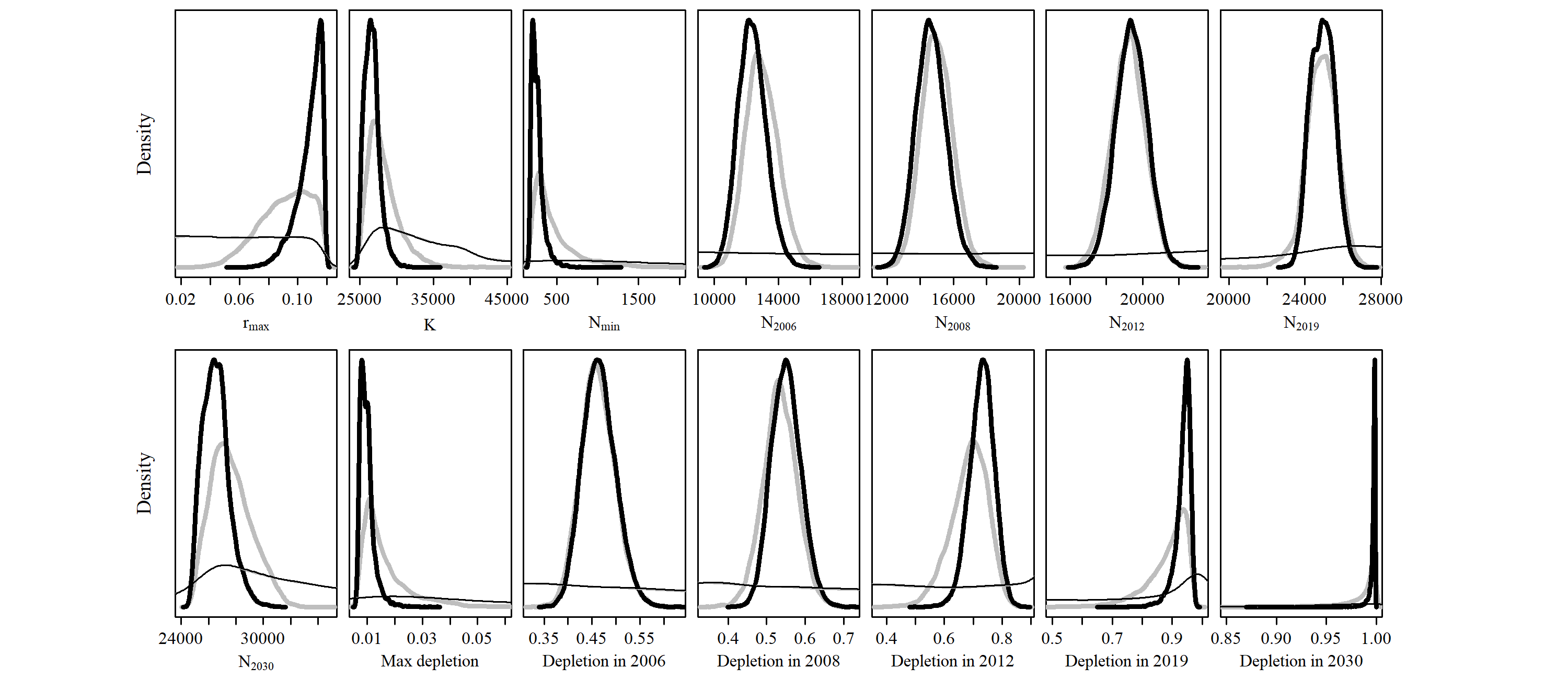
**

**5.8) Scenario D-6**

**
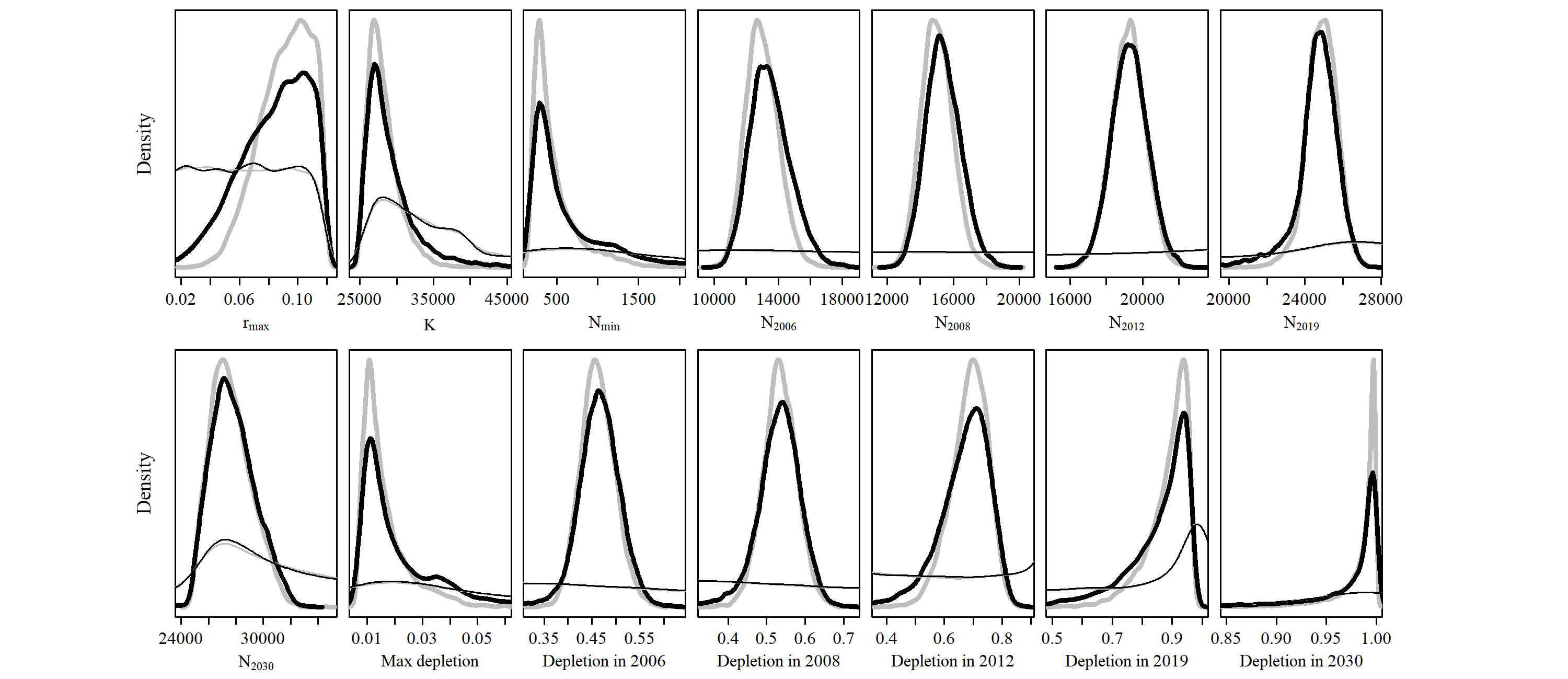
**

**5.9) Scenario D-7**

**
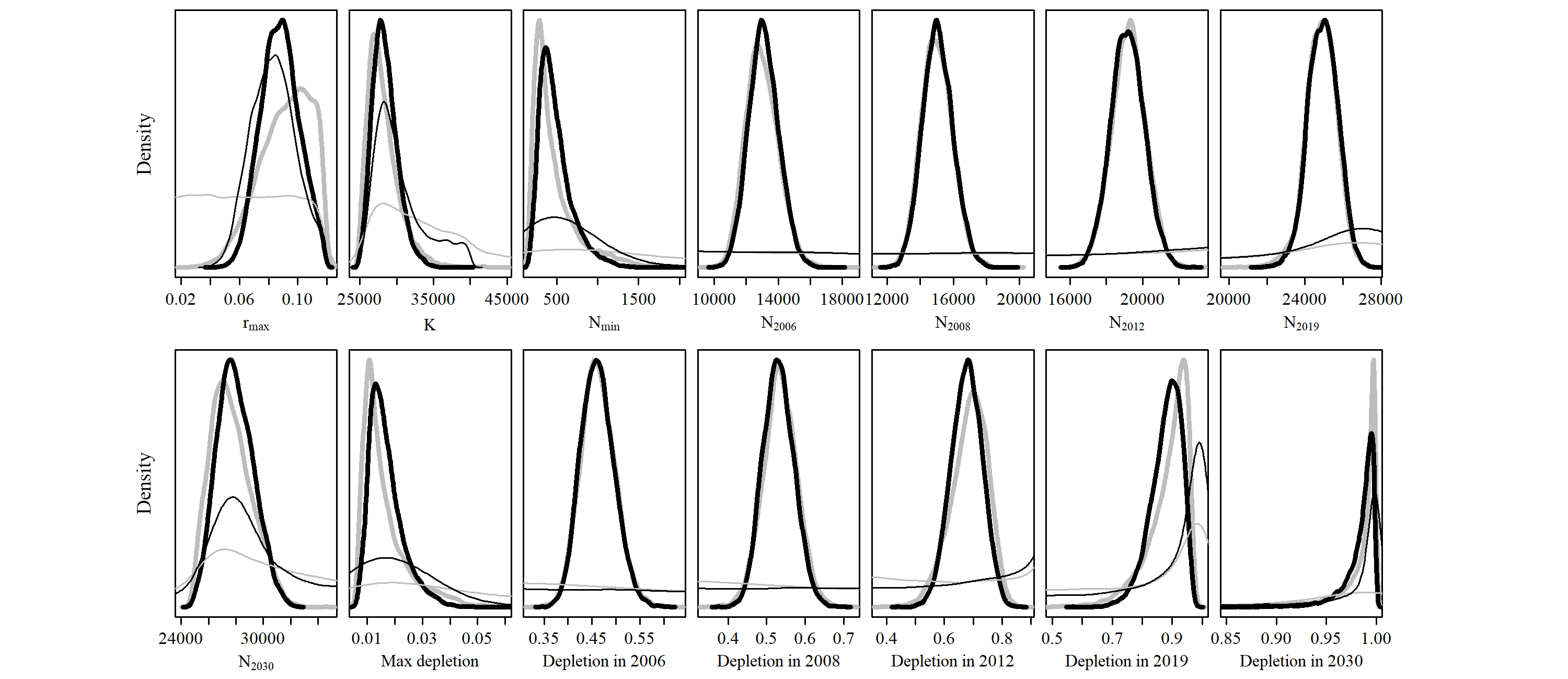
**

**5.10) Scenario C-1**

**
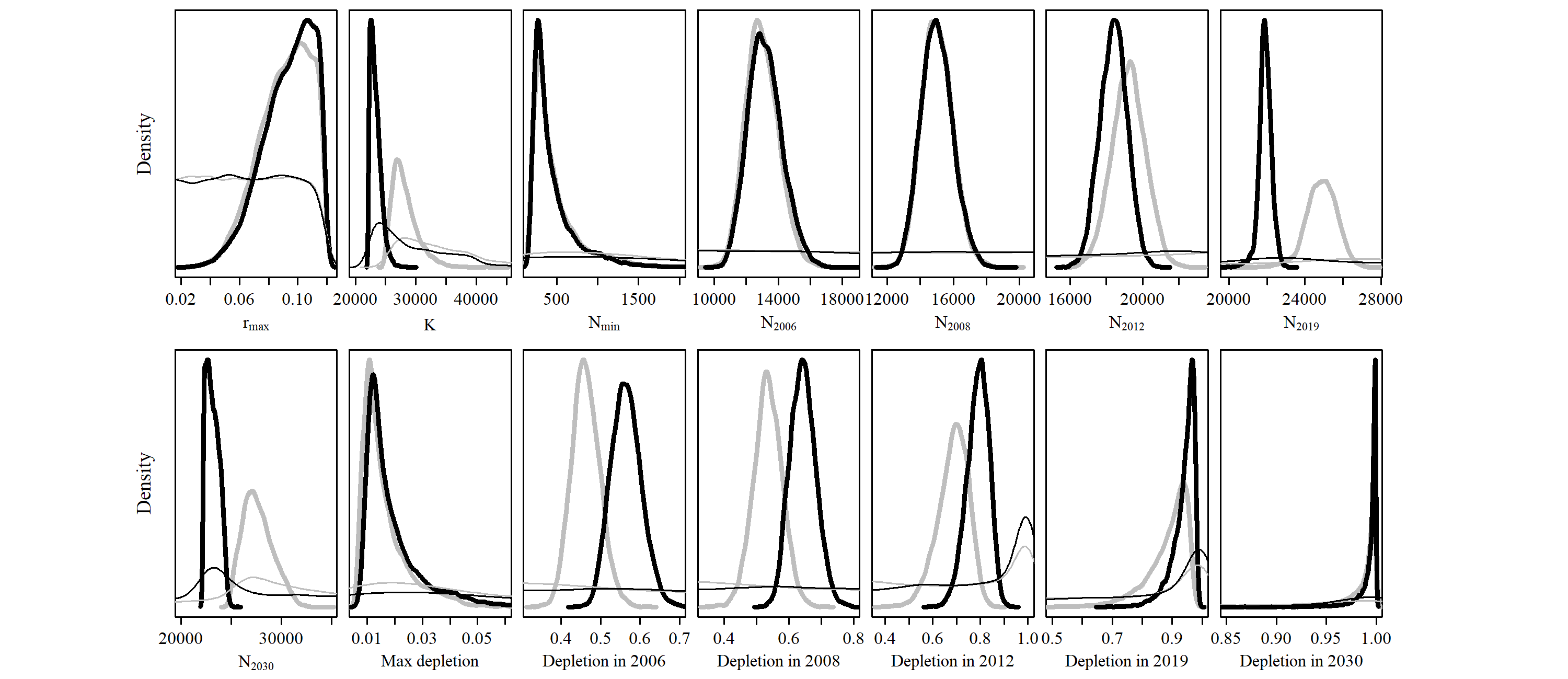
**

**5.11) Scenario C-2**

**
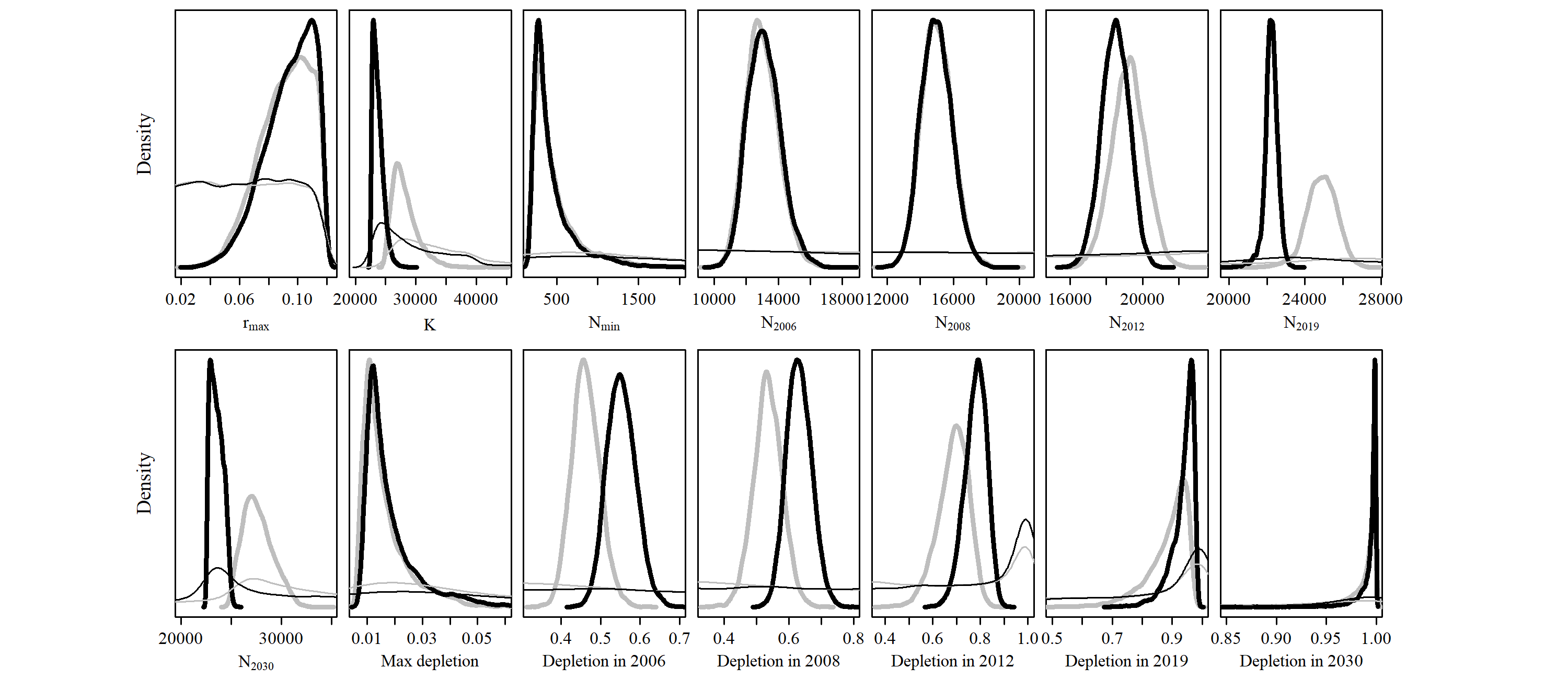
**

**5.12) Scenario C-3**

**
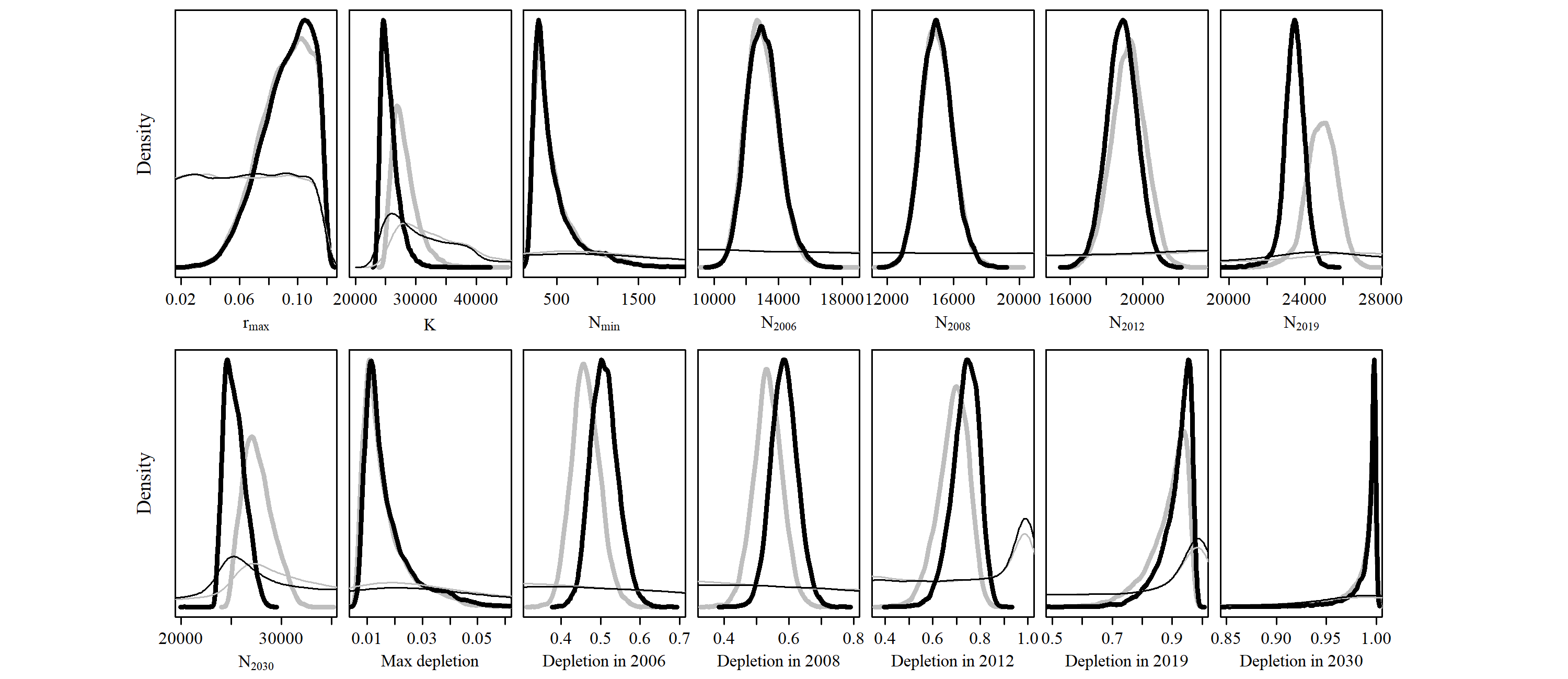
**

**5.13) Scenario C-4**

**
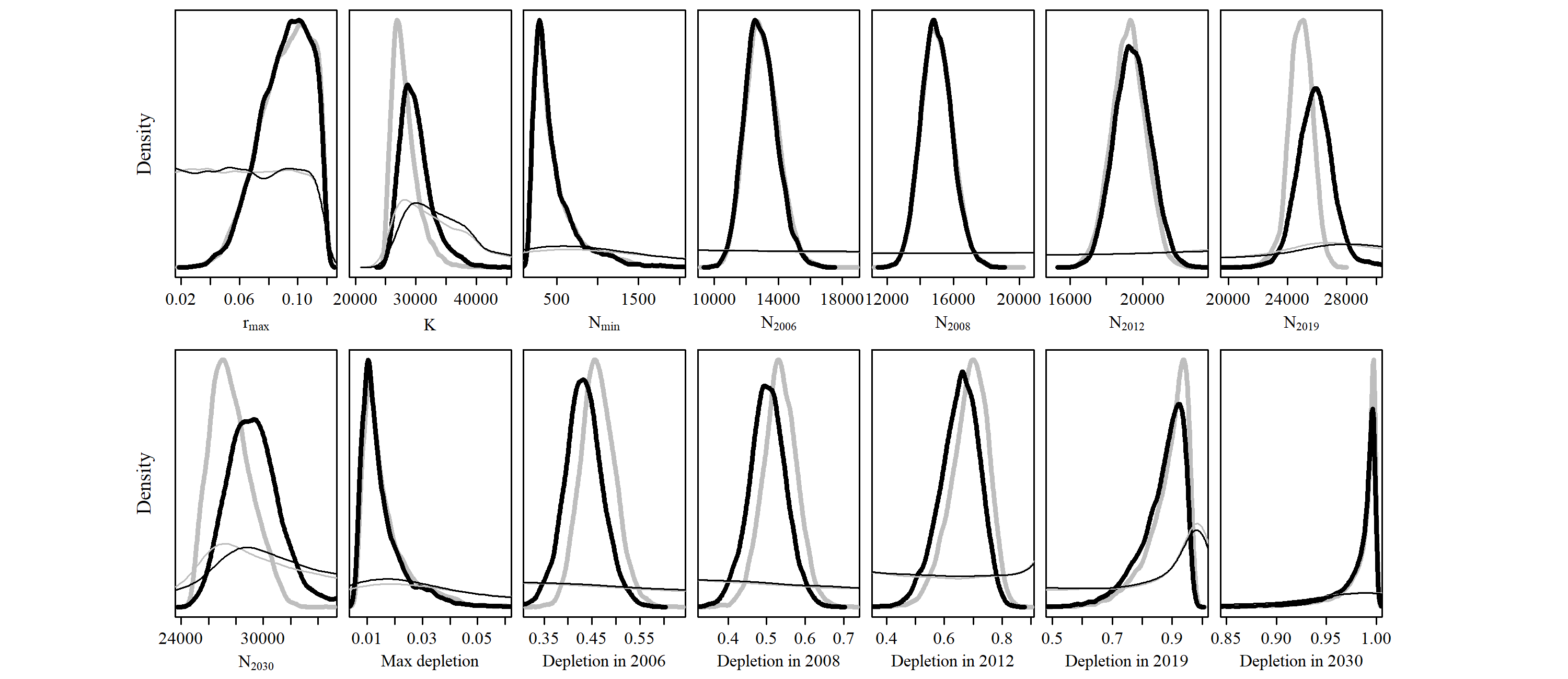
**

**5.14) Scenario C-5**

**
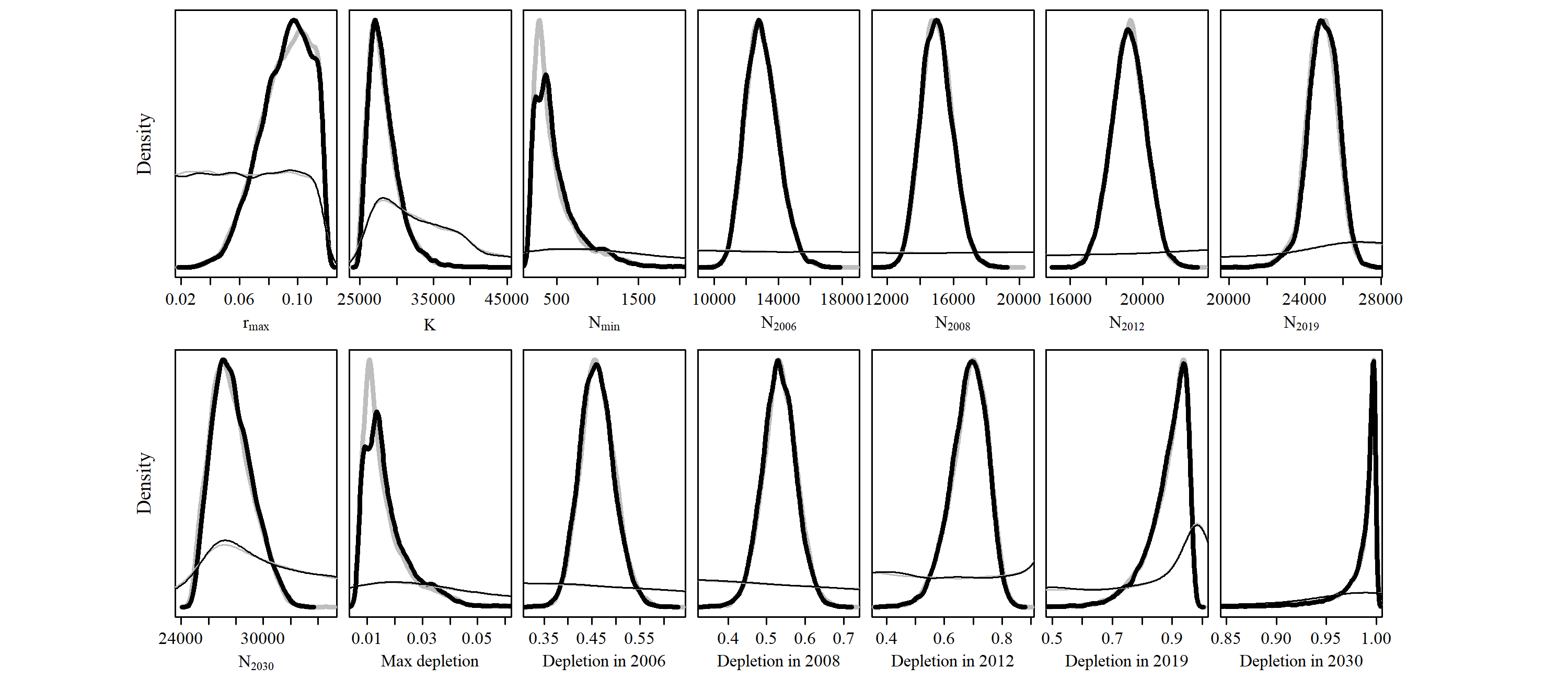
**

**5.15) Scenario C-6**

**
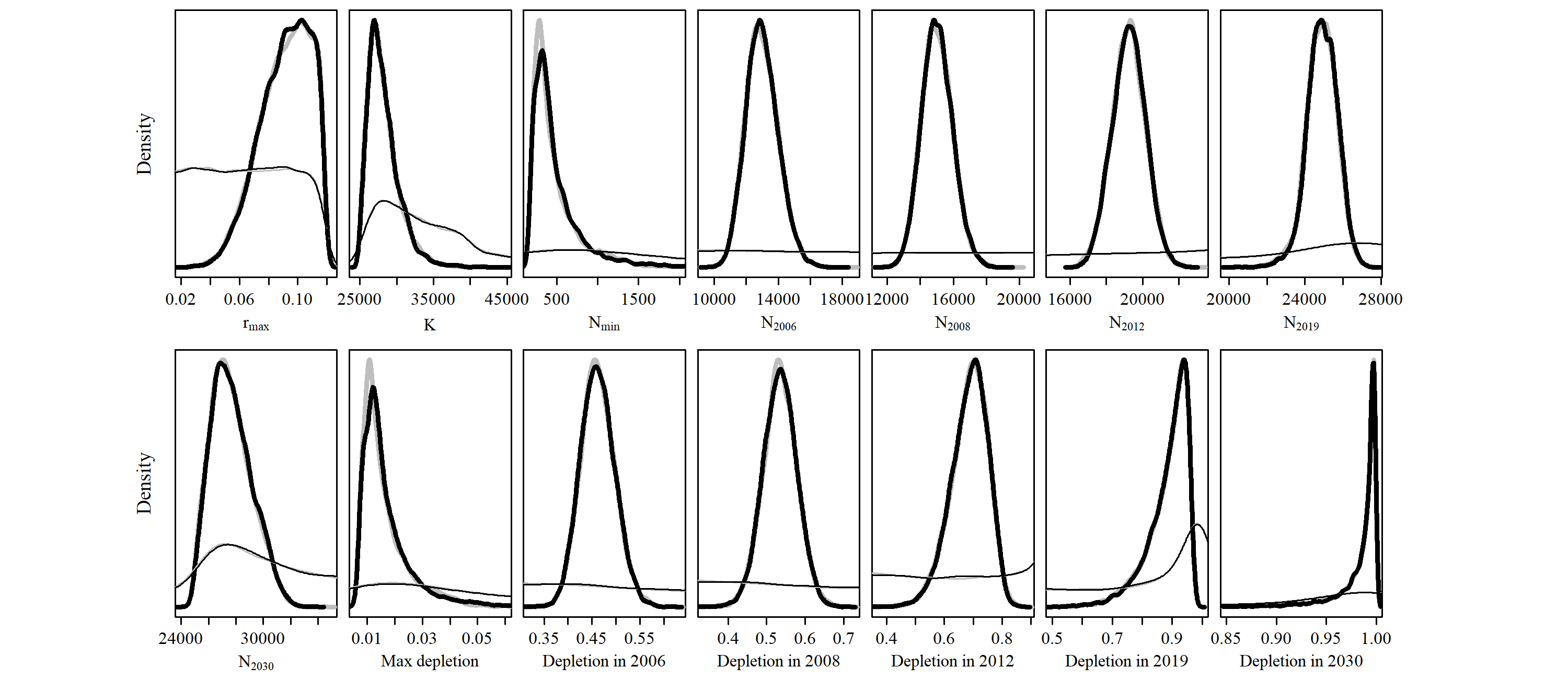
**

**5.16) Scenario C-7**

**
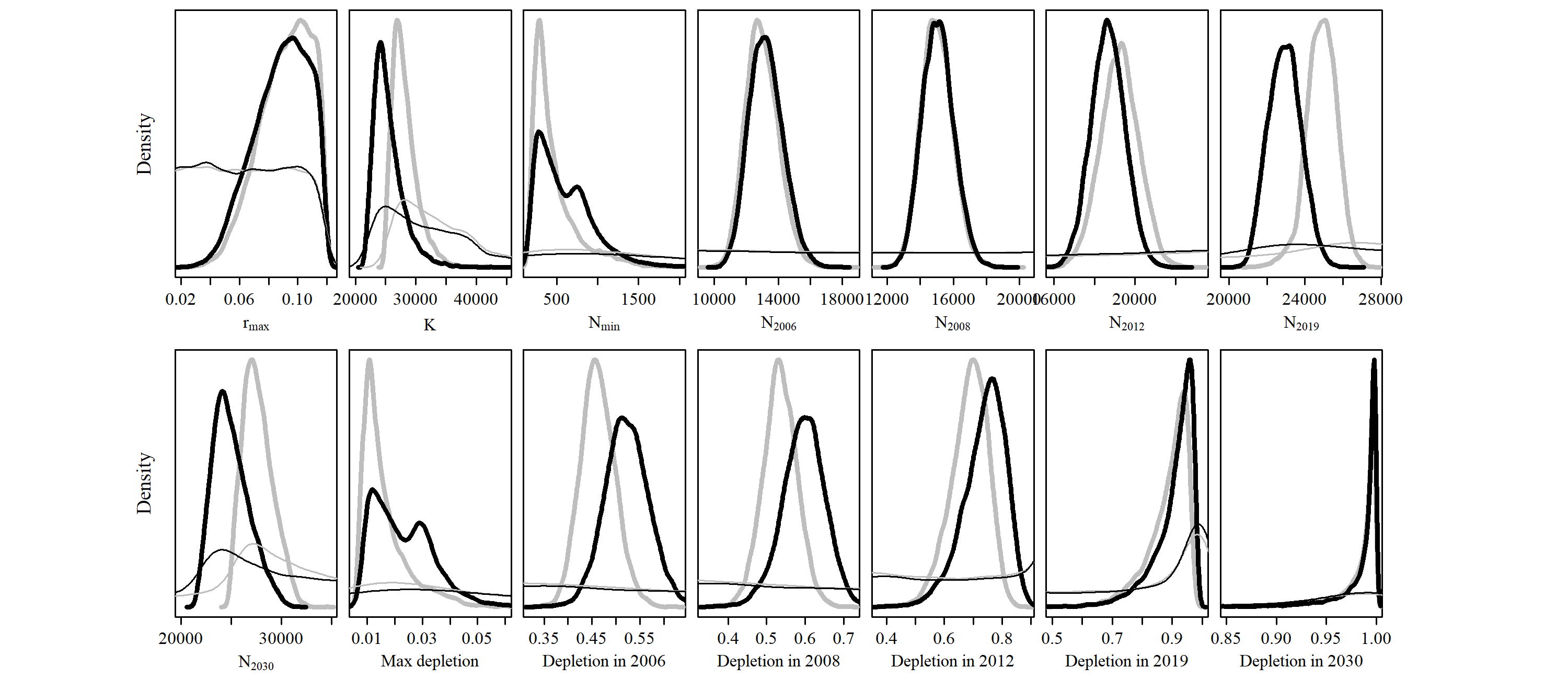
**

**5.17) Scenario G-1**

**
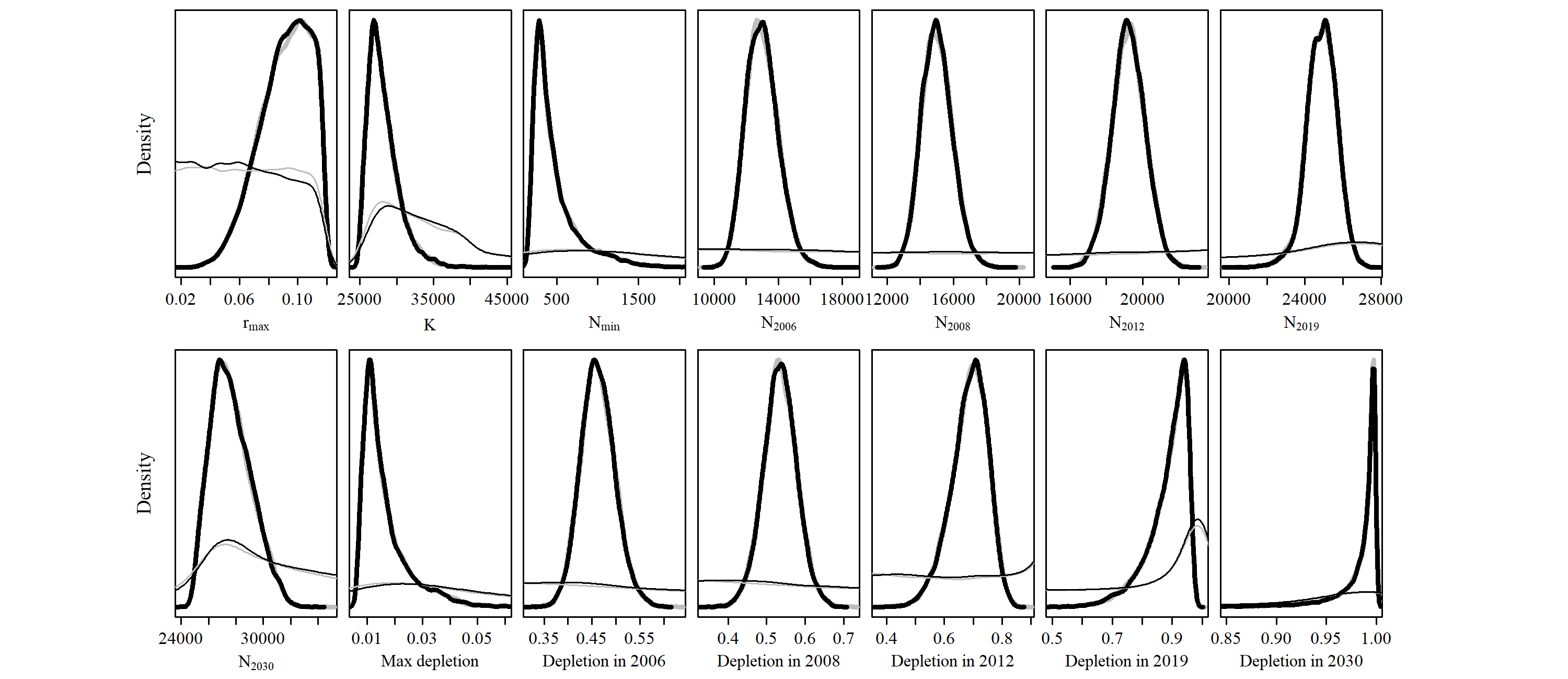
**

**5.18) Scenario G-2**

**
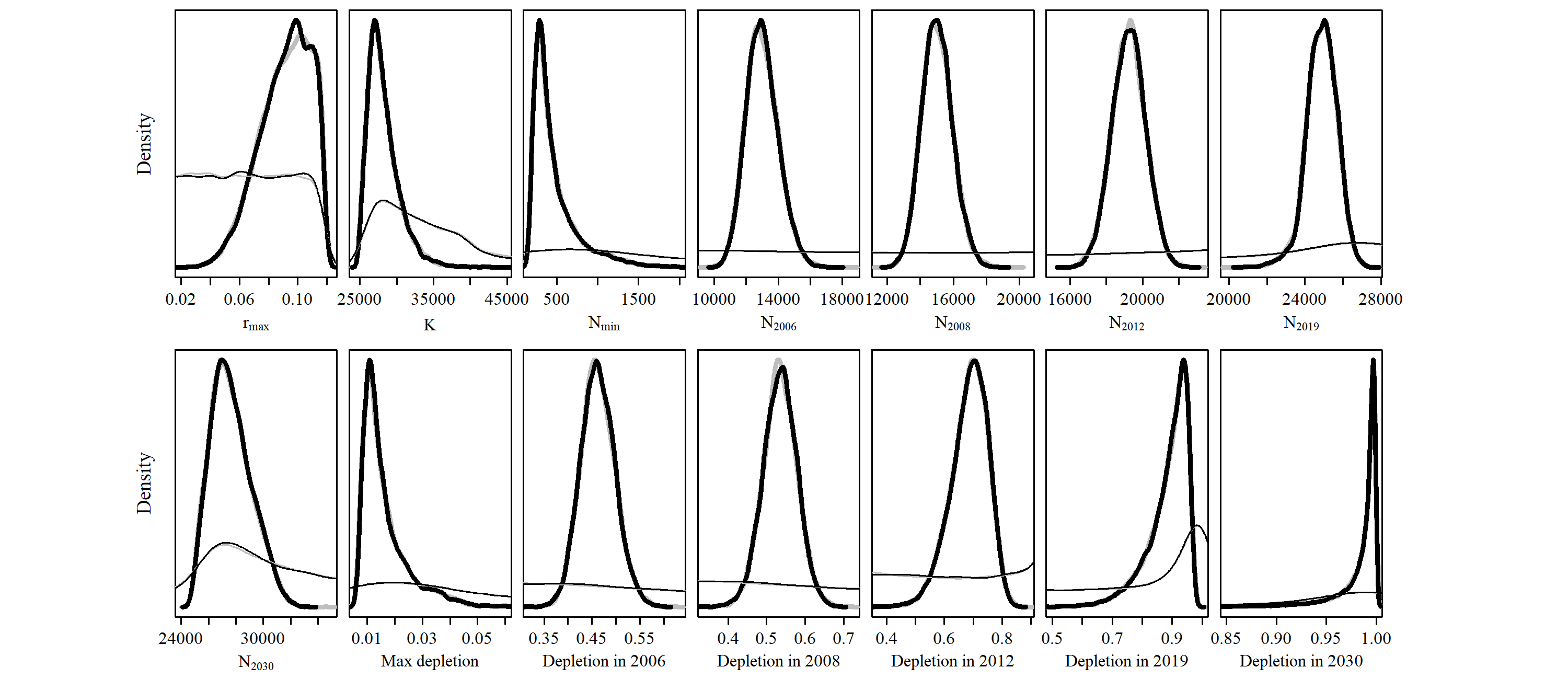
**

**5.19) Scenario M-1**

**
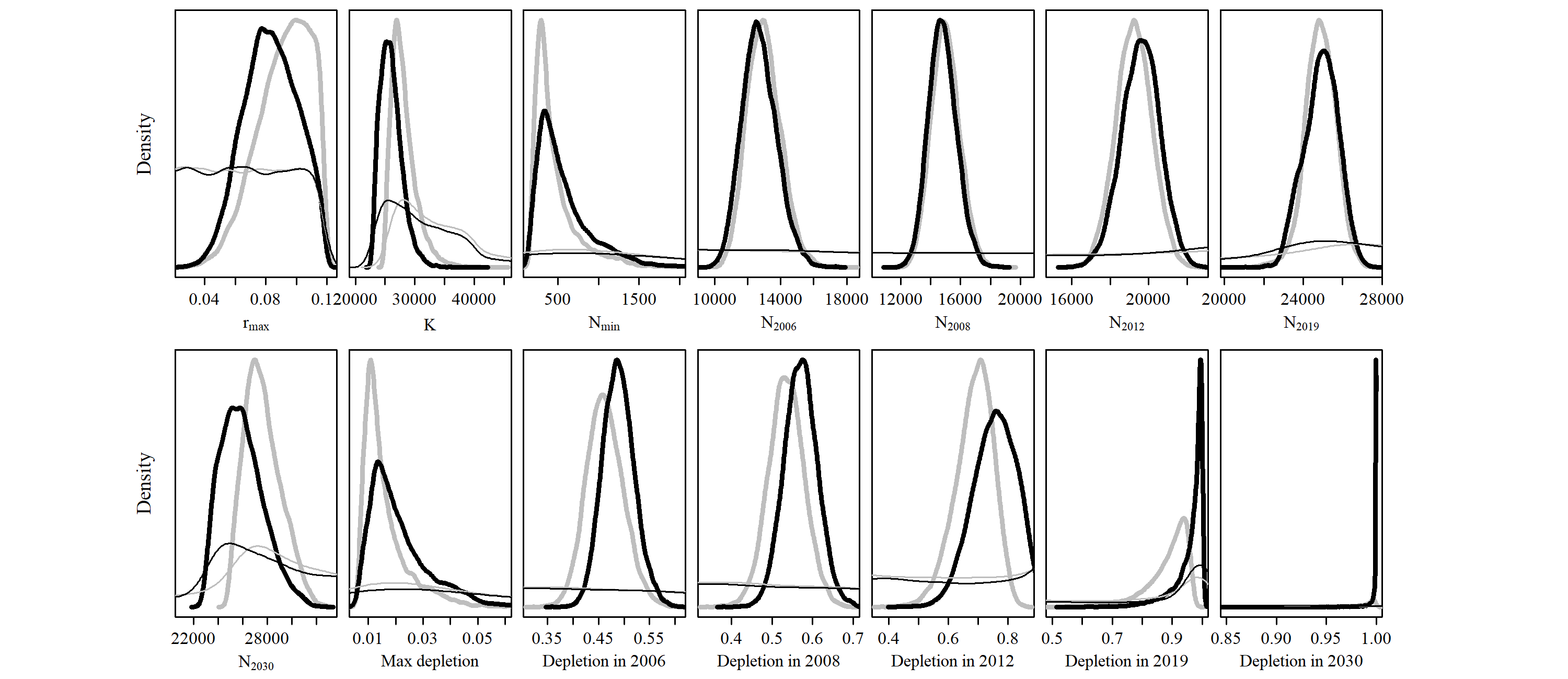
**

**5.20) Scenario M-2**


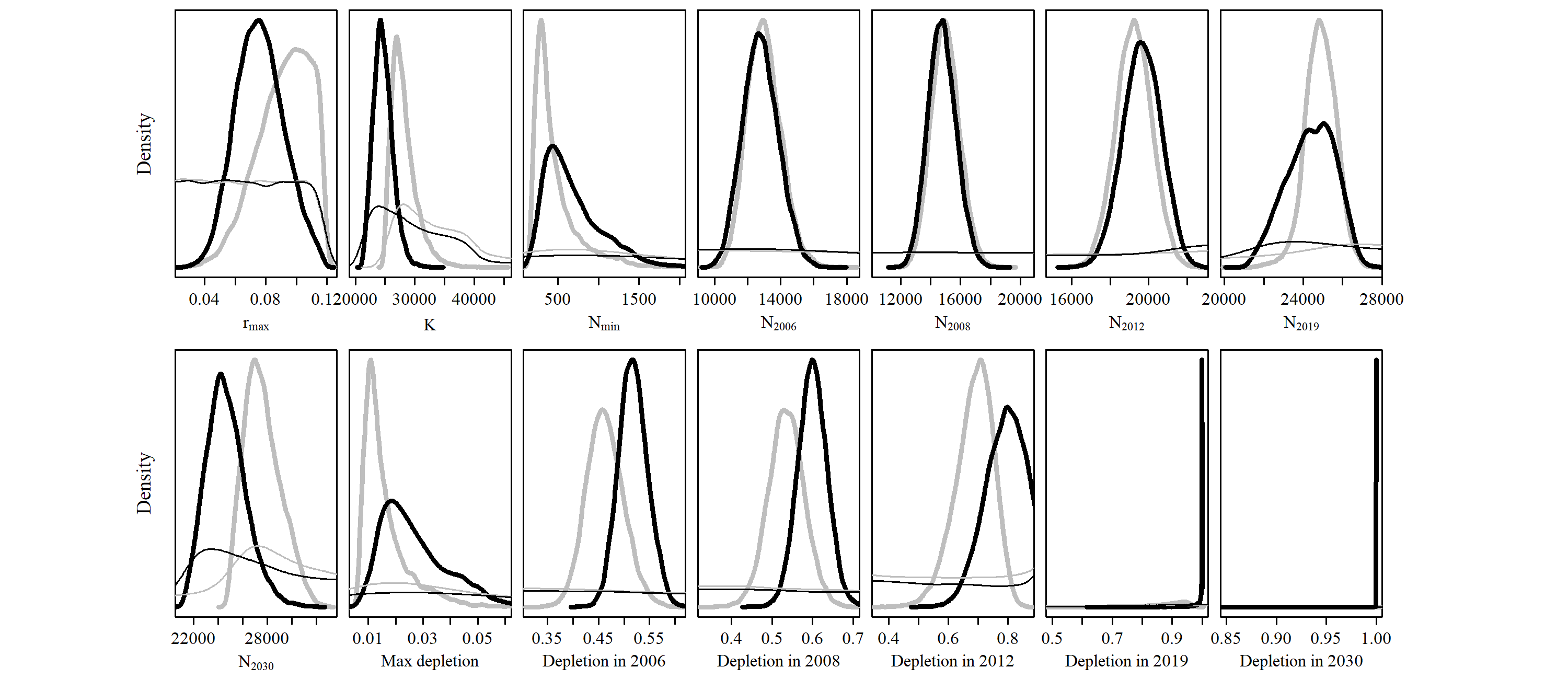

Supplement: Supplemental Material 2 [file rsos190368supp2.docx]
